# Ozone‐Tolerant Rice for Air‐Polluted Environments

**DOI:** 10.1111/gcb.70631

**Published:** 2025-12-08

**Authors:** Muhammad Shahedul Alam, Mirza Mofazzal Islam, Minh Khue Do, Blessing Omo Osimahon, Aishwarya Kushal Karki, Aleena Ittapad Baburajan, Marc Hartung, Sawitree Autarmat, Yanru Feng, Matthias Wissuwa, Michael Frei

**Affiliations:** ^1^ Department of Agronomy and Crop Physiology Justus‐Liebig‐University Giessen Germany; ^2^ Bangladesh Institute of Nuclear Agriculture (BINA) Mymensingh Bangladesh; ^3^ Institute of Crop Science and Resource Conservation (INRES) University of Bonn Bonn Germany

**Keywords:** air pollution, cereals, food security, molecular breeding, plant stress, yield losses

## Abstract

Recent decades have witnessed a surge in tropospheric ozone concentrations across many Asian countries, including Bangladesh, posing a significant threat to rice yields and regional food security. This study presents a comprehensive marker‐assisted breeding program designed to introgress two quantitative trait loci (QTL) conferring ozone tolerance into elite Bangladeshi rice varieties. We crossed the donor landrace Kasalath with high‐yielding varieties BRRI dhan28 and Binadhan‐11, generating BC_2_F_4_ lines with an average of 87% recipient parent genome recovery. These lines harbored diverse combinations of positive alleles at the target QTLs while retaining the agronomic traits of modern rice varieties. In controlled open‐top‐chamber experiments, the presence of these QTLs in elite backgrounds mitigated ozone stress symptoms, reduced physiological damage such as lipid peroxidation or loss in photosynthetic CO_2_ assimilation rates, and alleviated yield losses. Field trials conducted in a high ozone stress environment in Bangladesh, with and without the ozone protectant ethylenediurea (EDU), revealed that sensitive recipient parents suffered under ambient ozone concentrations. In contrast, the ozone‐tolerant lines exhibited significantly less yield loss and were less affected by pollution. Our findings demonstrate the efficacy of marker‐assisted breeding in adapting rice to changing atmospheric conditions, offering a robust strategy for safeguarding food security in the face of global change.

## Graphical abstract

To combat air pollution impacts, we introduced ozone tolerance into Bangladeshi rice varieties by marker‐assisted introgression of two quantitative trait loci (QTLs) from the landrace Kasalath. The resulting breeding lines displayed reduced stress symptoms and yield losses under elevated ozone conditions.

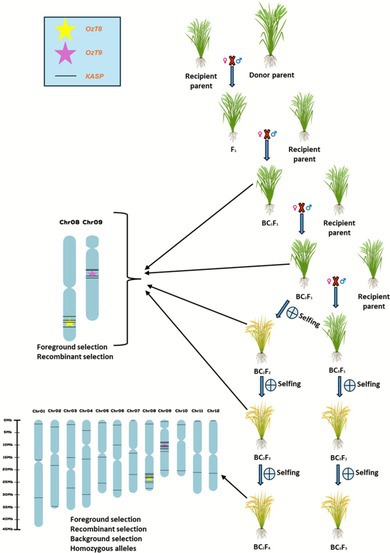

## 1 Introduction

Tropospheric ozone is a secondary air pollutant formed in the lower atmosphere through photochemical reactions involving precursor gases such as nitrogen oxides, carbon monoxide, and volatile organic compounds (The Royal Society 2008). Since its formation depends on intense solar radiation and high temperatures, ozone concentrations exhibit distinct diurnal (peaking in the late afternoon) and seasonal (peaking in summer) patterns (The Royal Society 2008). Recent satellite observations have shown that the Indian subcontinent has experienced the steepest rise in tropospheric ozone concentrations in recent decades and is now among the most polluted regions globally (Gaudel et al. 2024; Gopikrishnan and Kuttippurath 2024). Another study found that no other highly populated country has seen a greater increase in tropospheric ozone levels than Bangladesh (Brauer et al. 2016). This trend is attributed to the region's high population density and ongoing economic growth, which remain heavily dependent on fossil fuel combustion, a major source of ozone precursor gases (Akimoto 2003; Gao et al. 2020). The highest ozone concentrations in the Indian subcontinent are typically observed between March and May, when temperatures are high and cloud cover is minimal, allowing for intense solar radiation (Frei 2015; Gopikrishnan and Kuttippurath 2024). Elevated tropospheric ozone levels are harmful to human health, contributing to respiratory diseases. They also adversely affect natural vegetation and agricultural crops (The Royal Society 2008).

Ozone impairs plant performance by entering through the stomata during gas exchange related to photosynthesis (Ashmore et al. 2006). Once inside the mesophyll, ozone decomposes into reactive oxygen species (ROS), which cause oxidative stress and damage to plant tissues, including enzymes and lipids (Baier et al. 2005). Additionally, ozone can trigger an oxidative burst that initiates a cycle of cell death, resulting in necrotic lesions (Kangasjarvi et al. 2005). As a result, photosynthetic efficiency is reduced, leading to inhibited plant growth and significant yield losses in crops (Fuhrer 2009).

The extent to which ozone affects crop yields varies by species and the overlap between the crop's growing season and high ozone episodes. For the world's most widely cultivated crops, global yield losses due to ozone have been estimated for wheat (7.1%), soybean (12.4%), maize (6.1%) and rice (4.4%) (Mills et al. 2018). In recent years, the impact of ozone on rice has garnered increasing attention due to rising ozone levels in Asia, where rice is a crucial staple crop (Ainsworth 2008). Several important rice varieties grown in Bangladesh have been found to be particularly sensitive to ozone, showing yield losses of up to 40% in controlled ozone fumigation experiments (Akhtar et al. 2010; Ashrafuzzaman et al. 2017).

In our recent study, we monitored ozone concentrations in four major rice‐growing regions in Bangladesh and cultivated a selection of common Bangladeshi rice varieties with and without the application of the ozone protectant ethylenediurea (EDU) (Frei et al. 2024). This compound is sprayed on leaves to specifically protect plants from ozone damage and can thus be used to diagnose ozone stress in field conditions, where plants are exposed to ambient ozone stress (Agathokleous 2017; Ashrafuzzaman et al. 2018, 2017). The study revealed two key findings: first, that these rice‐growing areas are heavily polluted with tropospheric ozone, with concentrations exceeding the damage threshold of 40 ppb throughout much of the growing season; and second, that nearly all tested rice varieties showed significant yield improvements when treated with EDU, indicating that they were severely affected by ambient ozone levels. However, the widespread use of EDU as a foliar spray is not a practical solution for farmers due to its high labor demands, cost, limited availability, and unknown effects on ecosystems and human health. Therefore, developing and adopting ozone‐tolerant rice varieties offers a more sustainable and feasible approach to mitigating ozone‐induced yield losses.

Although ozone concentrations have been steadily rising worldwide, their negative impact on agricultural crop yields is unlikely to be addressed through conventional crop breeding approaches for several reasons. First, elevated ozone levels are spatially and temporally variable, depending largely on weather conditions and the emission of precursor gases. As a result, the selection of new crop varieties is unlikely to occur under consistently high ozone exposure (Ainsworth 2016; Frei 2015). Moreover, the significant rise in ozone levels across Asia has only occurred in recent decades—after many potential donors of ozone tolerance traits, such as landraces or wild rice relatives, had already been excluded from the gene pools typically accessed by breeders, who tend to focus on elite breeding lines. Therefore, targeted screening and breeding specifically for ozone tolerance are urgently required.

Despite the importance of this issue for global food security, ozone tolerance is rarely considered in current breeding programs. However, various studies have demonstrated intraspecific variation in ozone tolerance in key crops such as wheat (Begum et al. 2020; Biswas et al. 2008) and soybean (Burkey and Carter Jr 2009; Burton et al. 2016), which is a prerequisite for effective breeding. In rice, the screening of hundreds of varieties under controlled ozone conditions has revealed substantial variation in adaptive responses (Frei et al. 2008; Sawada and Kohno 2009; Ueda, Frimpong, et al. 2015). In our previous studies, we investigated this genetic variability and identified two major quantitative trait loci (QTLs) associated with ozone tolerance, where the tolerant alleles originated from the landrace Kasalath belonging to the *aus* subspecies (Frei et al. 2008). *OzT8* was linked to the plant's ability to maintain high biomass production and photosynthetic performance under ozone stress (Chen et al. 2011). In contrast, *OzT9* was associated with reduced formation of necrotic lesions, which was traced to the suppressed expression of a gene known as *OZONE RESPONSIVE APOPLASTIC PROTEIN1* (*OsORAP1*) (Frei et al. 2010; Ueda, Siddique, and Frei 2015). In controlled ozone fumigation experiments, the presence of these QTLs—either individually or in combination—significantly mitigated rice yield losses (Wang et al. 2014), deterioration in grain quality (Jing et al. 2016), and reductions in the feed value of rice straw (Frei et al. 2011). However, all of these studies were conducted using the genetic background of the *Japonica* rice variety Nipponbare, a standard reference cultivar in rice genetics and molecular biology (Kawahara et al. 2013). While scientifically valuable, Nipponbare has no agronomic relevance in South Asia, the region currently most affected by ozone‐induced rice yield losses. Thus, future research and breeding efforts must focus on transferring these tolerance traits into locally adapted, high‐yielding *indica* varieties that are of practical importance to farmers in ozone‐impacted areas.

To this end, we conducted a comprehensive marker‐assisted selection program using two widely cultivated Bangladeshi rice varieties as recipient parents. BRRI dhan28 was grown on 11%–41% of Bangladesh's rice‐growing area during the irrigated dry season (BRRI 2025) and has shown ozone‐induced yield losses of up to 40% in our previous studies (Ashrafuzzaman et al. 2017). Additionally, Binadhan‐11 is a popular rice variety bred for submergence tolerance (IRRI 2021), but it has proven to be highly sensitive to ozone in controlled fumigation experiments (Ashrafuzzaman et al. 2020). In this study, we investigated the following hypotheses: (i) introgressing the QTLs *OzT8* and *OzT9* into modern Bangladeshi rice varieties through marker‐assisted selection will improve their ozone tolerance; (ii) the presence of one or both QTLs will differentially influence foliar gas exchange and stress symptom formation of the breeding lines; (iii) newly developed breeding lines will exhibit enhanced ozone tolerance in field conditions without compromising yield potential.

## 2 Materials and Methods

### 2.1 Breeding Line Development

To identify polymorphic SNPs across the genome, three parental lines were subjected to genotyping‐by‐sequencing (GBS). Suitable SNP markers were subsequently converted into KASP (Kompetitive Allele Specific PCR) markers (Table S1) following LGC Genomics' KASP assay design guidelines. Genotyping was performed using the commercial services provided by LGC Genomics (Middlesex, UK). The RFLP marker interval reported by Frei et al. (2008) was defined as the target region, encompassing 1.17 Mb (9.63–10.80 Mb on chromosome 9) in the *OzT9* region and 1.07 Mb (23.11–24.18 Mb on chromosome 8) in the *OzT8* region.

The first round of genotyping, encompassing foreground selection and recombinant selection, was conducted on 218 BC_1_F_1_ lines to identify those with Kasalath introgressions at *OzT8* and *OzT9*. Of these lines, 135 were derived from the cross (A) BRRI dhan28 × Kasalath, and 83 were derived from (B) Binadhan‐11 × Kasalath. Foreground selection for each QTL was performed utilizing one KASP marker at the left border and another at the right border. Furthermore, two KASP markers, situated approximately 1 Mb away from the left and right borders, were employed for recombinant selection (Table S1). This genotyping procedure successfully identified lines carrying heterozygous tolerant alleles at the QTL positions, with or without recombination.

To restore the recipient's parental background, the selected BC_1_F_1_ lines were backcrossed again to produce BC_2_F_1_ progeny. A second round of genotyping was conducted on 1034 BC_2_F_1_ lines using markers previously developed for identifying Kasalath segments at QTL positions. Of these 1034 BC_2_F_1_ lines, 551 were derived from the cross (A) BRRI dhan28 × Kasalath, and 483 from (B) Binadhan‐11 × Kasalath. These lines were further screened for the desired phenotype, applying criteria such as plant architecture resembling the respective recipient parent (height, erect leaves, and large panicles), low sterility, grain size, shape, and color (as Kasalath has a red grain pericarp), and reduced or no awns (Kasalath typically has pronounced awns). This genotyping procedure successfully identified lines carrying either a single QTL or both QTLs with heterozygous tolerant alleles at the respective positions, with or without recombination. Subsequently, a few selected lines underwent an additional backcross to develop the BC_3_F_1_ progeny. For the third backcross, plants were chosen based on genotyping results from the second round and phenotypic characteristics observed up to the flowering stage.

To advance and generate homozygous lines, 50 BC_2_F_1_ lines from cross A and 40 BC_2_F_1_ lines from cross B were selected. From each line, 48 seedlings were planted to grow BC_2_F_2_ plants and advanced to BC_2_F_3_ generation. Simultaneously, 167 BC_3_F_1_ lines (100 from cross A and 67 from cross B) were planted for advancement. During these stages, phenotyping was performed based on previously established criteria. Subsequently, 2300 BC_2_F_2_ lines (1378 from cross A and 922 from cross B) and 46 BC_3_F_1_ lines (25 from cross A and 21 from cross B) were genotyped using markers developed earlier (Table S1).

This genotyping procedure successfully identified lines carrying homozygous tolerant alleles at most of the targeted QTL positions. Following genotyping and phenotyping, 44 BC_2_F_2_ breeding lines from cross A and 30 BC_2_F_2_ lines from cross B were selected. From each line, 48 seedlings were planted to grow BC_2_F_3_ plants. Additionally, 10 BC_3_F_1_ lines (five from cross A and five from cross B) were selected for further advancement. From each of these lines, 48 seedlings were planted to grow BC_3_F_2_ plants, which were subsequently advanced to the BC_3_F_3_ generation. Phenotyping was performed based on previously established criteria. Following this, genetic markers (Table S1) that had been previously developed were used for genotyping a total of 2200 BC_2_F_3_ lines (1189 from cross A and 1011 from cross B) and 96 BC_3_F_2_ lines (54 from cross A and 42 from cross B). This genotyping procedure again successfully identified lines carrying homozygous tolerant alleles at the QTL positions. In parallel, initial screening experiments for ozone tolerance were conducted using the selected BC_2_F_2_ progeny. Breeding line identifiers consisted of the prefix MFOL (Michael Frei Ozone‐tolerant Lines) and a unique three‐ or four‐digit code assigned during genotyping and phenotyping.

Based on the genotypic data and initial screening experiments, 10 lines from cross A (eight from BC_2_F_3_ and two from BC_3_F_2_) and nine lines from cross B (seven from BC_2_F_3_ and two from BC_3_F_2_) were selected for the final experiment (Table 2). As part of the genotyping process, 41 KASP markers (Table S1) were developed (Hospital and Charcosset 1997), including four markers previously utilized outside the QTL regions. These markers were designed to target all chromosomes for comprehensive background selection. Additional plants from the selected lines were cultivated independently for further analysis. The selected lines were genotyped using the 41 KASP markers to characterize the background genome. Concurrently, two additional KASP markers for *OzT9* and one for *OzT8* were developed to facilitate recombinant selection within the QTL regions (Table S1). Foreground selection was subsequently reconfirmed using seven markers, four of which had been employed in the earlier stages of the genotyping process. The background genome recovery in the breeding lines was determined using the following formula:

$$\text{Genome Recovery}\;\left(\%\right)=\left(\text{Number of markers matching the recurrent parent}/\text{Total number of markers tested}\right)\times 100$$

This formula calculates the percentage of the recurrent parent's genome in the breeding lines.

A detailed protocol for the marker‐assisted breeding scheme is provided as Appendix S1.

### 2.2 Greenhouse Experiments

The first screening experiment was conducted in a climate‐controlled greenhouse between September 2022 and February 2023 at the University of Giessen, Germany. A total of 77 rice lines were evaluated in this study. These included the parental lines, 44 BC_2_F_3_ breeding lines derived from the BRRI dhan28 × Kasalath cross, and 30 BC_2_F_3_ breeding lines derived from the Binadhan‐11 × Kasalath cross. Seed were germinated in Petri dishes containing deionized water at 30°C in complete darkness for 2 days, following the protocol described by Ashrafuzzaman et al. (2018). The germinated seeds were subsequently transferred to the greenhouse. Petri dishes containing the seeds were replenished with one‐fourth strength modified Yoshida nutrient solution (pH 5.5) (Shrestha et al. 2018) and exposed to natural light in the greenhouse for 6 days. The experimental setup in the greenhouse included 40 quick pots (Herrman Meyer KG, Art.‐Nr.: 741239) arranged in trays (H. Nitsch & Sohn GmbH & Co. KG, Art. Nr.: 481091). Each tray contained 24 spaces, each with a volume of 400 cm^3^. The artificial soil mixture used in the pots consisted of a 3:1 ratio of Hawita F.‐E. Typ N (Hawita Gruppe GmbH, catalog number: 0104006) and Profile Porous Ceramic (PPC) Greens Grade (TURF Handels GmbH, catalog number: Profile Sport). Osmocote Exact 3–4 M fertilizer (Herrman Meyer KG, catalog number: 81416) was incorporated into the soil mixture at a rate of 4 g/L. Seven‐day‐old seedlings were transplanted into the quick pots in a semi‐randomized block design experiment. Four open‐top chambers (1.625 m in height) were constructed to maintain a uniform microclimate. Two chambers were designated for ozone treatment, while the remaining two were control chambers. Three seedlings of the same genotype were transplanted within each chamber, with 10 trays, accommodating 231 plants per chamber. The plants were irrigated twice weekly to ensure adequate water availability in each tray. Beginning 2 weeks after transplanting, 50 mL of a 15 g/L Peter Excel fertilizer solution was applied fortnightly to each tray until the plants reached maturity. Supplementary lighting was provided in the greenhouse from 7:30 a.m. to 4:30 p.m., ensuring a minimum photosynthetic photon flux density (PPFD) of 400 μmol m^−2^ s^−1^. The greenhouse maintained an average temperature of 26/21°C (day/night) and an average relative humidity of 73%.

At 32 days after transplanting (DAT), fully established plants in both ozone chambers were subjected to ozone treatment until 148 DAT, near the end of the growing season. The ozone treatment was applied daily at a target concentration of 85–90 parts per billion (ppb) for 7 h each day (9:00–16:00). Ozone was generated using the Ozone‐Generator AQUARIZON 1.0 (INNOTEC High Engineering GmbH, Panoramastr. 5, D‐76327 Pfinztal, Germany). The generated ozone was blown via a fan connected to a central pipe, which distributed the gas through perforated plastic pipes positioned above both ozone chambers. Ozone levels were monitored in real‐time using an ozone gas analyzer (Anseros ozone gas analyzer mp, Anseros Klaus Nonnenmacher GmbH, Tübingen, Germany). Additionally, the concentrations in the chambers were continuously measured at 3‐min intervals with an independent handheld ozone monitor (Series 500; Aeroqual Ltd., Auckland, New Zealand). The mean ozone concentration recorded in the ozone chamber was 91 ± 18 ppb (standard deviation), whereas the control chamber maintained a mean concentration of 27 ± 7 ppb.

In the second greenhouse experiment, a total of 22 rice lines were selected, including three parental lines: the donor parent Kasalath and the recipient parents. These comprised 10 lines derived from the BRRI dhan28 × Kasalath cross (eight BC_2_F_4_ and two BC_3_F_3_) and 9 from the Binadhan‐11 × Kasalath cross (seven BC_2_F_4_ and two BC_3_F_3_). The experiment was conducted between April 2023 and October 2023 in a climate‐controlled greenhouse at the University of Giessen's research station Weilburger Grenze. Seedlings were prepared as described above. For the cultivation of rice plants, the universal substrate Hawita F.‐E. Type N (Hawita Gruppe GmbH, Catalogue No. 0104006) was utilized. This substrate was mixed with Profile Porous Ceramic (PPC) Greens Grade (TURF Handels GmbH, Catalogue No. Profile Sport) in a 3:1 ratio. Additionally, Osmocote Exact 3–4 M (Herrman Meyer KG, Catalogue No. 81416) was incorporated at a concentration of 4 g L^−1^ of substrate to ensure the provision of essential nutrients. The experiment was conducted using a semi‐randomized block design. Eight open‐top chambers measuring 1.625 m height were constructed to provide a controlled microclimate. Four chambers were allocated for ozone treatment, while the remaining four served as controls. A total of 576 1‐L pots were used, each filled with the previously prepared plant substrate and placed in planting trays. The pots were arranged in the greenhouse, each chamber containing six planting trays, accommodating 88 plants (4 plants from each genotype) per chamber. Nine‐day‐old seedlings were transplanted into plant pots filled with substrate. Irrigation was carried out twice weekly to maintain saturated soil moisture levels in each planting tray. Fourteen days after transplantation, the initial fertilizer application was conducted using a 50 mL solution of Peters Excel fertilizer at a concentration of 15 g L^−1^, which was applied to each planting tray. This fertilization process was repeated at 2‐week intervals until the plants reached maturity. The greenhouse environment was supplemented with artificial lighting from 7:30 AM to 4:30 PM to achieve a minimum PPFD of 400 μmol m^−2^ s^−1^. The average day/night temperatures within the greenhouse were recorded at 30.09°C/20.95°C, respectively, while the average day/night relative humidity levels were 61.85% and 85.55%, respectively.

At 30 DAT, the fully established plants were subjected to ozone fumigation in all ozone chambers using the systems described above, continuing until 153 DAT. The target ozone concentration was maintained at 100–110 ppb for 7 h daily (9:00–16:00). The average recorded ozone concentration in the treatment chambers was 107.20 ± 19.52 ppb, while the control chamber had an average concentration of 48.62 ± 12.51 ppb.

### 2.3 Field Experiment

The experiment was conducted in Amtoli, Madokhola, Sreepur, Gazipur (latitude 24° 18′ 36″ N, longitude 90°44′05″ E), located in the Madhupur Tract (Agro‐Ecological Zone 28, AEZ‐28) of Bangladesh. The study was carried out during the irrigated (Boro) season of 2024, which is characterized as the dry season, primarily reliant on irrigated water. During this season, rice crops are typically transplanted from December to early February and harvested between April and June. Our experiment was conducted from January to June 2024. Seedlings were transplanted in the third week of January, and harvesting occurred between late April and early June, depending on genotype‐specific growth duration. The experiment included a total of 13 genotypes, comprising five breeding lines derived from the cross BRRI dhan28 × Kasalath, five breeding lines from the cross Binadhan‐11 × Kasalath, and three parental lines: BRRI dhan28, Binadhan‐11, and Kasalath.

Field preparation, fertilizer application, and field management were conducted following the Bangladesh Rice Research Institute (BRRI) guidelines. The seeds were soaked in a water container for 24 h. After soaking, the seeds were removed from the water, placed in gunny bags, and kept in a dark room to facilitate germination. Once germinated, the seedlings were pre‐grown in nursery beds. Meanwhile, the experimental fields were ploughed using a tractor, followed by laddering to ensure proper soil leveling. Weeds and stubbles were manually removed during field preparation to optimize soil conditions.

Thirty‐five‐day‐old seedlings were transplanted at a spacing of 20 cm × 15 cm, with one seedling per hill. Fertilizers were applied at the following rates: urea (270 kg ha^−1^) as a nitrogen source, triple superphosphate (115 kg ha^−1^) for phosphorus, muriate of potash (150 kg ha^−1^) for potassium, gypsum (75 kg ha^−1^) for sulfur, zinc sulfate (6 kg ha^−1^) for zinc, and boron (7 kg ha^−1^) for boron. The fertilizer application schedule included an initial installment at 12 days after DAT, consisting of one‐third of the total urea and the full doses of all other fertilizers. The remaining urea was applied in two split doses as topdressing at 30 and 45 DAT. Pesticides were applied as needed to minimize insect and pest infestations. Weeding was performed manually three times at 15, 30, and 45 DAT.

The experiment was designed as a split‐plot arrangement with three replicates. The main plot treatment included exposure to EDU with two levels: with and without EDU. The subplots represented different genotypes. Each subplot measured 1.5 m^2^ (1 m × 1.5 m) and included 33 randomly selected plants for yield estimation at harvest, while border plants were excluded from data collection.

Ambient ozone concentrations were monitored daily at the experimental sites from the day of transplanting to harvest. Measurements were taken between 9:00 AM and 5:00 PM (8 h per day) at 3‐min intervals using a handheld ozone monitor equipped with a data logger (Series 500; Aeroqual Ltd., Auckland, New Zealand). The average ambient ozone concentration during this period was 88.99 ± 31.10 ppb.

Starting 1 week after transplanting and continuing until harvest, all plants in the experimental plots were treated weekly with a 300 ppm solution of EDU. The solution was applied using a backpack pesticide sprayer, ensuring that all leaves were saturated during each application. EDU, which is not commercially available, was obtained from Prof. William J. Manning of the Stockbridge School of Agriculture, University of Massachusetts (Manning et al. 2011). Fresh EDU solutions were prepared weekly at each experimental location. Plants in the control (non‐EDU) treatment were sprayed with an equivalent volume of water to ensure consistency in application methods. EDU treatments were applied at weekly intervals from 28 January to 2 June 2024. The experiment was conducted during the dry season, when only a few very light rainfall events occurred, none of which were substantial enough to cause EDU wash‐off or photodegradation. Therefore, no additional EDU applications were required beyond the regular weekly schedule.

### 2.4 Phenotypic Measurements in Greenhouse Experiments

Physiological measurements were made during the greenhouse experiments. Visible symptoms of ozone stress, assessed as the leaf bronzing score (LBS), were recorded on the second fully expanded leaves of each plant, following the methodologies described (Frei et al. 2008; Ueda, Frimpong, et al. 2015). The LBS scale ranged from 0, indicating no visible ozone‐induced symptoms, to 10, representing severe damage across the entire leaf surface.

Measurements of the normalized difference vegetation index (NDVI) and Lichtenthaler index 2 (Lic2) were conducted using a Polypen RP410 instrument (Photon Systems Instruments, Drásov, Czech Republic) (Begum et al. 2020). The NDVI was calculated using the formula NDVI = (*R*_780_ – *R*_630_)/(*R*_780_ + *R*_630_), where *R*_780_ and *R*_630_ represent reflectance at wavelengths of 780 nm and 630 nm, respectively. Similarly, Lic2 was determined as Lic2 = *R*_440_/*R*_690_, with *R*_440_ and *R*_690_ denoting reflectance at 440 nm and 690 nm, respectively (Begum et al. 2020). Data were collected from the second youngest fully developed leaf.

A LI‐600 Porometer/Fluorometer (LI‐COR Inc., Lincoln, Nebraska, USA) was employed to measure stomatal conductance, the quantum efficiency of photosystem II (PhiPS2). The quantum efficiency of photosystem II (PhiPS2) was calculated using the formula PhiPS2 = (*F*_*m*_′ − *F*_*s*_)/*F*_*m*_′, where *F*_*m*_′ represents the maximum fluorescence under actinic light, and *F*_*s*_ indicates the steady‐state terminal fluorescence. Data were collected from the second youngest fully expanded leaf.

In the first greenhouse experiment, photosynthetic CO_2_ assimilation rates were determined at the grain‐filling stage, while in the second greenhouse experiment, measurements were conducted at the late flowering stage. Net carbon assimilating rates were measured using a LI‐6800 Photosynthetic Gas Exchange System (LI‐COR Inc., Lincoln, Nebraska, USA). Measurements were conducted on the second youngest fully expanded leaf between 9:00 a.m. and 4:00 p.m. at a constant PPFD of 400 μmol photons m^−2^ s^−1^, a CO_2_ reference concentration of 400 ppm, a leaf temperature of 22°C, a relative humidity of 60%, and a flow rate of 300 μmol s^−1^.

In the first greenhouse experiment, lipid peroxidation was measured at the tillering stage. To assess lipid peroxidation in different genotypes, shoots' malondialdehyde (MDA) content was determined by analyzing thiobarbiturc acid responsive substances as described (Hodges et al. 1999; Höller et al. 2014).

For yield analysis in the greenhouse, flowering was recorded at 50%, and plants were harvested at physiological maturity specific to each genotype. During harvest, plant height and panicle number were recorded. Subsequently, the harvested individual plants were dried in an oven at 50°C for 72 h. Additional agronomic traits, including the number of filled grains, unfilled grains (empty husks), grain yield, and straw biomass, were measured to assess overall performance.

### 2.5 Yield Determination in Field Experiment

In the field experiment, grain yield and straw yield data were collected following the harvest. The grains were separated manually through hand threshing and then sun‐dried to reduce moisture content. Similarly, the straw was sun‐dried to ensure consistent drying. After drying, the weights of both grains and straw were recorded. The recorded data were subsequently converted to units of tonnes per hectare (t ha^−1^). Additionally, the harvest index (HI) was calculated as a percentage to measure the partitioning efficiency of biomass into grains.

### 2.6 Statistical Analysis

Relative values were calculated as the ratio of measurements under ozone stress conditions to those under control conditions. For the analysis of variance (ANOVA), the experimental data were subjected to a mixed model analysis utilizing the package lme4 in R. Ozone treatment, genotype, and their interaction were designated as fixed effects, while block and/or chamber were designated as random effects. The Dunnett test was performed to compare the breeding lines with their corresponding recipient parents.

Principal component analysis (PCA) was analyzed using relative values calculated as the ratio of the trait value under the stress treatment to its value under the control. Additionally, data collected at various growth stages were averaged to compute relative values that reflect the overall responses of different genotypes throughout the growth period. PCA was performed in R using the FactoMineR package for analysis and the Factoextra package for visualization.

## 3 Results

### 3.1 Breeding Lines Show High Recipient Genome Recovery

**FIGURE 1:**
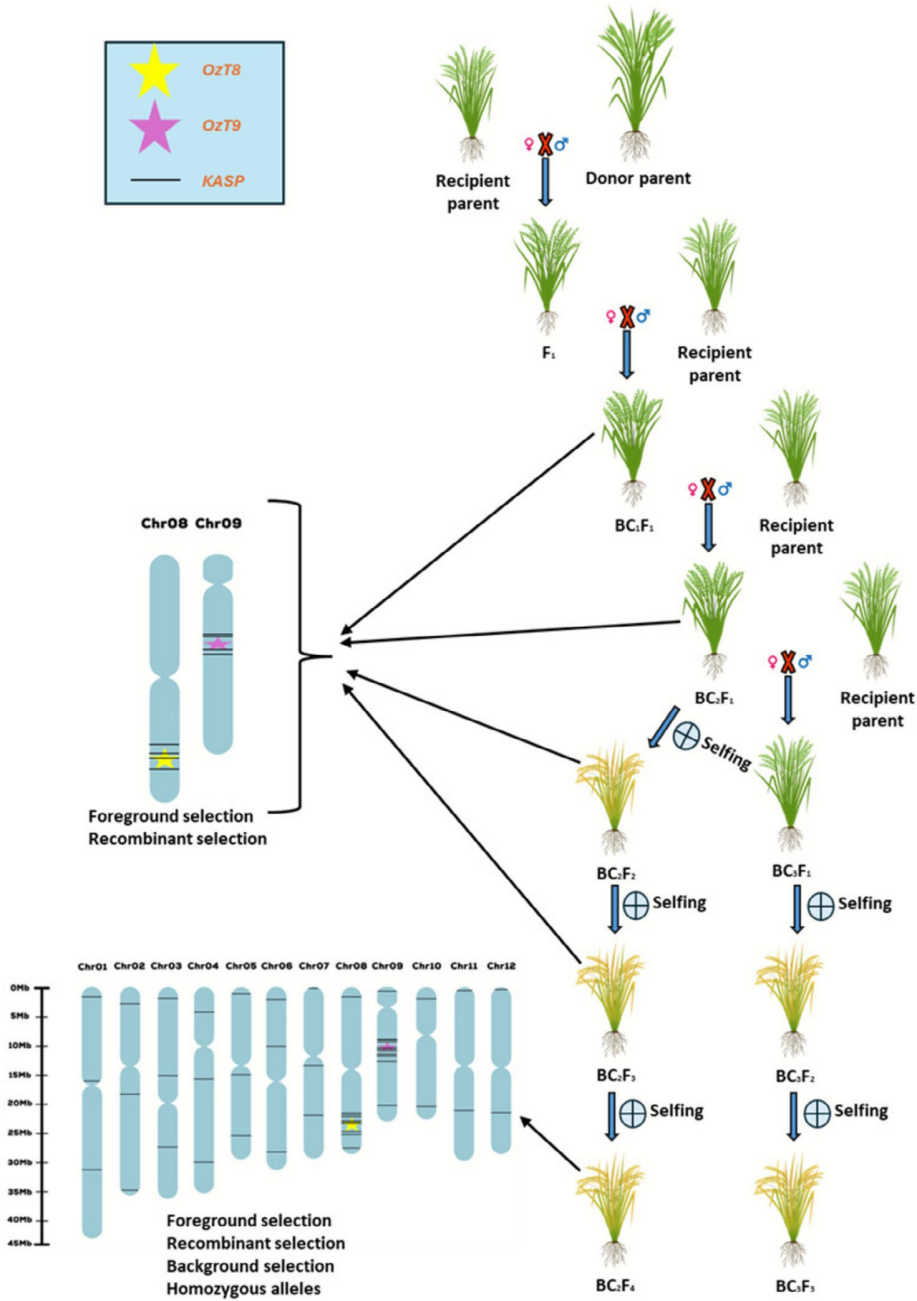
Schematic representation of the backcross breeding scheme for the introgression of ozone tolerance QTLs (*OzT8* and *OzT9*) into rice cultivars. The cross between the recurrent parent (BRRI dhan28 or Binadhan‐11) and the donor parent (Kasalath) was followed by backcrossing and selection for desired loci using marker‐assisted selection (MAS). The selection process integrates both phenotyping and genotyping. Foreground and recombinant selection were performed from BC_1_F_1_ to BC_2_F_4_ and BC_3_F_3_ progeny to identify favorable allelic combinations on chromosomes 8 and 9. Background selection was conducted on BC_2_F_4_ and BC_3_F_3_ progeny to evaluate the recovery of the recurrent parent's genetic background. Self‐pollination in advanced backcross generations ensured the fixation of homozygous alleles for the target loci. Chromosome diagrams highlight the positions of KASP markers, with *OzT8* (yellow star), *and OzT9* (pink star) explicitly indicated.

We introgressed two previously characterized QTLs conferring ozone tolerance, *OzT8* and *OzT9*, from the donor parent Kasalath, which belongs to the *aus* subspecies of rice into two recipient parents: (i) BRRI dhan28, a popular but ozone‐sensitive Bangladeshi rice variety belonging to the *indica* subspecies of rice, and (ii) Binadhan‐11, another modern Bangladeshi *indica* rice variety also sensitive to ozone (Ashrafuzzaman et al. 2020). Two parallel breeding schemes were implemented: (A) BRRI dhan28 × Kasalath and (B) Binadhan‐11 × Kasalath. The breeding process (Figure 1) commenced with initial crosses producing F_1_ progeny, followed by backcrossing to the respective recipient parents to generate BC_1_F_1_ progeny. Genotyping of the BC_1_F_1_ lines was performed using KASP markers to identify individuals carrying Kasalath introgressions at the *OzT8* and *OzT9* loci. Selected lines were advanced to the BC_2_F_3_ generation with continuous foreground and background selection employing KASP markers, along with phenotypic evaluation to select lines with suitable agronomic traits. This approach led to the selection of 74 lines for evaluation in the first ozone screening experiment: 44 BC_2_F_3_ lines derived from the BRRI dhan28 × Kasalath cross and 30 BC_2_F_3_ breeding lines derived from the Binadhan‐11 × Kasalath cross. These lines contained different combinations of either single or both QTLs (Tables S2 and S3). During the first screening experiment, we further evaluated lines for phenotypic traits, and excluded those exhibiting a spreading phenotype from further advancement and in‐depth physiological analyses. We focused only on lines that displayed an erect or moderately erect plant phenotype, similar to their recipient parents. This narrowed down the selection to 11 lines in the BRRI dhan28 × Kasalath background and 12 lines in the Binadhan‐11 × Kasalath background (Tables 1, S2 and S3). Furthermore, we excluded several lines from both BRRI dhan28 and Binadhan‐11 backgrounds, despite their strong performance across measured traits, due to agronomic unsuitability (Table 1). For the second round of experiments, we selected eight lines from the BRRI dhan28 × Kasalath cross and incorporated two new BC_3_F_2_ lines, while from the Binadhan‐11 × Kasalath cross we selected seven lines along with two new BC_3_F_2_ lines (Table 2). These selected lines exhibited an average genome recovery of 87.07% among the breeding lines originating from BRRI dhan28. Within this group, the genotype MFOL‐2236 exhibited a genome recovery rate of 82.93%, whereas MFOL‐233 demonstrated a higher recovery rate of 92.68% (Table 2). Similarly, breeding lines derived from the cross Binadhan‐11 × Kasalath exhibited an average genome recovery of 88.89%, with recovery rates ranging from 82.93% (observed in MFOL‐471 and MFOL‐50) to 95.12% (observed in MFOL‐1302) (Table 2). This variation indicates differential recovery rates across genotypes, with certain lines achieving closer proximity to the recurrent parent genome. These results highlight the efficiency of the background selection process in facilitating substantial recovery of the recurrent parent genome. Furthermore, the findings underscore the utility of KASP markers in background genotyping for marker‐assisted backcrossing, demonstrating their effectiveness in accelerating the development of improved breeding lines.

**TABLE 1:** Breeding lines selected for the first screening experiment and their phenotypic performance in comparison to their recurrent parents.

QTL	Line	Type	LBS	NDVI	Lic2	Phi PS2	SC	A	MDA
*BRRI dhan28 × Kaslaath*									
*OzT8*	MFOL‐2109	Erect	***	***	***	ns	ns	nd	***
*OzT8*	**MFOL‐2197**	Moderately erect	***	***	***	ns	ns	*	***
*OzT8*	**MFOL‐2236**	Erect	***	***	***	**	ns	*	***
*OzT9*	MFOL‐1926	Moderately erect	***	***	***	**	ns	nd	***
*OzT9*	**MFOL‐328**	Moderately erect	***	***	***	*	**	nd	***
*OzT9*	**MFOL‐1956**	Erect	***	***	***	ns	ns	**	***
*OzT8 + OzT9*	**MFOL‐1491**	Erect	***	***	***	ns	ns	**	***
*OzT8 + OzT9*	**MFOL‐1547**	Erect	***	***	***	ns	ns	***	***
*OzT8 + OzT9*	MFOL‐2388	Moderately erect	**	***	***	*	ns	***	***
*OzT8 + OzT9*	**MFOL‐1378**	Moderately erect	***	***	***	ns	ns	nd	***
*OzT8 + OzT9*	**MFOL‐1488**	Moderately erect	***	***	***	**	ns	nd	***
*Binadhan‐11 × Kasalath*									
*OzT8*	**MFOL‐1135**	Erect	***	***	***	*	ns	***	***
*OzT8*	**MFOL‐1198**	Erect	**	***	***	ns	*	***	***
*OzT8*	MFOL‐1281	Moderately erect	***	***	***	*	ns	nd	***
*OzT8*	MFOL‐1163	Moderately erect	***	***	***	***	ns	nd	***
*OzT9*	**MFOL‐1102**	Erect	ns	***	***	ns	ns	***	***
*OzT9*	**MFOL‐1011**	Erect	**	***	***	ns	ns	***	***
*OzT9*	**MFOL‐1302**	Erect	ns	**	***	ns	ns	nd	***
*OzT9*	MFOL‐1001	Moderately erect	***	***	***	ns	ns	nd	***
*OzT9*	MFOL‐1031	Moderately erect	***	***	***	*	ns	nd	***
*OzT8 + OzT9*	**MFOL‐471**	Moderately erect	**	**	***	ns	ns	***	***
*OzT8 + OzT9*	**MFOL‐544**	Moderately erect	ns	**	***	ns	*	***	***
*OzT8 + OzT9*	MFOL‐579	Moderately erect	***	***	***	ns	ns	nd	***

*Note:* “Type” indicates plant architecture. LBS leaf bronzing score at 80 days after start of ozone fumigation (DAO), NDVI normalized difference vegetation index at 80 DAO, Lic2 Lichtenthaler index 2 at 80 DAO, PhiPS2 quantum efficiency of photosystem 2 at 80 DAO, SC stomatal conductance at 80 DAO, A net CO_2_ assimilation rate at 116 DAO, MDA malondialdehyde concentration at 50 DAO. Asterisks indicate significantly improved relative (ozone/control) values of breeding lines compared to the recipient parent at *p < 0.05, **p < 0.01 and ***p < 0.001. Lines in bold were utilized in the second experiment. Selection focused on lines that performed significantly better than the recurrent parent under ozone stress, while also considering agronomic suitability (e.g., lodging, grain shattering).

Abbreviations: MFOL, Michael Frei Ozone‐tolerant Lines; nd, not determined; ns, not significant.

**TABLE 2:** KASP genotyping results for background, foreground, and recombinant selection in selected breeding lines with genome recovery percentages across 12 rice chromosomes. Parental genotypes are indicated in bold letters.

Marker	Kasalath	BRRI dhan28	MFOL‐2197	MFOL‐2236	MFOL‐1956	MFOL‐328	MFOL‐233	MFOL‐1378	MFOL‐1488	MFOL‐1491	MFOL‐1547	MFOL‐230	Binadhan‐11	MFOL‐1135	MFOL‐1198	MFOL‐1011	MFOL‐1102	MFOL‐1302	MFOL‐471	MFOL‐50	MFOL‐544	MFOL‐60
**Genome Recovery (%)**	**—**	**—**	85.37	82.93	90.24	87.80	92.68	85.37	87.80	87.80	85.37	85.37	—	92.68	90.24	92.68	90.24	95.12	82.93	82.93	85.37	87.80
*Chromosome 1*																						
**RIOZ_101**	**G:G**	**C:C**	C:C	C:C	C:C	G:G	C:C	C:C	C:C	C:C	C:C	C:C	**C:C**	C:C	C:C	C:C	C:C	C:C	C:C	C:C	C:C	C:C
**RIOZ_102**	**A:A**	**G:G**	G:G	G:G	G:G	G:G	G:G	G:G	G:G	G:G	G:G	G:G	**G:G**	G:G	G:G	A:A	G:G	G:G	G:G	G:G	A:A	G:G
**RIOZ_103**	**T:T**	**C:C**	C:C	C:C	C:C	C:C	C:C	C:C	C:C	C:C	C:C	C:C	**C:C**	C:C	C:C	C:C	C:C	C:C	C:C	C:C	C:C	C:C
*Chromosome 2*																						
**RIOZ_104**	**A:A**	**G:G**	G:G	G:G	G:G	G:G	G:G	G:G	G:G	G:G	G:G	G:G	**G:G**	G:G	G:G	G:G	G:G	G:G	G:G	G:G	G:G	G:G
**RIOZ_105**	**A:A**	**C:C**	C:C	C:C	C:C	C:C	C:C	C:C	C:C	C:C	C:C	C:C	**C:C**	C:C	C:C	C:C	C:C	C:C	C:C	C:C	C:C	C:C
**RIOZ_106**	**T:T**	**C:C**	C:C	C:C	C:C	C:C	C:C	C:C	C:C	C:C	C:C	C:C	**C:C**	C:C	C:C	C:C	C:C	C:C	T:T	C:C	C:C	C:C
*Chromosome 3*																						
**RIOZ_107**	**G:G**	**A:A**	A:A	G:G	A:A	A:A	A:A	A:A	A:A	A:A	A:A	A:A	**A:A**	A:A	G:G	A:A	A:A	A:A	A:A	A:A	A:A	A:A
**RIOZ_108**	**C:C**	**T:T**	T:T	T:T	T:T	T:T	T:T	C:C	T:T	T:T	T:T	T:T	**T:T**	T:T	T:T	T:T	T:T	T:T	T:T	T:T	T:T	T:T
**RIOZ_109**	**A:A**	**C:C**	C:C	C:C	C:C	C:C	C:C	C:C	C:C	C:C	C:C	C:C	**C:C**	C:C	C:C	C:C	C:C	C:C	C:C	C:C	C:C	C:C
*Chromosome 4*																						
**RIOZ_110**	**T:T**	**C:C**	C:C	C:C	C:C	C:C	C:C	C:C	C:C	C:C	C:C	C:C	**C:C**	C:C	C:C	C:C	C:C	C:C	C:C	C:C	C:C	C:C
**RIOZ_111**	**G:G**	**A:A**	A:A	A:A	A:A	A:A	A:A	A:A	A:A	A:A	A:A	A:A	**A:A**	A:A	A:A	A:A	A:A	A:A	A:A	A:A	A:A	A:A
**RIOZ_112**	**A:A**	**C:C**	C:C	C:C	C:C	C:C	C:C	C:C	C:C	C:C	C:C	C:C	**C:C**	C:C	C:C	C:C	C:C	C:C	C:C	C:C	C:C	C:C
*Chromosome 5*																						
**RIOZ_113**	**T:T**	**C:C**	C:C	C:C	C:C	C:C	C:C	C:C	C:C	C:C	C:C	C:C	**C:C**	C:C	C:C	C:C	C:C	C:C	C:C	C:C	C:C	C:C
**RIOZ_114**	**A:A**	**C:C**	C:C	C:C	C:C	C:C	C:C	C:C	C:C	C:C	C:C	C:C	**C:C**	C:C	C:C	C:C	C:C	C:C	C:C	C:C	C:C	C:C
**RIOZ_115**	**T:T**	**C:C**	C:C	T:T	C:C	C:C	C:C	C:C	C:C	C:C	C:C	C:C	**C:C**	C:C	C:C	C:C	C:C	C:C	C:C	C:C	C:C	C:C
*Chromosome 6*																						
**RIOZ_116**	**C:C**	**T:T**	T:T	C:C	C:C	T:T	T:T	T:T	T:T	T:T	T:T	T:T	**T:T**	T:T	T:T	T:T	T:T	T:T	T:T	T:T	T:T	T:T
**RIOZ_117**	**C:C**	**A:A**	A:A	A:A	A:A	A:A	A:A	A:A	A:A	A:A	A:A	A:A	**A:A**	A:A	A:A	A:A	A:A	A:A	C:C	A:A	A:A	A:A
**RIOZ_118**	**T:T**	**C:C**	C:C	C:C	C:C	C:C	C:C	C:C	C:C	C:C	C:C	C:C	**C:C**	C:C	C:C	C:C	C:C	C:C	C:C	C:C	C:C	C:C
*Chromosome 7*																						
**RIOZ_119**	**A:A**	**C:C**	C:C	C:C	C:C	C:C	C:C	C:C	C:C	C:C	C:C	C:C	**C:C**	C:C	C:C	C:C	C:C	C:C	C:C	C:C	C:C	C:C
**RIOZ_120**	**T:T**	**C:C**	C:C	C:C	C:C	C:C	C:C	C:C	C:C	C:C	T:T	C:C	**C:C**	C:C	C:C	C:C	C:C	C:C	C:C	C:C	C:C	C:C
**RIOZ_121**	**C:C**	**A:A**	A:A	A:A	A:A	A:A	A:A	A:A	A:A	A:A	A:A	A:A	**A:A**	A:A	A:A	A:A	A:A	A:A	A:A	A:A	A:A	A:A
*Chromosome 8*																						
**RIOZ_122**	**C:C**	**T:T**	T:T	T:T	T:T	C:C	T:T	T:T	T:T	T:T	T:T	T:T	**T:T**	T:T	T:T	T:T	T:T	T:T	T:T	T:T	T:T	T:T
**RIOZ_123**	**C:C**	**G:G**	C:C	C:C	G:G	G:G	G:G	G:G	C:C	G:G	G:G	G:G	**G:G**	G:G	C:C	G:G	G:G	G:G	G:G	G:G	G:G	G:G
**RIOZ_124**	**C:C**	**A:A**	C:C	C:C	A:A	A:A	A:A	C:C	C:C	A:A	A:A	C:C	**A:A**	C:C	C:C	A:A	A:A	A:A	A:A	C:C	A:A	A:A
**RIOZ_125**	**T:T**	**C:C**	T:T	T:T	C:C	C:C	C:C	T:T	T:T	T:T	C:C	T:T	**C:C**	T:T	T:T	C:C	C:C	C:C	T:T	T:T	T:T	T:T
**RIOZ_126**	**G:G**	**C:C**	G:G	G:G	C:C	C:C	C:C	G:G	G:G	C:C	C:C	G:G	**G:G**	G:G	G:G	G:G	G:G	G:G	G:G	G:G	G:G	G:G
**RIOZ_127**	**T:T**	**C:C**	T:T	T:T	T:T	T:T	T:T	T:T	T:T	T:T	C:C	T:T	**C:C**	T:T	T:T	C:C	C:C	C:C	T:T	T:T	T:T	T:T
**RIOZ_128**	**G:G**	**A:A**	G:G	G:G	A:A	A:A	A:A	G:G	G:G	G:G	G:G	G:G	**A:A**	G:G	G:G	A:A	A:A	A:A	G:G	G:G	G:G	G:G
**RIOZ_129**	**T:T**	**G:G**	T:T	T:T	G:G	G:G	G:G	G:G	G:G	T:T	T:T	T:T	**G:G**	T:T	G:G	G:G	G:G	G:G	T:T	T:T	T:T	T:T
**RIOZ_130**	**A:A**	**C:C**	A:A	C:C	C:C	C:C	C:C	C:C	C:C	A:A	A:A	A:A	**C:C**	C:C	C:C	C:C	C:C	C:C	C:C	A:A	C:C	C:C
**RIOZ_131**	**G:G**	**A:A**	G:G	A:A	G:G	A:A	A:A	A:A	A:A	A:A	G:G	A:A	**A:A**	A:A	A:A	A:A	A:A	A:A	A:A	A:A	A:A	A:A
*Chromosome 9*																						
**RIOZ_132**	**A:A**	**G:G**	G:G	G:G	G:G	G:G	G:G	G:G	G:G	G:G	G:G	G:G	**G:G**	G:G	G:G	G:G	G:G	G:G	G:G	G:G	G:G	G:G
**RIOZ_133**	**C:C**	**G:G**	G:G	G:G	G:G	C:C	G:G	G:G	G:G	G:G	G:G	G:G	**G:G**	G:G	G:G	G:G	G:G	G:G	G:G	G:G	G:G	G:G
**RIOZ_134**	**A:A**	**G:G**	G:G	G:G	G:G	G:G	A:A	A:A	G:G	G:G	G:G	A:A	**G:G**	G:G	G:G	G:G	G:G	G:G	A:A	A:A	A:A	A:A
**RIOZ_135**	**C:C**	**T:T**	T:T	T:T	C:C	T:T	C:C	C:C	T:T	C:C	T:T	C:C	**T:T**	T:T	T:T	C:C	T:T	C:C	C:C	C:C	C:C	C:C
**RIOZ_136**	**A:A**	**G:G**	G:G	G:G	A:A	G:G	A:A	A:A	G:G	A:A	G:G	A:A	**G:G**	G:G	G:G	G:G	G:G	A:A	A:A	A:A	A:A	A:A
**RIOZ_137**	**T:T**	**C:C**	C:C	C:C	T:T	C:C	T:T	T:T	C:C	T:T	C:C	T:T	**C:C**	C:C	C:C	T:T	C:C	T:T	T:T	T:T	T:T	T:T
**RIOZ_138**	**T:T**	**A:A**	A:A	A:A	T:T	T:T	T:T	T:T	T:T	T:T	A:A	T:T	**A:A**	A:A	A:A	T:T	A:A	T:T	T:T	T:T	T:T	T:T
**RIOZ_139**	**T:T**	**C:C**	C:C	C:C	T:T	T:T	T:T	T:T	T:T	T:T	T:T	T:T	**C:C**	C:C	C:C	T:T	T:T	T:T	T:T	T:T	T:T	T:T
**RIOZ_140**	**G:G**	**A:A**	A:A	A:A	G:G	A:A	G:G	G:G	G:G	G:G	G:G	G:G	**A:A**	A:A	A:A	G:G	G:G	G:G	G:G	G:G	G:G	G:G
**RIOZ_141**	**G:G**	**C:C**	C:C	C:C	C:C	C:C	C:C	C:C	C:C	C:C	C:C	C:C	**C:C**	C:C	C:C	C:C	G:G	C:C	C:C	C:C	C:C	C:C
**RIOZ_142**	**C:C**	**T:T**	T:T	T:T	T:T	T:T	T:T	T:T	T:T	T:T	T:T	T:T	**T:T**	T:T	T:T	T:T	C:C	T:T	T:T	T:T	T:T	T:T
*Chromosome 10*																						
**RIOZ_143**	**T:T**	**C:C**	C:C	C:C	C:C	C:C	C:C	C:C	C:C	C:C	C:C	C:C	**C:C**	C:C	C:C	C:C	C:C	C:C	C:C	C:C	C:C	C:C
**RIOZ_144**	**T:T**	**C:C**	C:C	C:C	C:C	C:C	C:C	C:C	C:C	C:C	C:C	C:C	**C:C**	C:C	C:C	C:C	C:C	C:C	C:C	C:C	C:C	C:C
*Chromosome 11*																						
**RIOZ_145**	**A:A**	**G:G**	G:G	G:G	G:G	G:G	G:G	G:G	G:G	G:G	G:G	G:G	**G:G**	G:G	G:G	G:G	G:G	G:G	G:G	G:G	G:G	G:G
**RIOZ_146**	**G:G**	**A:A**	A:A	A:A	A:A	A:A	A:A	A:A	A:A	A:A	A:A	A:A	**A:A**	A:A	A:A	A:A	A:A	A:A	A:A	A:A	A:A	A:A
*Chromosome 12*																						
**RIOZ_147**	**G:G**	**A:A**	A:A	A:A	A:A	A:A	A:A	A:A	A:A	A:A	A:A	A:A	**A:A**	A:A	A:A	A:A	A:A	A:A	A:A	A:A	A:A	A:A
**RIOZ_148**	**A:A**	**G:G**	G:G	G:G	G:G	A:A	G:G	G:G	G:G	G:G	G:G	G:G	**G:G**	G:G	G:G	G:G	G:G	G:G	G:G	G:G	G:G	G:G

*Note:* QTL positions are indicated in blue font, donor (Kasalath) genome fragments are shown in grey shading. The KASP markers with their sequences, used for genotyping in the marker‐assisted backcrossing process are provided in Table S1.

### 3.2 Breeding Lines Show Enhanced Physiological Tolerance and Less Reduction in Grain Yield in Ozone Fumigation Experiments

In a first screening experiment using open‐top chambers installed in a greenhouse, we evaluated the response of introgression lines generated through marker‐assisted backcrossing to ozone stress based on leaf symptoms and physiological parameters (Tables 1, S4 and S5). All lines, except three from the Binadhan‐11 × Kasalath cross, exhibited significantly lower symptom formation compared to the recipient parents. For other physiological traits, we compared the relative values (ozone/control) of the breeding lines to their respective recipient parents. In terms of vegetation indices representing foliar pigments, that is, NDVI and Lic2, all breeding lines outperformed their recipient parents. We further characterized photosynthetic performance by measuring chlorophyll fluorescence and gas exchange. These measurements revealed that some, but not all, breeding lines showed enhanced performance compared to their recipient parents. On the other hand, in terms of MDA concentration—a measure of oxidative stress—all breeding lines performed significantly better than their ozone‐sensitive parents. We refrained from analyzing and interpreting biomass and yield data in this experiment due to the dense planting of large numbers of lines in small pots. Instead, the results from this first screening experiment served as the basis for selecting lines for a second screening experiment with fewer lines, larger pots, and a higher number of chamber replicates.

**FIGURE 2:**
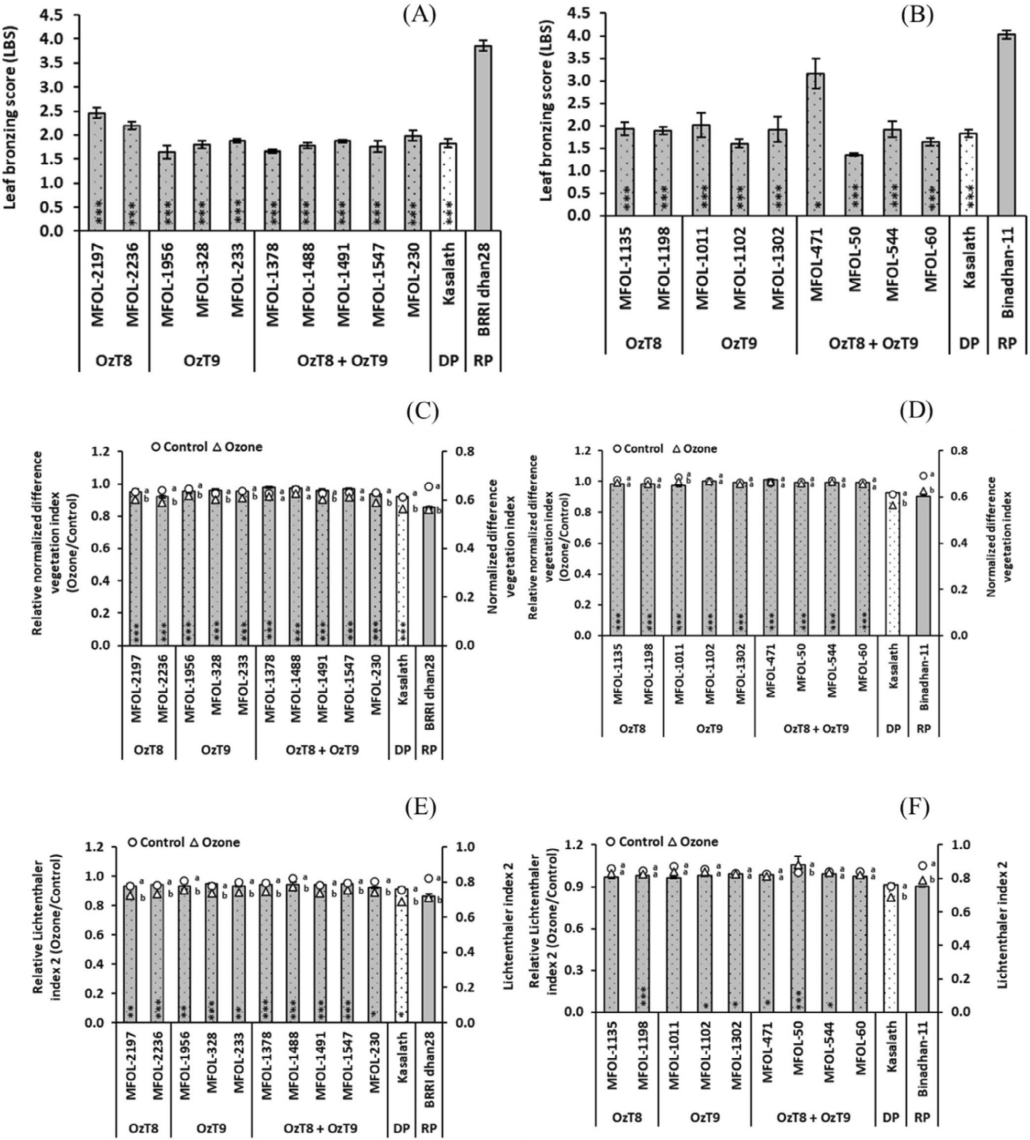
Foliar traits of selected breeding lines carrying *OzT8* and/or *OzT9* derived from the cross BRRI dhan28 × Kasalath (A, C, E) and Binadhan‐11 × Kasalath (B, D, F) under ozone stress and control conditions. (A, B) Leaf bronzing score (LBS) presented as absolute values; asterisks within the bars indicate significant differences between the genotypes and the recipient parent (BRRI dhan28 or Binadhan‐11), as determined by Dunnett's test (*p* < 0.05, *p < 0.01, **p < 0.001). (C, D) Normalized difference vegetation index (NDVI) and (E, F) Lichtenthaler index 2, presented as relative values (ozone/control). The bar graphs display the mean and standard error (*n* = 4). Circles (○) and triangles (△) represent absolute values for the control and ozone‐treated groups, respectively. Asterisks within the bars indicate significant differences between the genotypes and the recipient parent as determined by Dunnett's test, while letters shown beside the circles or triangles indicate significant differences among treatments within each genotype based on Šidák‐adjusted pairwise comparisons (*p* < 0.05). DP, donor parent (Kasalath), and RP, recipient parent (BRRI dhan28 or Binadhan‐11).

**FIGURE 3:**
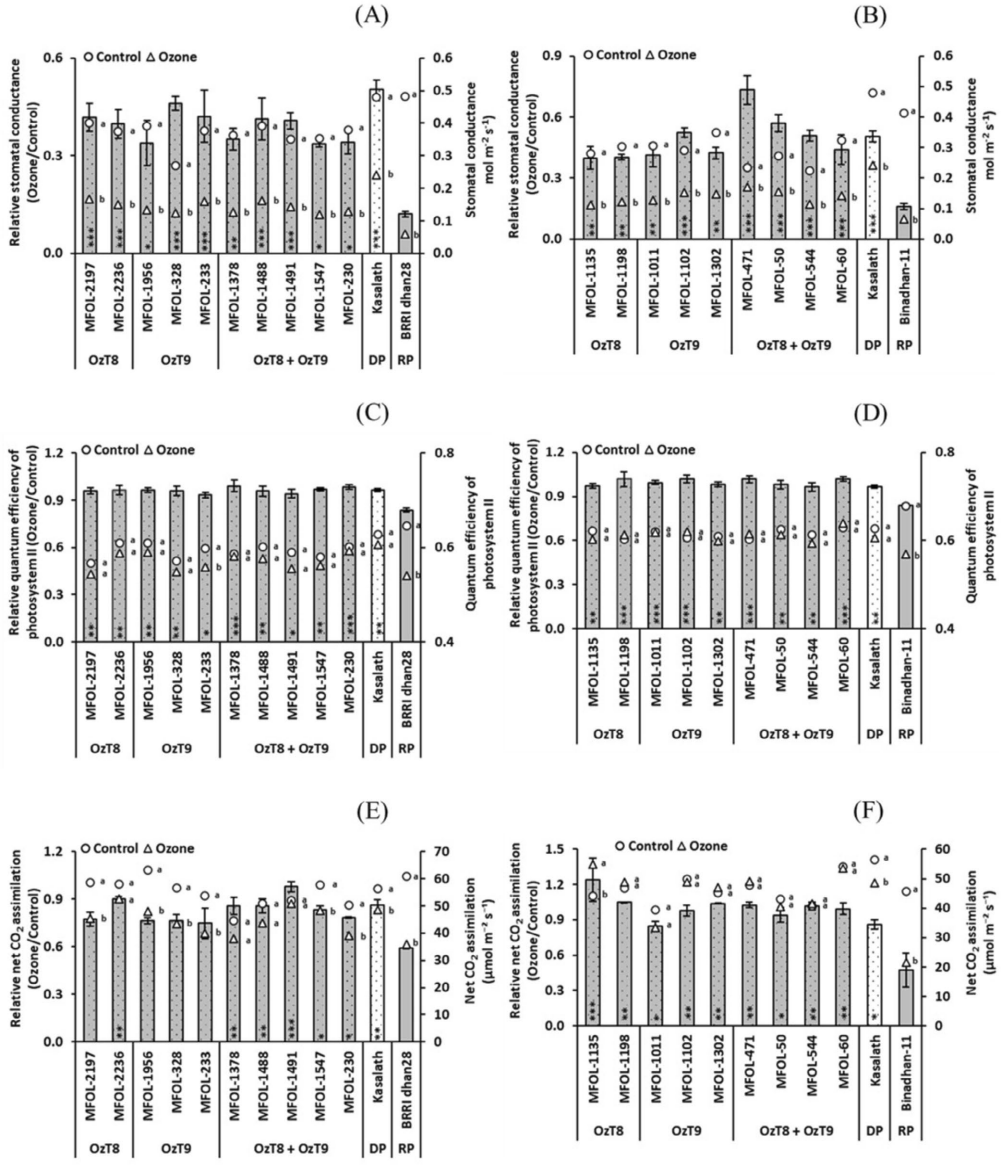
Photosynthetic parameters of selected breeding lines carrying OzT8 and/or OzT9 derived from the cross BRRI dhan28 × Kasalath (A, C, E) and Binadhan‐11 × Kasalath (B, D, F) under ozone stress and control conditions after 65 days of ozone fumigation. (A, B) Stomatal conductance, (C, D) quantum efficiency of photosystem II, and (E, F) net CO_2_ assimilation rate. The bar graphs display the mean and standard error of relative values (ozone/control, *n* = 4). Circles (○) and triangles (△) represent absolute values for the control and ozone‐treated groups, respectively. Asterisks within the bars indicate significant differences between the genotypes and the recipient parent (BRRI dhan28 or Binadhan‐11) as determined by Dunnett's test (*p* < 0.05, *p < 0.01, **p < 0.001). Letters shown beside the circles or triangles indicate significant differences among treatments within each genotype, based on Šidák‐adjusted pairwise comparisons (*p* < 0.05). DP, donor parent (Kasalath), and RP, recipient parent (BRRI dhan28 or Binadhan‐11).

In the second round of screening, we grew plants of the selected lines to maturity in open‐top chambers under ozone stress or control conditions and took physiological measurements during the growing season (Tables S6 and S7). As in the first round of screening, all breeding lines exhibited significantly lower symptom formation in both parental backgrounds (Figure 2A,B, Tables S10 and S11). This was also true for vegetation indices, particularly NDVI, although a few breeding lines did not outperform Binadhan‐11 for Lic2 (Figure 2E,F). We also evaluated photosynthetic and gas exchange traits. All breeding lines maintained relatively higher stomatal conductance and quantum efficiency of photosystem II under ozone stress compared to their recipient parents (Figure 3A,B). This was generally true for the net CO_2_ assimilation rate as well, except for four breeding lines from the BRRI dhan28 cross (Figure 3E,F). Remarkably, most breeding lines exhibited lower stomatal conductance and quantum efficiency of photosystem II than recipient parents under control conditions (Tables S10 and S11).

**FIGURE 4:**
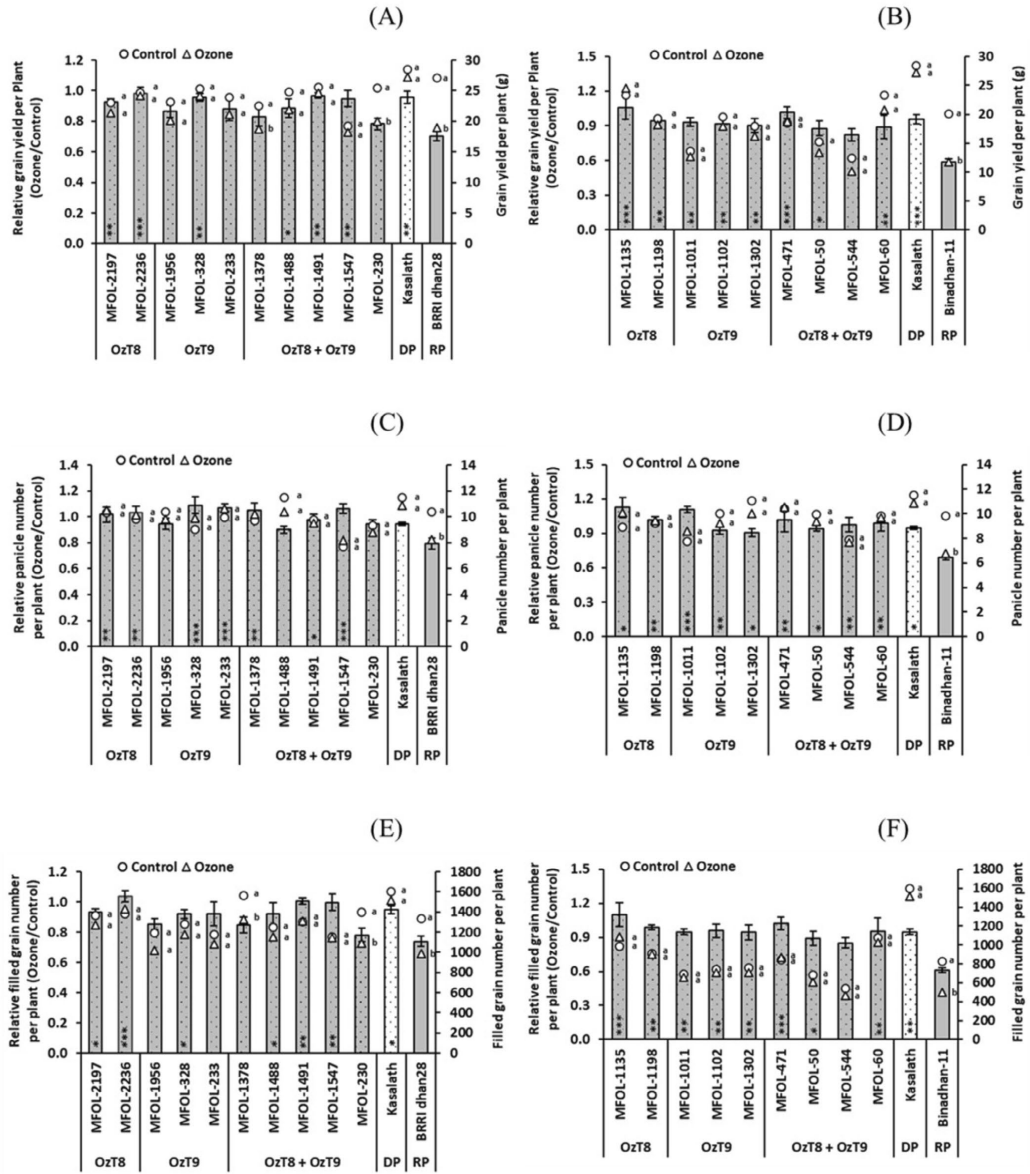
Grain yield components of selected breeding lines carrying *OzT8* and/or *OzT9* derived from the cross BRRI dhan28 × Kasalath (A, C, E) and Binadhan‐11 × Kasalath (B, D, F) under ozone stress and control conditions. (A, B) Grain yield, (C, D) panicle number per plant, and (E, F) filled grain number per plant. The bar graphs display the mean and standard error of relative values (ozone/control, *n* = 4). Circles (○) and triangles (△) represent absolute values for the control and ozone‐treated groups, respectively. Asterisks within the bars indicate significant differences between the genotypes and the recipient parent (BRRI dhan28 or Binadhan‐11) as determined by Dunnett's test (*p* < 0.05, *p < 0.01, **p < 0.001). Letters shown beside the circles or triangles indicate significant differences among treatments within each genotype, based on Šidák‐adjusted pairwise comparisons (*p* < 0.05). DP, donor parent (Kasalath), and RP, recipient parent (BRRI dhan28 or Binadhan‐11).

We further tested whether enhanced physiological performance would result in mitigation of yield losses under ozone stress (Tables S8 and S9). This proved to be the case for most of the breeding lines. While the two parental lines exhibited grain yield losses of −29.8% and −41.3% the ozone treatment, the yield changes in the breeding lines ranged between +5.7% and −21.8% (Figure 4A,B, Tables S12, S13, and S21). Importantly, the breeding lines did not differ significantly in grain yield potential from their recipient parental lines under non‐stress conditions. Their ability to maintain high grain yield was associated with enhanced performance in two yield components: while the recipient parents exhibited reduced panicle number (Figure 4C,D) and reduced filled grain number (Figure 4E,F) in the ozone treatment, these traits remained unaffected in all breeding lines.

**FIGURE 5:**
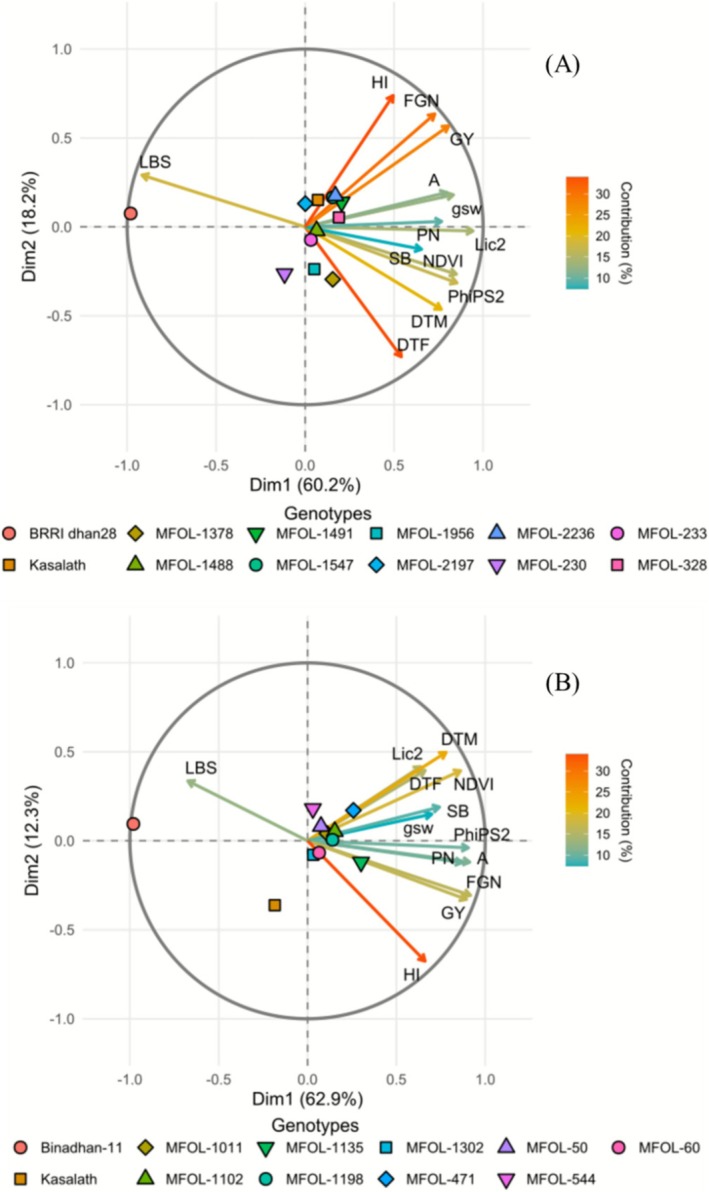
Principal component analysis (PCA) biplot showing the distribution of rice breeding lines carrying *OzT8* and/or *OzT9* derived from the cross BRRI dhan28 × Kasalath (A) and Binadhan‐11 × Kasalath (B) under ozone stress and control conditions. Cultivar distribution is presented along PC1 and PC2, with genotypes distinguished by shape/color and trait vectors color‐scaled by their contribution to the axes. The relative positions of genotypes reflect their ranking along the principal components (see Table S18), while trait loadings and contributions to PC1 and PC2 are detailed in Table S19, highlighting the phenotypic drivers of ozone sensitivity and resistance. Relative trait values (stress/control ratio) were used, except for LBS. Trait abbreviations: LBS, leaf bronzing score; NDVI, normalized difference vegetation index; Lic2, Lichtenthaler index 2; gsw, stomatal conductance; PhiPS2, quantum efficiency of photosystem II; A, CO_2_ assimilation rate; DTF, days to flowering; DTM, days to maturity; PH, plant height; PN, panicle number; FGN, filled grain number; GY, grain yield; SB, straw biomass; HI, harvest index. Sample size: *n* = 12 (A) and *n* = 11 (B).

To display the interrelations of physiological and yield traits in the selected breeding lines, we performed principal component analysis (Figure 5, Tables S14 and S15). In both genetic backgrounds, LBS and grain yield were strongly inversely related, demonstrating that foliar symptoms were negatively associated with yield. Conversely, high grain yield was associated with the ability to maintain physiological traits such as foliar pigment content (NDVI, Lic2) and photosynthetic performance.

### 3.3 Breeding Lines Show Superior Yield Performance in the Field

**FIGURE 6:**
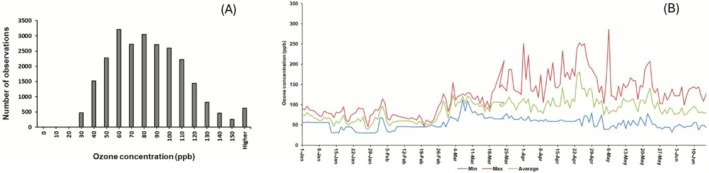
(A) Frequency distribution of ozone concentrations measured at the field site during the experimental growth period. Measurements were recorded at 3‐min intervals from 9:00 AM to 5:00 PM daily. (B) Seasonal dynamics of ozone concentrations (minimum, maximum, and average) during the field experiment.

Lastly, we evaluated selected breeding lines in a field experiment in Bangladesh. As fumigation experiments under field conditions would require costly and sophisticated infrastructure, such as Free‐Air Concentration Enrichment (FACE) systems, which are not available in Bangladesh, we opted to use ethylenediurea (EDU) as a tool to diagnose ozone response and sensitivity. The weekly application of a foliar spray of this chemical (Table S20) protects plants from ozone stress, thereby providing a ‘control’ under ambient ozone concentrations. The average daytime ozone concentration during the experimental period was high (89 ppb). In fact ozone values above the damage threshold for plants (typically considered as 40 ppb) occurred almost throughout the growing season, with peak episodes even exceeding 150 ppb (Figure 6). The ambient air occurring during the experimental period can thus be regarded as a high ozone stress environment.

**FIGURE 7:**
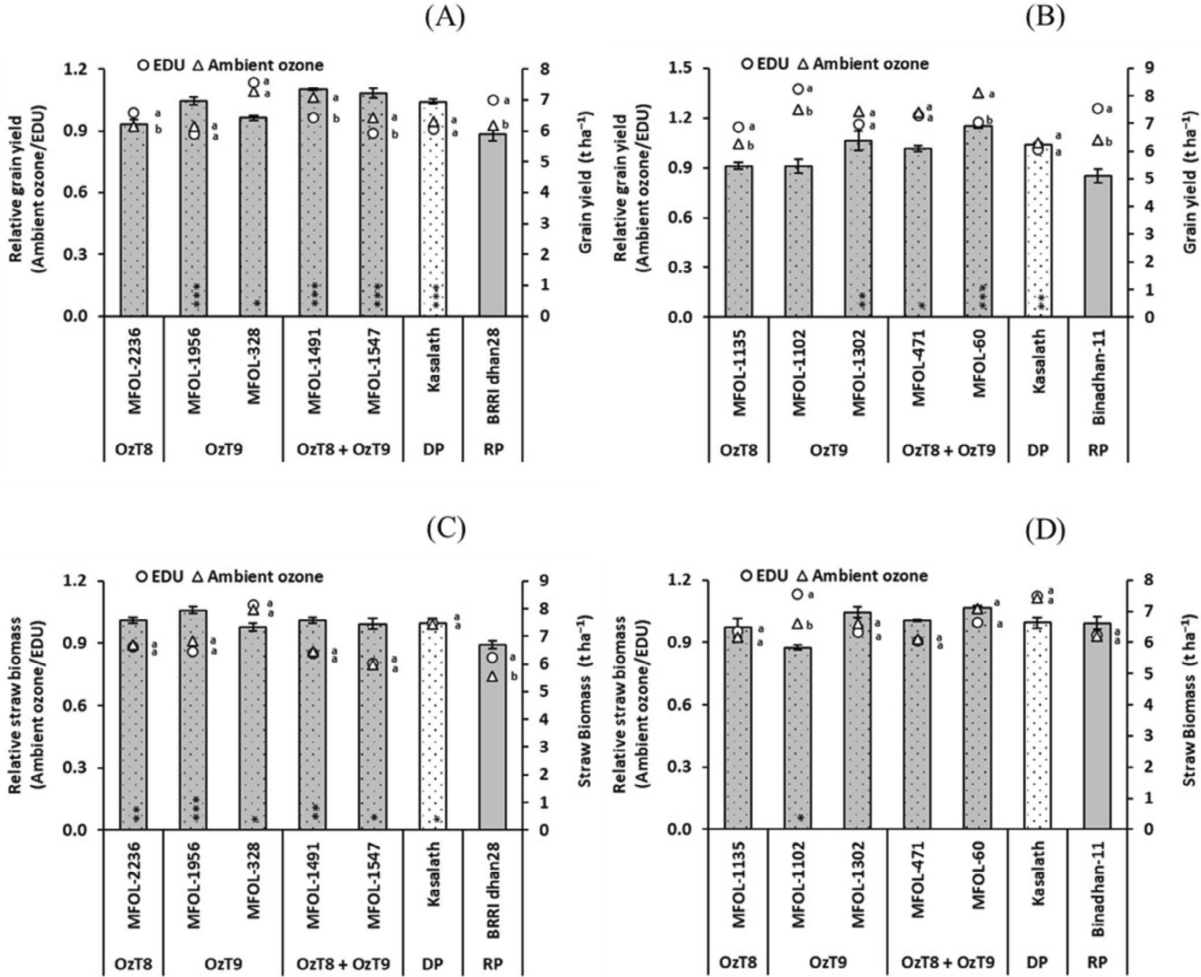
Grain (A, B) and straw (C, D) yield (t ha^−1^) of selected breeding lines carrying OzT8 and/or OzT9 derived from the cross BRRI dhan28 × Kasalath (A, C) and Binadhan‐11 × Kasalath (B, D) in field conditions under ambient ozone and with the application of the antiozonant ethylenediurea (EDU). The bar graphs display the mean and standard error of relative values (EDU/ambient ozone, *n* = 3). Circles (○) and triangles (△) represent absolute values for EDU‐treated and ambient ozone groups, respectively. Asterisks within the bars indicate significant differences between the genotypes and the recipient parent (BRRI dhan28 or Binadhan‐11) as determined by Dunnett's test (*p* < 0.05, *p < 0.01, **p < 0.001). Letters shown beside the circles or triangles indicate significant differences among treatments within each genotype, based on Šidák‐adjusted pairwise comparisons (*p* < 0.05). DP, donor parent (Kasalath), and RP, recipient parent (BRRI dhan28 or Binadhan‐11).

The EDU application in the experiment proved effective in protecting plants from ozone stress and resulted in a yield increase, especially in the ozone‐sensitive recipient parent lines. This led to a low ratio of grain yield in the ambient ozone/EDU treatment (Tables S16–S19, S22 and Figure 7A,B). However, most breeding lines exhibited significantly higher relative grain yield, reconfirming their ozone tolerance under field conditions. While the grain yield losses due to omission of EDU protection were −11.9% and −15.0% in the parental lines, the changes in the breeding lines were −9.1% to +15.1%. Importantly, this enhanced tolerance did not come at the cost of a constitutive yield penalty, as most lines exhibited similar yield potential to the recipient parents in non‐stress conditions (Tables S18, S19 and S22). In terms of straw yield (Figure 7C,D), the results were similar but with fewer significant differences from the parental lines. Overall, our results demonstrate the effective ozone tolerance of our breeding lines in the field without a yield penalty.

## 4 Discussion

Adaptive breeding for elevated tropospheric ozone requires genetic variation, which has previously been demonstrated and genetically dissected in important crop species such as, soybean (Burton et al. 2016), and wheat (Begum et al. 2020). However, to our knowledge, this is the first study reporting a marker‐assisted breeding (MAB) approach aimed at adapting any major crop variety to changing atmospheric conditions. Unlike biotechnological approaches such as genetic engineering or gene editing, MAB is more time‐consuming and laborious, as it requires multiple rounds of backcrossing and genotyping (Tester and Langridge 2010). On the other hand, it can be achieved through conventional crossing and is less controversially discussed in the public, posing fewer regulatory burdens for the release of new varieties (Agathokleous et al. 2023; Xiong et al. 2022). Following the development of breeding lines by MAB, we conducted experiments to address three distinct hypotheses.

### 4.1 Introgressing the QTLs *OzT8* and *OzT9* Into Modern Bangladeshi Rice Varieties Through Marker‐Assisted Selection Improves Ozone Tolerance

While a number of abiotic stresses related to global change, such as heat and drought, have received much attention and are widely considered in crop breeding schemes (Agathokleous et al. 2023), adaptation to changing atmospheric conditions through crop breeding remains largely unexplored. Only a few examples exist where cereal crops such as rice or wheat have been screened for adaptability to rising atmospheric CO_2_ concentrations (Agathokleous et al. 2023). Breeding efforts for ozone tolerance are similarly rare, partly because the screening for adaptability to atmospheric conditions is technically challenging and expensive, especially in the field where it typically requires FACE studies (Frei 2015). In this study, we took a different approach: we used quantitative trait loci (QTLs) that we had discovered and described in controlled fumigation experiments in open‐top chambers (Frei et al. 2010, 2008). Although a series of experiments demonstrated positive effects of the QTLs *OzT8* and *OzT9* on mitigating stress symptom formation (Frei et al. 2010), photosynthesis (Chen et al. 2011), grain yield losses (Wang et al. 2014), and grain quality under ozone stress (Jing et al. 2016), all previous studies had been conducted in the genetic background of the japonica variety Nipponbare, which is not agronomically relevant. This is a critical bottleneck for the utility of the QTLs, as there could be a genetic background effect implying a lack of functionality of the QTLs in widely grown and agronomically relevant *indica* varieties. Indeed a major weakness of many QTL mapping and gene cloning studies is that the effectiveness of the QTL is often only demonstrated in a narrow range of genomic backgrounds that have limited relevance to contemporary breeding programs (Cobb et al. 2019).

The marker‐assisted introgression of the QTLs *OzT8* and *OzT9* into elite *indica* varieties led to breeding lines with an overall background genome recovery of 87%, showing plant architecture similar to the recipient parent (Tables 1 and 2). Thus, we propose that the newly adapted lines would be suitable for current rice‐growing regimes. When introgressing stress tolerance into elite crop varieties, it is important to avoid a yield penalty under non‐stress conditions, as this might deter farmers from adopting new varieties that only perform better in unpredictable stress events (Mickelbart et al. 2015). On an individual plant level, the breeding lines developed in this study performed similarly to their parents (Figure 4), again suggesting that they form suitable material for further variety development. Thus, our study approach was successful in terms of QTL deployment into elite background with minimal linkage drag of undesirable traits from the donor landrace (Cobb et al. 2019). Under ozone stress, most lines exhibited superior grain yield performance compared to their recipient parent, indicating that their growth would be particularly warranted in ozone‐affected areas.

### 4.2 The Presence of One or Both QTLs Differentially Affects Foliar Gas Exchange and Symptom Formation of the Breeding Lines

In principle, ozone tolerance in plants can be achieved through stomatal regulation leading to effective exclusion, or through tissue tolerance, which implies biochemical acclimation or avoidance of oxidative stress despite high ozone uptake (Mills et al. 2018). However, exclusion can only be effective in the short term, as stomatal closure would not only exclude ozone but also inhibit photosynthetic gas exchange and thus plant growth. Consequently, the QTL employed in this study have previously been associated with tissue tolerance rather than exclusion. *OzT8* was proposed to confer biochemical acclimation of the photosynthetic apparatus to maintain high photosynthetic rates, rather than tolerance by stomatal closure (Chen et al. 2011). On the other hand, *OzT9* mitigated symptom formation and was associated with reduced expression of a gene, *OsORAP1*, which is involved in the oxidative cell death cycle in rice (Ueda, Siddique, and Frei 2015). Consequently, we would expect distinct and synergistic physiological effects of these two QTL. In this study, we developed breeding lines that contained either single or both QTL (Table 1). The ability to maintain high photosynthetic rates would be expected in breeding lines harboring *OzT8*. In fact, this trait was consistently seen in all breeding lines in the Binadhan‐11 background (Figure 3F), while in the BRRI dhan28 background, only the lines containing both QTL exhibited consistently superior performance (Figure 3F). Similarly, mitigation of leaf symptom formation would be expected in breeding lines harboring *OzT9*. Nevertheless, we observed mitigation of symptom formation in all breeding lines, even those containing only the QTL *OzT8* (Figure 2A,B). Thus, the stress‐mitigating effect of these QTL in the genetic background of ozone‐sensitive *indica* lines seems to be even more pronounced and nonspecific compared to their effect in the background of the moderately ozone‐sensitive japonica variety Nipponbare. Considering grain yield loss, the most important trait from a food security perspective, we observed superior performance of different breeding lines, irrespective of their QTL combination (Figure 4A,B). The presence of both versus only single QTL did not confer a significant advantage in our greenhouse study (Figure S3), while in the field experiment lines containing both QTL were significantly superior to those containing only *OzT8* (Figure S4). Notably, the best‐performing lines in the field contained both QTL (Figure 7A,B), suggesting that the pyramiding of these QTL optimized ozone tolerance in an *indica* background, as previously seen in Nipponbare‐derived lines (Wang et al. 2014).

### 4.3 Newly Developed Breeding Lines Exhibit Enhanced Ozone Tolerance in Field Conditions Without Compromising Yield Potential

The aim of developing stress‐tolerant crop varieties is typically to achieve yield stability in variable and heterogeneous stress situations without a yield penalty in non‐stress conditions (Mickelbart et al. 2015). This principle is highly relevant in the case of ozone stress, which depends on weather conditions and precursor gas emissions and is therefore rather variable (Ainsworth et al. 2008). The high susceptibility of widely grown rice varieties in Bangladesh, observed in this study (Figures 5 and 6) as well as previous investigations (Ashrafuzzaman et al. 2017, 2020), also demonstrates that breeders have not inadvertently selected for ozone tolerance despite strongly rising ozone concentrations in recent decades (Brauer et al. 2016). The spatial and temporal variability of ozone concentration may not have led to continuous selection pressure (Ainsworth et al. 2008). Therefore, a marker‐assisted breeding approach for the targeted introgression of tolerance QTL, while preserving the genetic background of the recipient parents, is warranted.

We tested the breeding lines developed in this study in ambient field conditions in Bangladesh, which, can actually be considered as a high ozone stress environments. Typically, ozone concentrations above 40 ppb are considered as damaging to plants, while the cumulative exposure to ozone is more harmful than individual episodes of ozone stress (Mills et al. 2007). Our ozone measurements clearly demonstrate the high and chronic level of ozone stress during the growing season, similar to our previous ozone monitoring in four rice growing areas of Bangladesh across 3 years (Frei et al. 2024). In these conditions, the breeding lines developed in this study exhibited grain yields between six and eight tonnes per hectare (Figure 7A,B). This compares to just above six tonnes per hectare for the recipient parents without the application of the ozone protectant EDU, while the average rice yield in Bangladesh in the year 2023 was 5.03 t per hectare (FAO 2025). Our data thus suggest that ozone tolerance does not compromise yield potential, although the breeding lines showed lower values in some physiological traits such as stomatal conductance and quantum efficiency of photosystem II in non‐stress condition (Figure 3). When comparing the yield in the EDU‐free control, where plants were exposed to ambient ozone without protection, our newly developed breeding lines even delivered between 4% and 27% more grain yield than the recipient parents. High yield potential of newly developed breeding lines was not associated with high relative yield losses due to ozone exposure (Table S22). We also selected these lines to show plant architecture suitable for modern agronomic schemes, that is, short and erect stature and non‐pigmented grain pericarps. We can therefore report successful QTL deployment into elite background, which forms an excellent resource for further variety development (Cobb et al. 2019). Based on the yield potential and ozone resistance, lines MFOL‐328, MFOL‐1491 in BRRI dhan28 background and MFOL‐60, MFOL‐1302 in Binadhan‐11 background appear most promising for further variety development.

In conclusion, we have shown that ozone tolerance QTL are highly effective when introgressed into the genetic backgrounds of ozone‐susceptible Bangladeshi elite varieties. The newly developed breeding lines serve as a valuable resource for cultivar development, and they can be readily crossed with additional elite varieties with minimal linkage drag of undesirable traits from the donor landrace Kasalath. This strategy presents a viable short‐ to mid‐term solution for protecting food security amidst elevated tropospheric ozone levels, but its success depends on local agricultural policies and farmer acceptance. Future research needs to address the stability of QTLs under varying ozone concentration gradients, their applicability in different genetic backgrounds, and the agronomic trait stability under long‐term cultivation. In addition, it is crucial to emphasize that the ultimate goal should remain the mitigation of air pollution through robust environmental protection measures.

## Author Contributions

Conceptualization: M.F., M.W., M.S.A., M.M.I.; Methodology: M.F., M.W., M.S.A.; Investigation: M.S.A., M.K.D., B.O.O., A.K.K., A.I.B., M.H., S.A., Y.F.; Data analysis and presentation: M.S.A.; Supervision: M.F.; Writing – original draft: M.F., M.S.A.; Writing – review and editing: B.O.O., M.W., M.M.I., M.K.D., A.K.K., A.I.B., M.H., S.A., Y.F.

## Funding

This work was supported by Deutsche Forschungsgemeinschaft, 426004147, 390732324.

## Conflicts of Interest

The authors declare no conflicts of interest.

## Supporting information


**Appendix S1:** gcb70631‐sup‐0001‐AppendixS1.docx.

## Data Availability

All raw data is available at https://doi.org/10.22029/jlupub‐20352.
