# Supplementary material for: Ozone‐Tolerant Rice for Air‐Polluted Environments
Source: Glob Chang Biol. 2025 Dec 8;31(12):e70631. doi: 10.1111/gcb.70631 (PMC12683472; doi:10.1111/gcb.70631)
Supplement: Supplementary file 1 — Appendix S1: gcb70631‐sup‐0001‐AppendixS1.docx. [file GCB-31-e70631-s001.docx]

Supplementary Materials for

**OZONE-TOLERANT RICE FOR AIR-POLLUTED ENVIRONMENTS**

Muhammad Shahedul Alam *et al.*

*Corresponding author. Email: [michael.frei@agrar.uni-giessen.de](mailto:michael.frei@agrar.uni-giessen.de)

**This PDF file includes:**

Supplementary Figures S1-S4

Supplementary Tables S1-S22

Supplementary Protocol for Marker-Assisted Breeding


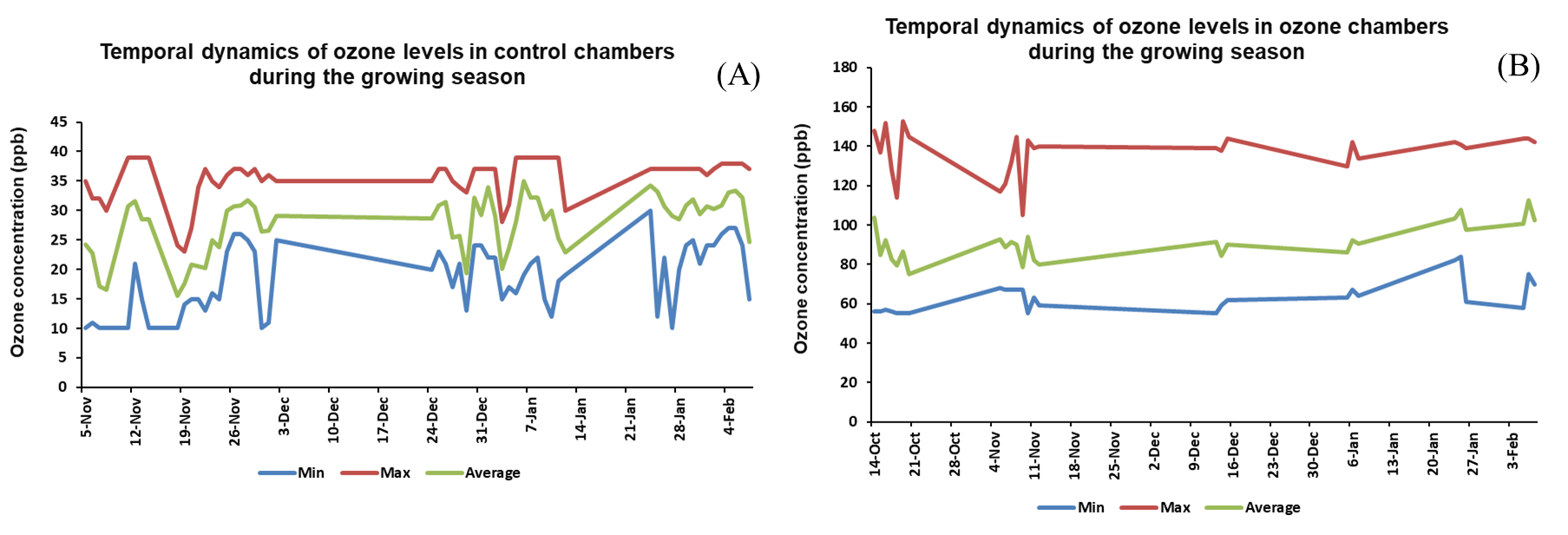


Supplementary Figure S1. Seasonal dynamics of ozone concentrations (minimum, maximum, and average) during the greenhouse experiment from September 2022 to February 2023. (A) Control chambers; (B) Ozone chambers.


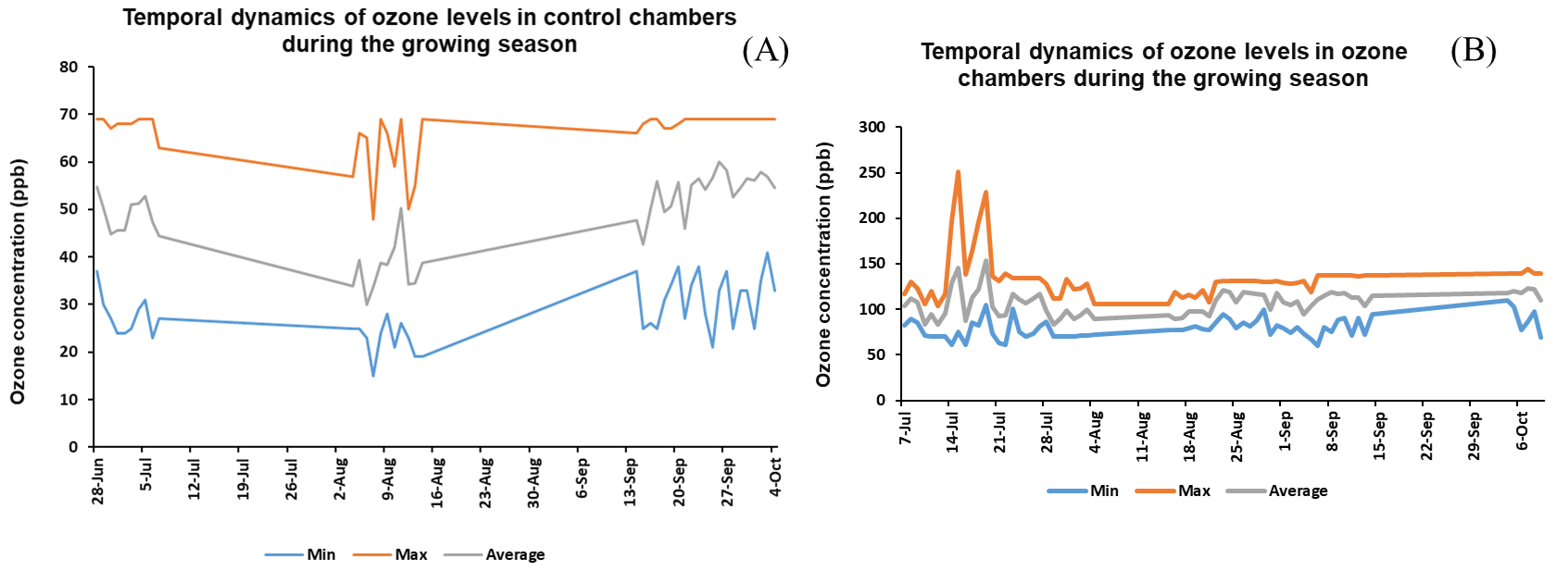


Supplementary Figure S2. Seasonal dynamics of ozone concentrations (minimum, maximum, and average) during the greenhouse experiment from April 2023 to October 2023. (A) Control chambers; (B) Ozone chambers.


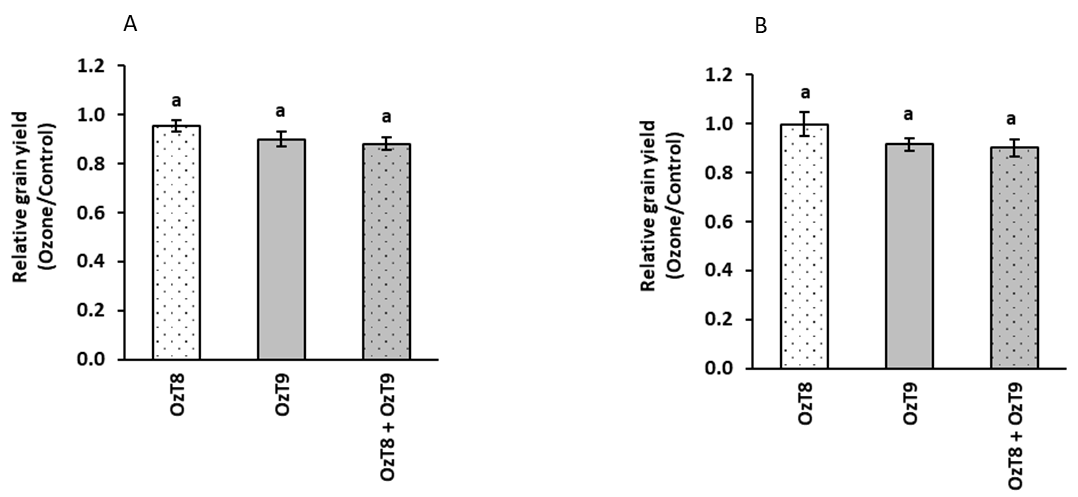


Supplementary Figure S3. Relative grain yield for different QTL combinations (*OzT8*, *OzT9*, and *OzT8* + *OzT9*) in response to ozone exposure (greenhouse condition) in breeding lines derived from (A) BRRI dhan28 × Kasalath and (B) Binadhan-11 × Kasalath. Bars labeled with the same letter are not significantly different according to Tukey’s HSD test (p < 0.05). Error bars represent the standard error of the mean.


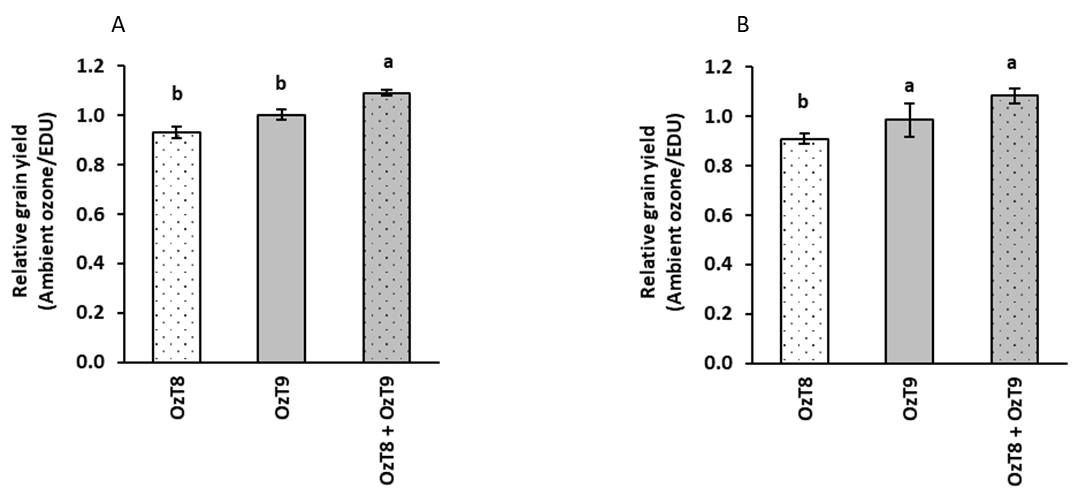


Supplementary Figure S4. Relative grain yield for different QTL combinations (*OzT8*, *OzT9*, and *OzT8* + *OzT9*) in response to ozone exposure (field trial) in breeding lines derived from (A) BRRI dhan28 × Kasalath and (B) Binadhan-11 × Kasalath. Bars labeled with the same letter are not significantly different according to Tukey’s HSD test (p < 0.05). Error bars represent the standard error of the mean.

**Table S1** Kompetitive Allele-Specific PCR (KASP) markers used for foreground, recombinant, and background selection.

| SNP ID | Sequence | Position | Purpose |
| --- | --- | --- | --- |
| RIOZ_101 | AAGTCCATGATACCATGCAGAGGCTTGTATAGTGAGAGTTAAGTCGTGAT[**C/G**]GAACAAGGACTACCTGGGCCCTTGGAGTTTGCTGTGCGGCCTTCTTTGAA | Chr1:1574479 | Background selection |
| RIOZ_102 | TTTTCGCTGAAGCATCACTTGCATCCTAAAAAAAATTAAAGTACCTGGGC[**G/A**]GCAAAGGCTGGGAGTGACGGCGAGTCAAGGATGTCGGTCGCTGGCAGAGG | Chr1:16348756 | Background selection |
| RIOZ_103 | ACCCGAATCACGAGGGCAGTTAAACGAAGGTGTCCAGCTGATTGTTGAGT[**C/T**]TCATGAACACATGAGATCGCACAAGGCCCAAGAACTCCACGTCAGGAGAA | Chr1:31792507 | Background selection |
| RIOZ_104 | tccttgaagacatatgtcatcggctagggtcatcactggacagtctggtg[**g/a**]ccgattgtggttcaatccaggcacgatttgcagaaatccaagctttaatc | Chr2:2472513 | Background selection |
| RIOZ_105 | TGAGCCCAGAGATCTGGGCGACGTTGACTCCTGTCACGGCCGATCCCTGC[**C/A**]TGTTGCTGGTGTTTCCAGCCATGTGAAGGCCGGAGAAGAAGAAGTCTTCC | Chr2:17165627 | Background selection |
| RIOZ_106 | ACTGGAGGCCCAACCTGTATTATGGGATGAGATCCCAAATGCAAAAATCA[**C/T**]ACAAGTTTGTCATGATTTTGTCGTAAGATCTGTCCTTTTACGAGTGGAAA | Chr2:35090950 | Background selection |
| RIOZ_107 | GAAAGCTGGTCTCCATACTTGATCCTTTGCAAACAAAACTAGAGGTAACC[**A/G**]TTGTGTTTTTATCCTTACTTTTCTAAATCTTTTTTCTGTCTTTGATGGTT | Chr3:1856562 | Background selection |
| RIOZ_108 | GCTGCAAATCTCCACCTATGGAAGTAATGCCAGAAGATGGTTTTACCATC[**T/C**]CTGGCTTAGCCTCTAGTTCTAAATCAAAATTATTAGCATTCAAATTATCC | Chr3:15217206 | Background selection |
| RIOZ_109 | AGAGCAATCGAAATGGCAGGGCAAGAAGGCCACGCCATGAAGGACAAGTT[**C/A**]GACAGGGGCATTGGATTTAAGGCCGCGGCCACCGCTGCGCATCATCCGGA | Chr3:27956992 | Background selection |
| RIOZ_110 | GAGCCCATGTTTACCACAACCTATCAAGTGAAGGCAATTATGAGTCAAAT[**C/T**]GATAAAGCCTCACTCGAGacaagagcttgaagctatgatgcatcaatctt | Chr4:4054239 | Background selection |
| RIOZ_111 | TAGCAAGACCAAAGTCGGCAAGTTTCACAGATCCATTCCTATGCACCAGT[**A/G**]TATTGCCACACTTCACATCCCTACAAGAATTTTGAACATTGAAGTACATG | Chr4:16023012 | Background selection |
| RIOZ_112 | AAAGAATGATGTGGATCGTCGTTTCAAATTCTCGAGTGTAAAAGGACAGG[**C/A**]CGAGTGGTTATCAATAATTAATTAGTGTAACCCTAAACCAAATGAAATGA | Chr4:30123004 | Background selection |
| RIOZ_113 | AACATGCCGTGCCCATGCTTGCCATGCTTGAACTTGCCATGGTGGCCGTG[**C/T**]TTGCCATGCTTGAACTTGCCGCCGTGGTGACCGCCGTAGCCATGGCCGTA | Chr5:1000902 | Background selection |
| RIOZ_114 | ccttaatatgaccaatcacattacaacgatgttcagggctatatttctcc[**c/a**]cacacttgaagcaaaggccctaagcacacctatagtatttcagctatcta | Chr5:14280112 | Background selection |
| RIOZ_115 | AGAGATACTATGCTTTTGTGTGAACATGCAGGTTATCCTGTGTTCTTCTT[**C/T**]GCCACTCGAGAATATTTACTCAGAGGTTTGTTTCTCACCTAATTGATTTT | Chr5:25256654 | Background selection |
| RIOZ_116 | GGAATAAGTGTTTTTGATTAAGAATGTAATGAATCCACATCATATATTTT[**T/C**]ACACATAAAAGAGCCAGAGTAACAATATACAATTTCGGCACCTGTTCTGT | Chr6:2028173 | Background selection |
| RIOZ_117 | atacccctgaggggttcagatcatcgaatcttagcgccaacacagccatg[**a/c**]tagtttacgaagccagaggatgcgaagcctactcgccaggagatctcgtc | Chr6:10801446 | Background selection |
| RIOZ_118 | GTGAGCCCGGGACCCAGAACAAGACCGGGTCCGAGGCATACCTTAGGCTA[**C/T**]ATTCTTAAGATATAGATTTTATCCACTAAACCTAAGGTCAGGATGGAAAT | Chr6:27039570 | Background selection |
| RIOZ_119 | TCTGTACGATTGGCAACTAGAATCTTGTACAGATCTACGGGGATACTGGC[**C/A**]GATCACAAAAACATATTGTTGGGGTTCCTGTCATCTTAGTTTGTGCCAAG | Chr7:372129 | Background selection |
| RIOZ_120 | CAGAGATGACACAGTGCTGTGACACAGTACTGAAGCTAAATCGGAGATGC[**C/T**]ATAGTCCTGTGACGTTGGATAAATCTCAACCATCCAATGCGCAGCAAACG | Chr7:13268038 | Background selection |
| RIOZ_121 | TTAGTACTGATCTGATAACTGAACTGTCAGAGAAACAGTAGAAGAGATAA[**A/C**]GCAACGACAGTTTCAGTTCCAGTTTCAGCTTTAATTTCCTCCATAATTTC | Chr7:22303126 | Background selection |
| RIOZ_122 | TTTTAGCTCAGATATACATTTGCGTGCTCGCTCAAAGAGATGTTTAGTGG[**T/C**]TGGGAGTTGTTTTGATTGAGATATGGCAGTTAATTTTCCCAGATCTGAGT | Chr8:1525984 | Background selection |
| RIOZ_123 | TAATAAAGTCTTCACATACTTTCTTGGACTGCCACAAATGTTGGATGCTC[**G/C**]CCGGAGACACCTAGTTGGCTAGGAGGATGACCTCCAAGATTATCAGCTCA | Chr8:21943017 | Background selection |
| RIOZ_124 | tgctattgtacttgctctcaGTGGTGGCTGCAACGCAGACAGASGGGCTA[**A/C**]YTTTCGCCGCGAAATTGTACTGCTACTACTGCTATCTTTGTGGTGGTTGA | Chr8:22179503 | Background selection |
| RIOZ_125 | AGGAGGACGAATGCTCTTGGTGTCAATCTCAAGTGCTCAAGGATGAATCT[**C/T**]GAACCTCACTTGCTTTTCAATCTAACTCAATCCAACTCAAAAGATTCACT | Chr8:22885079 | Recombinant selection |
| RIOZ_126 | ATATAGATCATTTTATCTTTCTCTTAATTTGCCTCTGACYTTTAGAATGA[**C/G**]AGTTGTTTTGGGAYGGAGGTCGTAGTATGTAACAACGGTATACTCCACGG | Chr8:23360411 | Foreground selection |
| RIOZ_127 | atttaatgcaaagattgtaatacttctccactaagttaggttgttgggcc[**c/t**]atgtggtaaccccttatggtataaaaggaggtmcaggcctacttagggag | Chr8:23538390 | Recombinant selection |
| RIOZ_128 | TCAATTTACAAATATTAGTTATCTACAAGCTTCCATTGCACACAAAGACA[**A/G**]GATAGCTTGGGGGAGAACTTGTGAAGTAATGGGTTCAATCCTTAGCCACG | Chr8:23845725 | Foreground selection |
| RIOZ_129 | CAGTATCAATAATGCATGCCAATGTTTAAGTGAAAAGATCTTTTATTTCC[**G/T**]CTGCGTTCGTTGTGTGGTGTATACCCCTACATGATAGTGGGGGAGTACTA | Chr8:24878875 | Recombinant selection |
| RIOZ_130 | GCCCGTAAAATATACTACTAATATGGGCTCATGGGCTGGTTGCTTCCAAT[**C/A**]TCTTTCGGTAGCCATAACACTGGGCCGCACCCTATGGGCCGATTCATGGG | Chr8:25434293 | Background selection |
| RIOZ_131 | TGGAAACACGGCTAGTTGAGTCAGAAAATGTCTGCATGATTGATCGCGTC[**A/G**]TTGCGTCCGATCCATTGCCGGAATAACAACGACCACGACTTACATCTATC | Chr8:27449978 | Background selection |
| RIOZ_132 | tttttagtctcggtttataagcgtaacaggattagatgatctttattccc[**g/a**]gttggggttaccaaccgggactaaagatcacaccaaccgagagtaaagat | Chr9:714308 | Background selection |
| RIOZ_133 | ATCTATCCAACAGCTTGTAACTTGGCAATGAATCTGGTAACAAGCTTGTA[**G/C**]CATCCAGCACAAAAAATGATTGGCTAACACAGATCTTGACTCAAAATGGA | Chr9:8995601 | Background selection |
| RIOZ_134 | tgcaagcgatcagaacAAACAATGGAGAATTCGTGGAGAGGCTGATCGTC[**G/A**]GTCTCTGTCTCGTCCTAATAGCGCASGAGGAGGATTCGCTTCTgtaacga | Chr9:9346103 | Recombinant selection |
| RIOZ_135 | CTACTAGATGAATTTGAGGACTGCAGTGAGGCCGGATCTCAAAGGGGCAG[**T/C**]TCCTGGATTGCAAAGGTTCGTCACAATCCTTTGAAAAGGGGTAACCCCCA | Chr9:9682191 | Foreground selection |
| RIOZ_136 | gatctcaggcaatggtgtggttctgaatgccctgctcgttccgatctctc[**g/a**]tgcgtgcaagcgatcaaaaccggggaagcagacagaagcagcacaggcta | Chr9:10282788 | Recombinant selection |
| RIOZ_137 | GGTTTATATATATTTATGATTATACTTTTGTATCACATACCCTCTTCGAT[**C/T**]CAAAATATAAGATTGTTTAGCATATAGATCTTTTCATTCTAAAATATAGG | Chr9:10364620 | Recombinant selection |
| RIOZ_138 | AACGCGGTATGCAAAGTTCCYRACCGTACCGATACCGTTTYCGYAAAACt[**a/t**]atataataataatatttttaCTGATGTTACCGTTTTCAAAATTTAGTTTT | Chr9:10499193 | Foreground selection |
| RIOZ_139 | GATATCCATGGAGGGCGGTGACTCCGGGTTTTTCAGGTTTTCGGCAGCGG[**C/T**]GCCGGTGCCARTGCCTGCACACCAGGCACGGAGGGCGCTATCAGATCTAC | Chr9:11247783 | Recombinant selection |
| RIOZ_140 | TAGTAGGGCACCAGCTGTTGAGGTTCAGGTCTCCTGCTTCTGCTGCTTTC[**A/G**]TGCAGCCTTTGGACCGTCAACATGCTGGGCTGTACGTTCAGTAAAGTAGG | Chr9:11838697 | Background selection |
| RIOZ_141 | AAGAGATCTCTCCTTCAAAATAGTACTAATAATCCATATCTTGATTAGAT[**C/G**]ACTCGGTCCACGGAGTGTACTAGGAGAATCAGAAGCGCCGTAGCGACTGT | Chr9:12077825 | Background selection |
| RIOZ_142 | TTTTCAACGTGAGAGATTCTTCCAGATCTTTCTTTCTTCTTCCTTAGGAG[**T/C**]AGTGATCCAAGATGATTGATTATTACGGCACGATATCAATCAATCATGCA | Chr9:20212811 | Background selection |
| RIOZ_143 | CAAAATTTTTAGGGCGATGCTCCGGTGCAATATACTTGAAGTATTCTACT[**C/T**]CGAGTAGAATTAATCTGGATCGTTTATTTTAAAGGGTATGTAAGTAATCT | Chr10:1666053 | Background selection |
| RIOZ_144 | CACGTTCGAAACAATCGCAAGATTGGCAAGTAAACTCTCCTGCTTGCTTG[**C/T**]TCACAACCACATCAGATCATTGATCAATGTTTCATCAGCTCATCACTTCT | Chr10:20377057 | Background selection |
| RIOZ_145 | gtcagttgcaggatcaaactgactggcacgcccgcatctcatcaaccttt[**g/a**]gacctgcattggagctaagcagatctcccagaccagtgtgtcatattttt | Chr11:509659 | Background selection |
| RIOZ_146 | GAAGAGGAAATAACAAAAGCAATTAGTCCACTTGTTGAAAAGTACTTTCC[**A/G**]AAGGAATCTTCTTCGGTATATAAGGTAACTTTCTGGCCTAATTTCTATGT | Chr11:21190408 | Background selection |
| RIOZ_147 | tcctgtgcaggtccaaaagttgttgagatgmgggcgtgccagtcagtttg[**a/g**]tcctgcaactgacaagatatgcaaatagtagatcaaacagccgatcggct | Chr12:239773 | Background selection |
| RIOZ_148 | GGCCTCTGAACTCGAGGAGTTCAGATCTTGTGCTCACGAGAGGTGGCAGC[**G/A**]ACTGCATCCAAAGGGGAAGGGGGAGAAGAATCTGTCATGTAGCTATGGTA | Chr12:21834365 | Background selection |

**Table S2** Morphological characteristics of breeding lines derived from the BRRI dhan28 × Kasalath cross.

| **QTLs** | **Line** | **Type** | **Remarks** |
| --- | --- | --- | --- |
| ***OzT8*** | MFOL-2149 | Spreading | X |
|  | MFOL-2218 | Spreading | X |
|  | MFOL-2248 | Spreading | X |
|  | MFOL-2163 | Spreading | X |
|  | MFOL-2109 | Erect, similar to BRRI dhan28 | Selected |
|  | MFOL-2181 | Spreading | X |
|  | MFOL-2179 | Spreading | X |
|  | MFOL-2090 | Spreading | X |
|  | MFOL-2102 | Spreading | X |
|  | MFOL-2197 | Moderately erect, close to BRRI dhan28 | Selected |
|  | MFOL-2204 | Spreading | X |
|  | MFOL-2236 | Erect, similar to BRRI dhan28 | Selected |
| ***OzT9*** | MFOL-2018 | Spreading | X |
|  | MFOL-1798 | Spreading | X |
|  | MFOL-1926 | Moderately erect, close to BRRI dhan28 | Selected |
|  | MFOL-2026 | Spreading | X |
|  | MFOL-1925 | Spreading | X |
|  | MFOL-1973 | Spreading | X |
|  | MFOL-96-325 | Spreading | X |
|  | MFOL-1881 | Spreading | X |
|  | MFOL-1778 | Spreading | X |
|  | MFOL-1801 | Spreading | X |
|  | MFOL-117-331 | Spreading | X |
|  | MFOL-1928 | Spreading | X |
|  | MFOL-1857 | Spreading | X |
|  | MFOL-328 | Moderately erect, close to BRRI dhan28 | Selected |
|  | MFOL-1956 | Erect, close to BRRI dhan28 | Selected |
|  | MFOL-111-328 | Spreading | X |
|  | MFOL-1856 | Spreading | X |
|  | MFOL-1772 | Spreading | X |
|  | MFOL-1779 | Spreading | X |
|  | MFOL-1838 | Spreading | X |
| ***OzT8* + *OzT9*** | MFOL-1491 | Erect, close to BRRI dhan28 | Selected |
|  | MFOL-2395 | Spreading | X |
|  | MFOL-2406 | Spreading | X |
|  | MFOL-1474 | Spreading | X |
|  | MFOL-2349 | Spreading | X |
|  | MFOL-1325 | Spreading | X |
|  | MFOL-1547 | Erect, close to BRRI dhan28 | Selected |
|  | MFOL-2388 | Moderately erect, close to BRRI dhan28 | Selected |
|  | MFOL-1378 | Moderately erect, close to BRRI dhan28 | Selected |
|  | MFOL-1488 | Moderately erect, close to BRRI dhan28 | Selected |
|  | MFOL-1720 | Spreading | X |
|  | MFOL-2301 | Spreading | X |

MFOL = Michael Frei Ozone-tolerant Lines. “Type” indicates plant architecture. Lines carrying QTLs *OzT8*, *OzT9*, or both were evaluated for plant type. Lines with an erect or moderately erect growth habit, closely resembling the recurrent parent BRRI dhan28 (marked as X), were selected for further breeding. Lines with a spreading growth habit were excluded, as this type lacks agronomic preference due to higher risk of lodging, shading effects, reduced planting density, and incompatibility with mechanized harvesting practices.

**Table S3** Morphological characteristics of breeding lines derived from the Binadhan-11 × Kasalath cross.

| **QTLs** | **Line** | **Type** | **Remarks** |
| --- | --- | --- | --- |
| ***OzT8*** | MFOL-1135 | Erect, similar to Binadhan-11 | Selected |
|  | MFOL-1198 | Erect, similar to Binadhan-11 | Selected |
|  | MFOL-1211 | Spreading | X |
|  | MFOL-1238 | Spreading | X |
|  | MFOL-1281 | Moderately erect, close to Binadhan-11 | Selected |
|  | MFOL-1231 | Erect, close to Binadhan-11 | Selected |
|  | MFOL-1163 | Moderately erect, close to Binadhan-11 | Selected |
|  | MFOL-1216 | Spreading | X |
|  | MFOL-1263 | Spreading | X |
|  | MFOL-1133 | Spreading | X |
|  | MFOL-1170 | Spreading | X |
|  | MFOL-1259 | Spreading | X |
|  | MFOL-1121 | Spreading | X |
|  | MFOL-1154 | Spreading | X |
|  | MFOL-1131 | Spreading | X |
| ***OzT9*** | MFOL-774 | Spreading | X |
|  | MFOL-1102 | Erect, close to Binadhan-11 | Selected |
|  | MFOL-1011 | Erect, close to Binadhan-11 | Selected |
|  | MFOL-1302 | Erect, close to Binadhan-11 | Selected |
|  | MFOL-1001 | Moderately erect, close to Binadhan-11 | Selected |
|  | MFOL-1031 | Moderately erect, close to Binadhan-11 | Selected |
|  | MFOL-866 | Spreading | X |
|  | MFOL-1289 | Spreading | X |
|  | MFOL-729 | Spreading | X |
|  | MFOL-842 | Spreading | X |
| ***OzT8 + OzT9*** | MFOL-471 | Moderately erect, close to Binadhan-11 | Selected |
|  | MFOL-544 | Moderately erect, close to Binadhan-11 | Selected |
|  | MFOL-579 | Moderately erect, close to Binadhan-11 | Selected |
|  | MFOL-491 | Spreading | X |
|  | MFOL-518 | Spreading | X |

MFOL = Michael Frei Ozone-tolerant Lines. “Type” indicates plant architecture. Lines carrying QTLs *OzT8*, *OzT9*, or both were evaluated for plant type. Lines with an erect or moderately erect growth habit, closely resembling the recurrent parent Binadhan-11 (marked as X), were selected for further breeding. Lines with a spreading growth habit were excluded, as this type lacks agronomic preference due to higher risk of lodging, shading effects, reduced planting density, and incompatibility with mechanized harvesting practices.

**Table S4** Descriptive statistics and analysis of variance (ANOVA) for physiological and biochemical traits of breeding lines derived from the cross between BRRI dhan28 and Kasalath under ozone stress and control conditions (screening experiment).

| **Traits** | **Date** | **LS means (treatment)** | | | | **ANOVA** | | | | | |
| --- | --- | --- | --- | --- | --- | --- | --- | --- | --- | --- | --- |
|  |  | **Control** | | **Ozone** | | **Ozone-Treatment** | | **Genotype** | | **Interaction** | |
|  |  | **Mean** | **SD** | **Mean** | **SD** | **Pr(>F)** | **F values** | **Pr(>F)** | **F values** | **Pr(>F)** | **F values** |
| LBS | 80 DAO | 0 | 0 | 3.46 | 0.79 | **0.0102** | 387.66 | **<0.0001** | 11.31 | **<0.0001** | 11.31 |
| NDVI | 80 DAO | 0.73 | 0.04 | 0.63 | 0.04 | **0.0311** | 418.74 | **<0.0001** | 4.13 | **<0.0001** | 4.40 |
| Lic2 | 80 DAO | 0.94 | 0.08 | 0.67 | 0.07 | **0.02545** | 625.01 | **0.0013** | 2.93 | 0.0615 | 1.76 |
| SC (mol m^−2^ s^−1^) | 80 DAO | 0.28 | 0.19 | 0.10 | 0.07 | 0.1453 | 18.52 | **0.0340** | 1.95 | 0.1638 | 1.42 |
| PhiPS2 | 80 DAO | 0.71 | 0.07 | 0.70 | 0.07 | 0.0605 | 110.17 | 0.1107 | 1.56 | 0.1926 | 1.36 |
| A (µmol m⁻² s⁻¹) | 116 DAO | 29.60 | 8.65 | 18.65 | 9.00 | 0.9612 | 0.00 | 0.9020 | 0.73 | 0.1736 | 1.21 |
| MDA (nmol g^−1^) FW | 50 DAO | 13.58 | 2.47 | 20.24 | 4.00 | **0.0354** | 321.82 | **<0.0001** | 8.61 | **<0.0001** | 6.60 |

Note: Mean values of all genotypes are shown. LS, least square means; SD, standard deviation; LBS, leaf bronzing score; NDVI, normalized difference vegetation index; Lic2, Lichtenthaler index 2;  SC, stomatal conductance(mol m^−2^ s^−1^); PhiPS2, quantum efficiency of photosystem II; A, CO_2_ assimilation rate (µmol m⁻² s⁻¹); MDA, malondialdehyde (nmol g^−1^); FW, fresh weight.

**Table S5** Descriptive statistics and analysis of variance (ANOVA) for physiological and biochemical traits of breeding lines derived from the cross between Binadhan-11 and Kasalath under ozone stress and control conditions (screening experiment).

| **Traits** | **Date** | **LS means (treatment)** | | | | **ANOVA** | | | | | |
| --- | --- | --- | --- | --- | --- | --- | --- | --- | --- | --- | --- |
|  |  | **Control** | | **Ozone** | | **Ozone-Treatment** | | **Genotype** | | **Interaction** | |
|  |  | **Mean** | **SD** | **Mean** | **SD** | **Pr(>F)** | **F values** | **Pr(>F)** | **F values** | **Pr(>F)** | **F values** |
| LBS | 80 DAO | 0 | 0 | 3.98 | 0.97 | **0.0111** | 327.43 | **<0.0001** | 9.51 | **<0.0001** | 9.51 |
| NDVI | 80 DAO | 0.74 | 0.02 | 0.62 | 0.06 | **0.0407** | 244.07 | **<0.0001** | 4.83 | **0.0002** | 3.28 |
| Lic2 | 80 DAO | 0.95 | 0.07 | 0.64 | 0.08 | **0.0218** | 852.78 | **0.0010** | 2.89 | **0.0040** | 2.53 |
| gsw (mol m^−2^ s^−1^) | 80 DAO | 0.21 | 0.14 | 0.16 | 0.12 | 0.4205 | 1.66 | 0.0799 | 1.65 | 0.2804 | 1.21 |
| PhiPS2 | 80 DAO | 0.71 | 0.07 | 0.71 | 0.06 | 0.3637 | 2.42 | 0.4884 | 0.99 | 0.4177 | 1.04 |
| A (µmol m⁻² s⁻¹) | 116 DAO | 30.18 | 8.28 | 17.59 | 7.21 | 0.1264 | 24.70 | **<0.0001** | 3.13 | 0.0938 | 1.37 |
| MDA (nmol g^−1^) FW | 50 DAO | 11.97 | 2.33 | 18.18 | 4.00 | **0.0324** | 383.93 | **<0.0001** | 12.72 | **<0.0001** | 8.79 |

Note: Mean values of all genotypes are shown. LS, least square means; SD, standard deviation; LBS, leaf bronzing score; NDVI, normalized difference vegetation index; Lic2, Lichtenthaler index 2;  gsw, stomatal conductance(mol m^−2^ s^−1^); PhiPS2, quantum efficiency of photosystem II; A, CO_2_ assimilation rate (µmol m⁻² s⁻¹); MDA, malondialdehyde (nmol g^−1^); FW, fresh weight.

**Table S6** Descriptive statistics and analysis of variance (ANOVA) for physiological traits of breeding lines derived from the cross between BRRI dhan28 and Kasalath under ozone stress and control conditions (second experiment).

| **Traits** | **LS means (treatment)** | | | | **ANOVA** | | | | | |
| --- | --- | --- | --- | --- | --- | --- | --- | --- | --- | --- |
|  | **Control** | | **Ozone** | | **Ozone-Treatment** | | **Genotype** | | **Interaction** | |
|  | **Mean** | **SD** | **Mean** | **SD** | **Pr(>F)** | **F values** | **Pr(>F)** | **F values** | **Pr(>F)** | **F values** |
| LBS | 0.00 | 0.00 | 2.06 | 1.64 | **<0.0000** | 2171 | **<0.0000** | 15.952 | **<0.0000** | 15.952 |
| NDVI | 0.63 | 0.06 | 0.60 | 0.08 | **<0.0000** | 152.967 | **0.0000** | 6.002 | **0.0000** | 5.194 |
| Lic2 | 0.79 | 0.11 | 0.74 | 0.12 | **<0.0000** | 145.213 | **0.0000** | 4.848 | 0.1759 | 1.380 |
| gsw (mol m^−2^ s^−1^) | 0.38 | 0.21 | 0.14 | 0.13 | **<0.0000** | 983.928 | **0.0000** | 8.039 | **<0.0000** | 5.751 |
| PhiPS2 | 0.60 | 0.13 | 0.57 | 0.14 | **0.0000** | 26.141 | **0.0001** | 3.414 | 0.0587 | 1.745 |
| A (µmol m⁻² s⁻¹) | 55.21 | 9.19 | 44.48 | 8.97 | **<0.0000** | 86.618 | **0.0000** | 4.659 | **0.0143** | 2.247 |

Note: Mean values of all genotypes are shown. LS, least square means; SD, standard deviation; LBS, leaf bronzing score; NDVI, normalized difference vegetation index; Lic2, Lichtenthaler index 2; gsw, stomatal conductance(mol m^−2^ s^−1^); PhiPS2, quantum efficiency of photosystem II; A, CO_2_ assimilation rate (µmol m⁻² s⁻¹).

**Table S7** Descriptive statistics and analysis of variance (ANOVA) for physiological traits of breeding lines derived from the cross between Binadhan-11 and Kasalath under ozone stress and control conditions (second experiment).

| **Traits** | **LS means (treatment)** | | | | **ANOVA** | | | | | |
| --- | --- | --- | --- | --- | --- | --- | --- | --- | --- | --- |
|  | **Control** | | **Ozone** | | **Ozone-Treatment** | | **Genotype** | | **Interaction** | |
|  | **Mean** | **SD** | **Mean** | **SD** | **Pr(>F)** | **F values** | **Pr(>F)** | **F values** | **Pr(>F)** | **F values** |
| LBS | 0.00 | 0.00 | 2.12 | 1.59 | **<0.0000** | 2171.903 | **<0.0000** | 26.623 | **<0.0000** | 26.623 |
| NDVI | 0.66 | 0.05 | 0.65 | 0.05 | **0.0000** | 45.344 | **<0.0000** | 39.255 | **0.0000** | 8.100 |
| Lic2 | 0.84 | 0.08 | 0.82 | 0.17 | **0.0010** | 10.878 | **<0.0000** | 11.529 | **0.0069** | 2.443 |
| gsw (mol m^−2^ s^−1^) | 0.32 | 0.19 | 0.14 | 0.10 | **<0.0000** | 605.412 | **<0.0000** | 17.096 | **<0.0000** | 10.076 |
| PhiPS2 | 0.62 | 0.12 | 0.61 | **0.12** | **0.0106** | 6.555 | **0.3759** | 1.078 | **0.0000** | 3.916 |
| A (µmol m⁻² s⁻¹) | 46.63 | 9.17 | 44.34 | 13.22 | 0.0971 | 2.786 | **0.0000** | 8.055 | **0.0002** | 3.615 |

Note: Mean values of all genotypes are shown. LS, least square means; SD, standard deviation; LBS, leaf bronzing score; NDVI, normalized difference vegetation index; Lic2, Lichtenthaler index 2; gsw, stomatal conductance(mol m^−2^ s^−1^); PhiPS2, quantum efficiency of photosystem II; A, CO_2_ assimilation rate (µmol m⁻² s⁻¹).

**Table S8** Descriptive statistics and analysis of variance (ANOVA) of agronomic traits of breeding lines originating from BRRI dhan28 X Kasalath under ozone stress and control conditions (second experiment).

| **Traits** | **LS means (treatment)** | | | | **ANOVA** | | | | | |
| --- | --- | --- | --- | --- | --- | --- | --- | --- | --- | --- |
|  | **Control** | | **Ozone** | | **Ozone-Treatment** | | **Genotype** | | **Interaction** | |
|  | **Mean** | **SD** | **Mean** | **SD** | **Pr(>F)** | **F values** | **Pr(>F)** | **F values** | **Pr(>F)** | **F values** |
| Days to flowering | 96.02 | 5.91 | 91.88 | 5.77 | **<0.0000** | 254.898 | **<0.0000** | 143.422 | **0.0000** | 5.061 |
| Days to maturity | 131.18 | 5.65 | 125.16 | 5.36 | **<0.0000** | 722.293 | **<0.0000** | 172.015 | **<0.0000** | 10.443 |
| Plant height (cm) | 132.11 | 15.70 | 127.10 | 15.88 | **0.0000** | 47.030 | **<0.0000** | 132.938 | 0.9886 | 0.284 |
| Panicle number | 9.94 | 2.62 | 9.76 | 2.26 | 0.4447 | 0.585 | **0.0000** | 4.436 | 0.3682 | 1.090 |
| Filled grain number | 1333.51 | 306.78 | 1208.93 | 314.14 | **0.0000** | 18.886 | **0.0000** | 7.930 | 0.1163 | 1.537 |
| Grain yield (g) | 24.42 | 5.31 | 21.70 | 5.35 | **0.0000** | 30.195 | **0.0000** | 7.408 | 0.0655 | 1.729 |
| Straw biomass (g) | 26.69 | 7.70 | 22.68 | 6.89 | **0.0000** | 47.907 | **<0.0000** | 23.598 | 0.939 | 0.438 |
| Harvest index | 48.19 | 4.89 | 49.14 | 4.24 | **0.0135** | 6.151 | **<0.0000** | 14.794 | **0.0024** | 2.687 |

Note: Mean values of all genotypes are shown. LS, least square means; SD, standard deviation.

**Table S9** Descriptive statistics and analysis of variance (ANOVA) of agronomic traits of breeding lines originating from Binadhan-11 X Kasalath under ozone stress and control conditions (second experiment).

| **Traits** | **LS means (treatment)** | | | | **ANOVA** | | | | | |
| --- | --- | --- | --- | --- | --- | --- | --- | --- | --- | --- |
|  | **Control** | | **Ozone** | | **Ozone-Treatment** | | **Genotype** | | **Interaction** | |
|  | **Mean** | **SD** | **Mean** | **SD** | **Pr(>F)** | **F values** | **Pr(>F)** | **F values** | **Pr(>F)** | **F values** |
| Days to flowering | 124.66 | 13.24 | 120.15 | 13.37 | **<0.0000** | 310.049 | **<0.0000** | 1035.004 | 0.0598 | 1.799 |
| Days to maturity | 158.05 | 13.09 | 152.14 | 13.17 | **<0.0000** | 768.968 | **<0.0000** | 1471.587 | **0.0003** | 3.388 |
| Plant height (cm) | 114.22 | 21.01 | 112.00 | 20.28 | **0.0012** | 10.617 | **<0.0000** | 330.433 | 0.5406 | 0.892 |
| Panicle number | 9.66 | 2.81 | 9.30 | 2.67 | 0.1751 | 1.846 | **0.0000** | 5.522 | **0.1319** | 1.517 |
| Filled grain number | 877.39 | 331.07 | 821.83 | 359.74 | **0.0114** | 6.472 | **<0.0000** | 63.901 | **0.0314** | 2.014 |
| Grain yield (g) | 19.24 | 6.18 | 17.42 | 6.76 | **0.0002** | 14.632 | **<0.0000** | 37.665 | **0.0107** | 2.354 |
| Straw biomass (g) | 36.30 | 9.62 | 32.71 | 8.95 | **0.0001** | 16.624 | **0.0000** | 9.352 | 0.3618 | 1.099 |
| Harvest index | 34.57 | 6.90 | 34.31 | 7.30 | 0.5714 | 0.321 | **<0.0000** | 57.306 | **0.0273** | 2.060 |

Note: Mean values of all genotypes are shown. LS, least square means; SD, standard deviation.

**Table S10.** Comparison of rice breeding lines derived from the cross between BRRI dhan28 and Kasalath under control and ozone treatments based on physiological and morphological traits using Tukey–Kramer groupings (second experiment).

| Genotype | Treatment | LBS | | NDVI | | Lic2 | | gsw (mol m^−2^ s^−1^) | | PhiPS2 | | A (µmol m⁻² s⁻¹) | |
| --- | --- | --- | --- | --- | --- | --- | --- | --- | --- | --- | --- | --- | --- |
|  |  | emmean | T-K groupings | emmean | T-K groupings | emmean | T-K groupings | emmean | T-K groupings | emmean | T-K groupings | emmean | T-K groupings |
| BRRI dhan28 | Control | 0.00 | d | 0.66 | a | 0.82 | a | 0.48 | a | 0.65 | a | 60.87 | ab |
| BRRI dhan28 | Ozone | 3.86 | a | 0.56 | f | 0.71 | gh | 0.06 | g | 0.54 | c | 36.06 | g |
| Kasalath | Control | 0.00 | d | 0.61 | bcde | 0.75 | bcdefg | 0.48 | a | 0.63 | ab | 56.40 | abcd |
| Kasalath | Ozone | 1.83 | c | 0.57 | f | 0.69 | h | 0.24 | de | 0.61 | abc | 48.50 | abcdefg |
| MFOL-1378 | Control | 0.00 | d | 0.63 | abcd | 0.79 | abcde | 0.36 | bc | 0.59 | abc | 44.42 | cdefg |
| MFOL-1378 | Ozone | 1.66 | c | 0.61 | bcde | 0.75 | cdefgh | 0.13 | fg | 0.58 | abc | 38.02 | fg |
| MFOL-1488 | Control | 0.00 | d | 0.64 | abc | 0.82 | a | 0.39 | ab | 0.60 | abc | 50.93 | abcdef |
| MFOL-1488 | Ozone | 1.78 | c | 0.63 | abcd | 0.77 | abcdef | 0.16 | ef | 0.58 | abc | 43.65 | defg |
| MFOL-1491 | Control | 0.00 | d | 0.63 | abcd | 0.78 | abcdef | 0.35 | bc | 0.59 | abc | 51.89 | abcdef |
| MFOL-1491 | Ozone | 1.88 | c | 0.60 | de | 0.74 | efgh | 0.14 | fg | 0.56 | c | 50.73 | abcdefg |
| MFOL-1547 | Control | 0.00 | d | 0.63 | abcd | 0.80 | abcde | 0.35 | bc | 0.58 | abc | 57.82 | abcd |
| MFOL-1547 | Ozone | 1.77 | c | 0.61 | bcde | 0.75 | bcdefg | 0.12 | fg | 0.56 | bc | 48.26 | abcdefg |
| MFOL-1956 | Control | 0.00 | d | 0.65 | ab | 0.81 | ab | 0.39 | ab | 0.61 | abc | 63.03 | a |
| MFOL-1956 | Ozone | 1.64 | c | 0.62 | bcde | 0.75 | bcdefg | 0.13 | fg | 0.59 | abc | 48.08 | bcdefg |
| MFOL-2197 | Control | 0.00 | d | 0.63 | abcd | 0.78 | abcdef | 0.40 | ab | 0.57 | bc | 58.67 | abc |
| MFOL-2197 | Ozone | 2.45 | b | 0.60 | de | 0.72 | fgh | 0.17 | ef | 0.54 | c | 45.39 | cdefg |
| MFOL-2236 | Control | 0.00 | d | 0.64 | abc | 0.78 | abcdef | 0.38 | b | 0.61 | abc | 58.09 | abcd |
| MFOL-2236 | Ozone | 2.17 | bc | 0.59 | ef | 0.73 | fgh | 0.15 | efg | 0.59 | abc | 52.44 | abcdef |
| MFOL-230 | Control | 0.00 | d | 0.63 | abcd | 0.80 | abc | 0.38 | b | 0.60 | abc | 50.13 | abcdefg |
| MFOL-230 | Ozone | 1.98 | bc | 0.59 | ef | 0.74 | defgh | 0.13 | fg | 0.59 | abc | 39.22 | efg |
| MFOL-233 | Control | 0.00 | d | 0.64 | abcd | 0.80 | abcd | 0.38 | b | 0.60 | abc | 53.64 | abcde |
| MFOL-233 | Ozone | 1.88 | c | 0.61 | cde | 0.74 | defgh | 0.16 | ef | 0.56 | bc | 40.05 | efg |
| MFOL-328 | Control | 0.00 | d | 0.63 | abcd | 0.78 | abcdef | 0.27 | cd | 0.57 | bc | 56.64 | abcd |
| MFOL-328 | Ozone | 1.80 | c | 0.60 | de | 0.74 | efgh | 0.12 | fg | 0.55 | c | 43.38 | defg |

Note: Data represent estimated marginal means (EMMeans) of measured traits under control and ozone treatments. Tukey–Kramer groupings (T-K groupings) indicate statistical differences among genotypes and treatments at p ≤ 0.05; values sharing the same letter are not significantly different. LBS, leaf bronzing score; NDVI, normalized difference vegetation index; Lic2, Lichtenthaler index 2; gsw, stomatal conductance(mol m^−2^ s^−1^); PhiPS2, quantum efficiency of photosystem II; A, CO_2_ assimilation rate (µmol m⁻² s⁻¹).

**Table S11.** Comparison of rice breeding lines derived from the cross between Binadhan-11 and Kasalath under control and ozone treatments based on physiological and morphological traits using Tukey–Kramer groupings (second experiment).

| Genotype | Treatment | LBS | | NDVI | | Lic2 | | gsw (mol m^−2^ s^−1^) | | PhiPS2 | | A (µmol m⁻² s⁻¹) | |
| --- | --- | --- | --- | --- | --- | --- | --- | --- | --- | --- | --- | --- | --- |
|  |  | emmean | T-K groupings | emmean | T-K groupings | emmean | T-K groupings | emmean | T-K groupings | emmean | T-K groupings | emmean | T-K groupings |
| Binadhan-11 | Control | 0.00 | e | 0.69 | a | 0.88 | a | 0.41 | ab | 0.68 | a | 45.73 | abc |
| Binadhan-11 | Ozone | 4.03 | a | 0.62 | d | 0.79 | bc | 0.07 | j | 0.57 | c | 21.62 | d |
| Kasalath | Control | 0.00 | e | 0.61 | d | 0.75 | cd | 0.48 | a | 0.63 | abc | 56.40 | a |
| Kasalath | Ozone | 1.83 | cd | 0.57 | e | 0.69 | d | 0.24 | def | 0.61 | bc | 48.50 | abc |
| MFOL-1011 | Control | 0.00 | e | 0.69 | ab | 0.87 | a | 0.31 | cde | 0.62 | abc | 39.40 | bc |
| MFOL-1011 | Ozone | 2.02 | c | 0.67 | abc | 0.84 | ab | 0.13 | ij | 0.62 | abc | 33.47 | cd |
| MFOL-1102 | Ozone | 0.00 | e | 0.67 | abc | 0.85 | ab | 0.29 | cde | 0.62 | abc | 49.94 | abc |
| MFOL-1102 | Control | 0.00 | cd | 0.67 | abc | 0.84 | ab | 0.15 | ghi | 0.61 | bc | 48.78 | abc |
| MFOL-1135 | Control | 0.00 | e | 0.67 | abc | 0.86 | ab | 0.28 | cde | 0.62 | abc | 54.79 | ab |
| MFOL-1135 | Ozone | 1.94 | c | 0.66 | abc | 0.83 | abc | 0.11 | ij | 0.60 | bc | 44.21 | abc |
| MFOL-1198 | Control | 0.00 | e | 0.67 | abc | 0.84 | ab | 0.30 | cde | 0.61 | bc | 48.92 | abc |
| MFOL-1198 | Ozone | 1.89 | cd | 0.65 | c | 0.83 | abc | 0.12 | ij | 0.60 | bc | 46.60 | abc |
| MFOL-1302 | Control | 0.00 | e | 0.66 | bc | 0.83 | abc | 0.35 | bc | 0.61 | bc | 46.94 | abc |
| MFOL-1302 | Ozone | 1.92 | c | 0.66 | c | 0.82 | abc | 0.15 | hij | 0.60 | bc | 45.12 | abc |
| MFOL-471 | Ozone | 0.00 | e | 0.66 | abc | 0.83 | abc | 0.23 | efg | 0.61 | bc | 49.12 | abc |
| MFOL-471 | Control | 0.00 | b | 0.66 | c | 0.81 | abc | 0.17 | fghi | 0.60 | bc | 47.86 | abc |
| MFOL-50 | Control | 0.00 | e | 0.67 | abc | 0.88 | a | 0.27 | cde | 0.63 | abc | 42.95 | abc |
| MFOL-50 | Ozone | 1.36 | d | 0.66 | bc | 0.84 | ab | 0.15 | ghi | 0.61 | bc | 40.40 | abc |
| MFOL-544 | Control | 0.00 | e | 0.67 | abc | 0.84 | ab | 0.22 | efgh | 0.61 | bc | 41.48 | abc |
| MFOL-544 | Ozone | 1.92 | c | 0.66 | abc | 0.84 | ab | 0.11 | ij | 0.59 | bc | 40.75 | abc |
| MFOL-60 | Control | 0.00 | e | 0.66 | bc | 0.84 | ab | 0.32 | cd | 0.64 | ab | 53.97 | ab |
| MFOL-60 | Ozone | 1.64 | cd | 0.66 | c | 0.82 | abc | 0.14 | hij | 0.63 | abc | 53.67 | ab |

Note: Data represent estimated marginal means (EMMeans) of measured traits under control and ozone treatments. Tukey–Kramer groupings (T-K groupings) indicate statistical differences among genotypes and treatments at p ≤ 0.05; values sharing the same letter are not significantly different. LBS, leaf bronzing score; NDVI, normalized difference vegetation index; Lic2, Lichtenthaler index 2; gsw, stomatal conductance(mol m^−2^ s^−1^); PhiPS2, quantum efficiency of photosystem II; A, CO_2_ assimilation rate (µmol m⁻² s⁻¹).

**Table S12.** Comparison of rice breeding lines derived from the cross between BRRI dhan28 and Kasalath under control and ozone treatments based on agronomic traits using Tukey–Kramer groupings (second experiment).

| Genotype | Treatment | DTF | | DTM | | PH | | PN | | FGN | | GY | | SB | | HI | |
| --- | --- | --- | --- | --- | --- | --- | --- | --- | --- | --- | --- | --- | --- | --- | --- | --- | --- |
|  |  | emmean | T-K groupings | emmean | T-K groupings | emmean | T-K groupings | emmean | T-K groupings | emmean | T-K groupings | emmean | T-K groupings | emmean | T-K groupings | emmean | T-K groupings |
| BRRI dhan28 | Control | 96 | cdefg | 133 | bcd | 130 | cdef | 10.38 | abc | 1338 | abcde | 27.05 | ab | 27.49 | cd | 49.59 | abc |
| BRRI dhan28 | Ozone | 89 | k | 122 | k | 126 | defgh | 8.25 | bc | 987 | e | 18.98 | ef | 20.39 | defg | 48.15 | bcd |
| Kasalath | Control | 97 | cdef | 129 | fg | 174 | a | 11.50 | a | 1600 | a | 28.42 | a | 40.13 | a | 43.40 | de |
| Kasalath | Ozone | 92 | jk | 122 | jk | 168 | a | 10.88 | ab | 1516 | ab | 27.18 | ab | 35.80 | ab | 41.34 | e |
| MFOL-1378 | Control | 81 | l | 116 | l | 113 | ij | 10.19 | abc | 1565 | a | 22.51 | abcdef | 19.66 | fg | 53.51 | a |
| MFOL-1378 | Ozone | 79 | l | 114 | l | 107 | j | 9.69 | abc | 1326 | abcde | 18.65 | ef | 17.53 | g | 51.15 | ab |
| MFOL-1488 | Control | 96 | efghi | 132 | cdef | 125 | efgh | 11.50 | a | 1251 | abcde | 24.77 | abcde | 26.22 | cdef | 49.90 | abc |
| MFOL-1488 | Ozone | 92 | jk | 126 | hi | 120 | ghi | 10.38 | abc | 1154 | bcde | 21.90 | bcdef | 21.74 | defg | 48.96 | abc |
| MFOL-1491 | Control | 97 | bcde | 131 | def | 134 | bcd | 9.75 | abc | 1310 | abcde | 25.51 | abc | 27.06 | cde | 52.01 | ab |
| MFOL-1491 | Ozone | 93 | ghij | 126 | h | 128 | cdefg | 9.50 | abc | 1304 | abcde | 24.70 | abcde | 22.92 | cdefg | 48.95 | abc |
| MFOL-1547 | Control | 99 | abcd | 130 | ef | 122 | fghi | 8.19 | bc | 1150 | cde | 19.19 | def | 22.77 | cdefg | 49.67 | abc |
| MFOL-1547 | Ozone | 93 | hij | 125 | hij | 118 | hi | 7.69 | c | 1145 | cde | 18.19 | f | 18.43 | g | 45.94 | cde |
| MFOL-1956 | Control | 100 | ab | 135 | ab | 127 | cdefg | 10.38 | abc | 1195 | bcde | 23.20 | abcdef | 24.59 | cdefg | 49.38 | abc |
| MFOL-1956 | Ozone | 100 | abc | 133 | bcde | 124 | efgh | 9.81 | abc | 1018 | de | 20.07 | cdef | 20.48 | defg | 48.71 | abc |
| MFOL-2197 | Control | 99 | abcd | 133 | bcd | 132 | bcde | 10.50 | abc | 1369 | abcd | 23.07 | abcdef | 26.82 | cdef | 48.01 | bcd |
| MFOL-2197 | Ozone | 93 | ij | 126 | h | 127 | defgh | 10.31 | abc | 1273 | abcde | 21.36 | bcdef | 23.01 | cdefg | 46.19 | cde |
| MFOL-2236 | Control | 101 | a | 136 | a | 140 | b | 10.13 | abc | 1427 | abc | 24.63 | abcde | 29.77 | bc | 47.50 | bcd |
| MFOL-2236 | Ozone | 96 | defgh | 130 | ef | 136 | bc | 9.81 | abc | 1378 | abcd | 24.23 | abcdef | 27.36 | cde | 45.70 | cde |
| MFOL-230 | Control | 100 | ab | 137 | a | 128 | cdefg | 9.31 | abc | 1397 | abc | 25.46 | abcd | 23.25 | cdefg | 52.04 | ab |
| MFOL-230 | Ozone | 98 | bcde | 130 | ef | 124 | efgh | 8.81 | abc | 1091 | cde | 19.92 | cdef | 20.08 | efg | 50.01 | abc |
| MFOL-233 | Control | 94 | fghij | 134 | abc | 130 | bcdef | 10.63 | abc | 1178 | bcde | 23.96 | abcdef | 24.86 | cdefg | 49.44 | abc |
| MFOL-233 | Ozone | 91 | jk | 126 | h | 122 | fghi | 9.94 | abc | 1083 | cde | 21.10 | bcdef | 21.92 | defg | 48.69 | abc |
| MFOL-328 | Control | 91 | jk | 127 | gh | 131 | bcdef | 9.88 | abc | 1278 | abcde | 25.28 | abcd | 27.70 | cd | 51.77 | ab |
| MFOL-328 | Ozone | 89 | k | 123 | ijk | 125 | efgh | 9.06 | abc | 1177 | bcde | 24.16 | abcdef | 22.48 | cdefg | 47.88 | bcd |

Note: Data represent estimated marginal means (EMMeans) of measured traits under control and ozone treatments. Tukey–Kramer groupings (T-K groupings) indicate statistical differences among genotypes and treatments at p ≤ 0.05; values sharing the same letter are not significantly different. DTF, days to flowering; DTM, days to maturity; PH, plant height; PN, panicle number; FGN, filled grain number; GY, grain yield; SB, straw biomass; HI, harvest index.

**Table S13.** Comparison of rice breeding lines derived from the cross between Binadhan-11 and Kasalath under control and ozone treatments based on agronomic traits using Tukey–Kramer groupings (second experiment).

| Genotype | Treatment | DTF | | DTM | | PH | | PN | | FGN | | GY | | SB | | HI | |
| --- | --- | --- | --- | --- | --- | --- | --- | --- | --- | --- | --- | --- | --- | --- | --- | --- | --- |
|  |  | emmean | T-K groupings | emmean | T-K groupings | emmean | T-K groupings | emmean | T-K groupings | emmean | T-K groupings | emmean | T-K groupings | emmean | T-K groupings | emmean | T-K groupings |
| Binadhan-11 | Control | 136 | a | 170 | a | 108 | def | 11.50 | a | 822 | bcde | 20.07 | bcd | 39.31 | abcde | 34.11 | cdefg |
| Binadhan-11 | Ozone | 129 | efgh | 161 | ef | 107 | def | 11.06 | ab | 500 | fg | 11.77 | ij | 29.72 | def | 28.73 | fghij |
| Kasalath | Control | 97 | l | 129 | j | 174 | a | 10.88 | abc | 1600 | a | 28.42 | a | 40.13 | abcd | 43.40 | a |
| Kasalath | Ozone | 92 | m | 122 | k | 168 | a | 10.56 | abc | 1516 | a | 27.18 | a | 35.80 | abcdef | 41.34 | ab |
| MFOL-1011 | Control | 132 | bcd | 163 | d | 95 | gh | 10.38 | abc | 695 | defg | 13.66 | efghij | 33.99 | bcdef | 28.55 | ghij |
| MFOL-1011 | Ozone | 128 | efgh | 158 | fg | 90 | h | 10.06 | abc | 659 | defg | 12.73 | ghij | 32.86 | bcdef | 28.25 | hij |
| MFOL-1102 | Ozone | 129 | efgh | 164 | d | 102 | fg | 10.00 | abcd | 739 | cdef | 19.58 | bcd | 40.24 | abcd | 32.95 | defgh |
| MFOL-1102 | Control | 126 | hi | 158 | g | 102 | fg | 10.00 | abcd | 707 | defg | 17.97 | cdefgh | 35.74 | abcdef | 32.58 | defgh |
| MFOL-1135 | Control | 131 | cdef | 164 | cd | 125 | b | 9.94 | abcd | 1081 | b | 24.57 | ab | 44.89 | a | 36.37 | bcde |
| MFOL-1135 | Ozone | 127 | gh | 159 | fg | 121 | bc | 9.81 | abcd | 982 | bc | 23.24 | abc | 43.00 | ab | 34.31 | cdef |
| MFOL-1198 | Control | 133 | abc | 166 | bc | 106 | def | 9.81 | abcd | 913 | bcd | 19.33 | bcde | 29.73 | def | 40.04 | ab |
| MFOL-1198 | Ozone | 129 | defg | 160 | ef | 105 | def | 9.69 | abcd | 904 | bcd | 18.18 | cdefg | 28.17 | f | 39.42 | abc |
| MFOL-1302 | Control | 134 | ab | 168 | ab | 106 | def | 9.44 | abcd | 754 | cdef | 17.90 | cdefgh | 40.33 | abc | 32.98 | defgh |
| MFOL-1302 | Ozone | 129 | efgh | 162 | de | 105 | def | 9.38 | abcd | 712 | defg | 16.12 | defghi | 32.88 | bcdef | 30.89 | efghi |
| MFOL-471 | Ozone | 128 | fgh | 162 | de | 111 | de | 9.25 | abcd | 863 | bcde | 18.72 | cdef | 30.37 | cdef | 40.10 | ab |
| MFOL-471 | Control | 124 | i | 157 | g | 109 | def | 9.25 | abcd | 843 | bcde | 18.44 | cdefg | 29.08 | ef | 38.10 | abcd |
| MFOL-50 | Control | 109 | j | 142 | h | 113 | cd | 8.94 | abcd | 685 | defg | 15.23 | defghij | 34.50 | abcdef | 30.38 | fghi |
| MFOL-50 | Ozone | 104 | k | 137 | i | 111 | de | 8.56 | abcd | 608 | efg | 13.39 | fghij | 32.59 | bcdef | 29.66 | fghij |
| MFOL-544 | Control | 135 | a | 168 | ab | 106 | def | 7.88 | bcd | 541 | fg | 12.41 | hij | 35.01 | abcdef | 25.85 | ij |
| MFOL-544 | Ozone | 131 | cde | 163 | de | 104 | ef | 7.75 | cd | 460 | g | 10.20 | j | 31.52 | cdef | 24.43 | j |
| MFOL-60 | Control | 107 | j | 142 | h | 112 | d | 7.69 | cd | 1078 | b | 23.30 | abc | 32.04 | cdef | 43.10 | a |
| MFOL-60 | Ozone | 103 | k | 137 | i | 109 | def | 6.75 | d | 1029 | b | 20.73 | bcd | 27.12 | f | 42.16 | a |

Note: Data represent estimated marginal means (EMMeans) of measured traits under control and ozone treatments. Tukey–Kramer groupings (T-K groupings) indicate statistical differences among genotypes and treatments at p ≤ 0.05; values sharing the same letter are not significantly different. DTF, days to flowering; DTM, days to maturity; PH, plant height; PN, panicle number; FGN, filled grain number; GY, grain yield; SB, straw biomass; HI, harvest index.

**Table S14** Principal component (PC) scores and ranks of rice cultivars along the first two principal components (PC1 and PC2). “PC1 score” and “PC2 score” represent each cultivar’s coordinate on the respective axis. “Rank PC1” and “Rank PC2” indicate the relative position of cultivars when ordered by their scores, with higher scores corresponding to higher ranks. These ranks provide a comparative distribution of cultivar sensitivity or resistance to ozone as represented in the PCA biplot (Figure 5).

| Genotype | PC1_score | Rank_PC1 | PC2_score | Rank_PC2 |
| --- | --- | --- | --- | --- |
| BRRI dhan28 x Kaslaath | | | | |
| MFOL-1491 | 1.86 | 1 | 1.27 | 4 |
| MFOL-328 | 1.72 | 2 | 0.48 | 7 |
| MFOL-2236 | 1.54 | 3 | 1.58 | 1 |
| MFOL-1547 | 1.41 | 4 | 1.51 | 2 |
| MFOL-1378 | 1.41 | 5 | -2.67 | 12 |
| Kasalath | 0.65 | 6 | 1.37 | 3 |
| MFOL-1488 | 0.58 | 7 | -0.19 | 8 |
| MFOL-1956 | 0.47 | 8 | -2.16 | 10 |
| MFOL-233 | 0.29 | 9 | -0.66 | 9 |
| MFOL-2197 | 0.01 | 10 | 1.19 | 5 |
| MFOL-230 | -1.05 | 11 | -2.39 | 11 |
| BRRI dhan28 | -8.89 | 12 | 0.68 | 6 |
| Binadhan11 x Kasalath | | | | |
| MFOL-1135 | 2.58 | 1 | -1.03 | 10 |
| MFOL-471 | 2.22 | 2 | 1.47 | 2 |
| MFOL-1102 | 1.32 | 3 | 0.45 | 5 |
| MFOL-1198 | 1.21 | 4 | 0.05 | 7 |
| MFOL-1011 | 0.94 | 5 | 0.36 | 6 |
| MFOL-50 | 0.65 | 6 | 0.70 | 4 |
| MFOL-60 | 0.56 | 7 | -0.57 | 8 |
| MFOL-1302 | 0.27 | 8 | -0.68 | 9 |
| MFOL-544 | 0.26 | 9 | 1.55 | 1 |
| Kasalath | -1.58 | 10 | -3.11 | 11 |
| Binadhan-11 | -8.42 | 11 | 0.81 | 3 |

**Table S15** Loadings and contributions of phenotypic traits to the first two principal components (PC1 and PC2) from PCA. PC1 and PC2 loadings indicate the correlation of each trait with the respective axis. “PC1 contribution (%)” and “PC2 contribution (%)” give the proportion of each trait’s variance accounted for in constructing that axis. “Total contribution (PC1+PC2, %)” indicates the overall influence of each trait on the PCA biplot (Figure 5).

| Trait | PC1_loading | PC2_loading | PC1 contribution (%) | PC2 contribution (%) | Total contribution (PC1+PC2, %) |
| --- | --- | --- | --- | --- | --- |
| BRRI dhan28 x Kaslaath | | | | | |
| Lic2 | 0.94 | -0.02 | 11.36 | 0.02 | 11.39 |
| LBS | -0.92 | 0.29 | 10.74 | 3.59 | 14.34 |
| PhiPS2 | 0.85 | -0.32 | 9.30 | 4.20 | 13.50 |
| NDVI | 0.85 | -0.27 | 9.23 | 2.99 | 12.22 |
| gsw | 0.83 | 0.18 | 8.87 | 1.38 | 10.24 |
| GY | 0.81 | 0.57 | 8.31 | 13.67 | 21.99 |
| A | 0.80 | 0.19 | 8.09 | 1.59 | 9.68 |
| PN | 0.77 | 0.03 | 7.52 | 0.04 | 7.56 |
| DTM | 0.77 | -0.46 | 7.48 | 9.09 | 16.58 |
| FGN | 0.73 | 0.64 | 6.78 | 17.09 | 23.87 |
| SB | 0.65 | -0.13 | 5.46 | 0.67 | 6.13 |
| DTF | 0.54 | -0.73 | 3.73 | 22.57 | 26.30 |
| HI | 0.50 | 0.74 | 3.13 | 23.10 | 26.23 |
| Binadhan11 x Kasalath | | | | | |
| FGN | 0.92 | -0.31 | 10.30 | 5.89 | 16.19 |
| A | 0.91 | -0.12 | 10.19 | 0.90 | 11.09 |
| PhiPS2 | 0.91 | -0.04 | 10.03 | 0.10 | 10.12 |
| GY | 0.89 | -0.33 | 9.79 | 6.79 | 16.58 |
| PN | 0.87 | -0.12 | 9.25 | 0.93 | 10.18 |
| NDVI | 0.86 | 0.39 | 9.08 | 9.73 | 18.81 |
| DTM | 0.78 | 0.50 | 7.43 | 15.50 | 22.94 |
| SB | 0.74 | 0.19 | 6.75 | 2.26 | 9.01 |
| gsw | 0.70 | 0.15 | 5.98 | 1.41 | 7.39 |
| LBS | -0.67 | 0.34 | 5.55 | 7.10 | 12.65 |
| DTF | 0.66 | 0.40 | 5.36 | 9.83 | 15.19 |
| HI | 0.66 | -0.68 | 5.35 | 28.67 | 34.02 |
| Lic2 | 0.64 | 0.42 | 4.96 | 10.89 | 15.84 |

*Trait abbreviations: LBS, leaf bronzing score; NDVI, normalized difference vegetation index; Lic2, Lichtenthaler index 2; gsw, stomatal conductance; PhiPS2, quantum efficiency of photosystem II; A, CO₂ assimilation rate; DTF, days to flowering; DTM, days to maturity; PH, plant height; PN, panicle number; FGN, filled grain number; GY, grain yield; SB, straw biomass; HI, harvest index.*

**Table S16** Descriptive statistics and ANOVA of grain yield, straw biomass, and harvest index of breeding lines derived from BRRI dhan28 × Kasalath under EDU and ambient ozone conditions (field experiment).

| **Traits** | **LS means (treatment)** | | | | **ANOVA** | | | | | |
| --- | --- | --- | --- | --- | --- | --- | --- | --- | --- | --- |
|  | **EDU** | | **Ambient ozone** | | **Ambient ozone-Treatment** | | **Genotype** | | **Interaction** | |
|  | **Mean** | **SD** | **Mean** | **SD** | **Pr(>F)** | **F values** | **Pr(>F)** | **F values** | **Pr(>F)** | **F values** |
| Grain yield (g) | 6.50 | 0.64 | 6.51 | 0.49 | 0.8827 | 0.022 | **0.0000** | 22.905 | **0.0001** | 7.121 |
| Straw biomass (g) | 6.76 | 0.78 | 6.69 | 0.81 | 0.4867 | 0.498 | **0.0000** | 42.606 | 0.1371 | 1.806 |
| Harvest index | 49.05 | 2.82 | 49.40 | 2.87 | 0.4595 | 0.564 | **0.0000** | 17.215 | 0.2045 | 1.541 |

Note: Mean values of all genotypes are shown. LS, least square means; SD, standard deviation.

**Table S17** Descriptive statistics and ANOVA of grain yield, straw biomass, and harvest index of breeding lines derived from Binadhan-11 × Kasalath under EDU and ambient ozone conditions (field experiment).

| **Traits** | **LS means (treatment)** | | | | **ANOVA** | | | | | |
| --- | --- | --- | --- | --- | --- | --- | --- | --- | --- | --- |
|  | **EDU** | | **Ambient ozone** | | **Ambient ozone-Treatment** | | **Genotype** | | **Interaction** | |
|  | **Mean** | **SD** | **Mean** | **SD** | **Pr(>F)** | **F values** | **Pr(>F)** | **F values** | **Pr(>F)** | **F values** |
| Grain yield (g) | 7.15 | 0.67 | 7.06 | 0.78 | 0.3448 | 0.926 | **0.0000** | 21.435 | **0.0000** | 9.322 |
| Straw biomass (g) | 6.66 | 0.66 | 6.60 | 0.54 | 0.5406 | 0.385 | **0.0000** | 13.758 | **0.0470** | 2.516 |
| Harvest index | 51.76 | 3.48 | 51.61 | 3.12 | 0.7770 | 0.082 | **0.0000** | 18.403 | 0.0956 | 2.044 |

Note: Mean values of all genotypes are shown. LS, least square means; SD, standard deviation.

**Table S18.** Comparison of rice breeding lines derived from the cross between BRRI dhan28 and Kasalath under EDU and ambient ozone conditions based on grain yield, straw biomass, and harvest index using Tukey–Kramer groupings (field experiment).

| Genotype | Treatment | GY | | SB | | HI | |
| --- | --- | --- | --- | --- | --- | --- | --- |
|  |  | emmean | T-K groupings | emmean | T-K groupings | emmean | T-K groupings |
| BRRI dhan28 | EDU | 6.99 | abcd | 6.23 | cd | 52.91 | a |
| BRRI dhan28 | Ambient-Ozone | 6.16 | e | 5.55 | d | 52.60 | ab |
| Kasalath | EDU | 6.05 | e | 7.48 | ab | 44.75 | f |
| Kasalath | Ambient-Ozone | 6.30 | de | 7.43 | ab | 45.89 | ef |
| MFOL-1491 | EDU | 6.44 | cde | 6.37 | cd | 50.30 | abcde |
| MFOL-1491 | Ambient-Ozone | 7.09 | abc | 6.44 | c | 52.42 | abc |
| MFOL-1547 | EDU | 5.92 | e | 6.02 | cd | 49.58 | abcde |
| MFOL-1547 | Ambient-Ozone | 6.41 | cde | 5.97 | cd | 51.78 | abcd |
| MFOL-1956 | EDU | 5.88 | e | 6.44 | c | 47.73 | def |
| MFOL-1956 | Ambient-Ozone | 6.14 | e | 6.82 | bc | 47.35 | def |
| MFOL-2236 | EDU | 6.60 | bcde | 6.62 | bc | 49.90 | abcde |
| MFOL-2236 | Ambient-Ozone | 6.16 | e | 6.68 | bc | 47.97 | cdef |
| MFOL-328 | EDU | 7.58 | a | 8.15 | a | 48.20 | bcdef |
| MFOL-328 | Ambient-Ozone | 7.29 | ab | 7.96 | a | 47.79 | def |

Note: Data represent estimated marginal means (EMMeans) of measured traits under control and ozone treatments. Tukey–Kramer groupings (T-K groupings) indicate statistical differences among genotypes and treatments at p ≤ 0.05; values sharing the same letter are not significantly different. GY, grain yield; SB, straw biomass; HI, harvest index.

**Table S19.** Comparison of rice breeding lines derived from the cross between Binadhan-11 and Kasalath under EDU and ambient ozone conditions based on grain yield, straw biomass, and harvest index using Tukey–Kramer groupings (field experiment).

| Genotype | Treatment | GY | | SB | | HI | |
| --- | --- | --- | --- | --- | --- | --- | --- |
|  |  | emmean | T-K groupings | emmean | T-K groupings | emmean | T-K groupings |
| Binadhan-11 | EDU | 7.53 | abc | 6.24 | bc | 54.67 | a |
| Binadhan-11 | Ambient-Ozone | 6.40 | def | 6.20 | bc | 50.75 | ab |
| Kasalath | EDU | 6.05 | f | 7.48 | a | 44.75 | c |
| Kasalath | Ambient-Ozone | 6.30 | ef | 7.43 | a | 45.89 | bc |
| MFOL-1102 | EDU | 8.26 | a | 7.54 | a | 52.32 | a |
| MFOL-1102 | Ambient-Ozone | 7.50 | abc | 6.60 | abc | 53.13 | a |
| MFOL-1135 | EDU | 6.88 | cdef | 6.35 | bc | 52.07 | a |
| MFOL-1135 | Ambient-Ozone | 6.26 | ef | 6.16 | bc | 50.46 | ab |
| MFOL-1302 | EDU | 6.99 | cde | 6.33 | bc | 52.48 | a |
| MFOL-1302 | Ambient-Ozone | 7.42 | abc | 6.60 | abc | 52.86 | a |
| MFOL-471 | EDU | 7.28 | bcd | 6.05 | c | 54.60 | a |
| MFOL-471 | Ambient-Ozone | 7.39 | abc | 6.10 | bc | 54.79 | a |
| MFOL-60 | EDU | 7.05 | cde | 6.65 | abc | 51.45 | a |
| MFOL-60 | Ambient-Ozone | 8.11 | ab | 7.08 | ab | 53.40 | a |

Note: Data represent estimated marginal means (EMMeans) of measured traits under control and ozone treatments. Tukey–Kramer groupings (T-K groupings) indicate statistical differences among genotypes and treatments at p ≤ 0.05; values sharing the same letter are not significantly different. GY, grain yield; SB, straw biomass; HI, harvest index.

**Table S20.** Weekly schedule of EDU applications (January–June 2024)

| Date | EDU Application Number |
| --- | --- |
| 1/28/2024 | EDU 1 |
| 2/4/2024 | EDU 2 |
| 2/11/2024 | EDU 3 |
| 2/18/2024 | EDU 4 |
| 2/25/2024 | EDU 5 |
| 3/3/2024 | EDU 6 |
| 3/10/2024 | EDU 7 |
| 3/17/2024 | EDU 8 |
| 3/24/2024 | EDU 9 |
| 3/31/2024 | EDU 10 |
| 4/7/2024 | EDU 11 |
| 4/14/2024 | EDU 12 |
| 4/21/2024 | EDU 13 |
| 4/28/2024 | EDU 14 |
| 5/5/2024 | EDU 15 |
| 5/12/2024 | EDU 16 |
| 5/19/2024 | EDU 17 |
| 5/26/2024 | EDU 18 |
| 6/2/2024 | EDU 19 |

**Table S21.** *Effects of ozone exposure on grain yield of rice (Oryza sativa L.) genotypes under greenhouse conditions.*
Average grain yield (g plant⁻¹) under control and ozone treatments in the second greenhouse experiment. Percent change represents the relative reduction (–) or increase (+) in yield under ozone compared with control.

| **Genotype** | **Average Yield from Control (g)** | **Average Yield from Ozone (g)** | **% Change (Reduction / Increase)** |
| --- | --- | --- | --- |
| **BRRI dhan28 x Kaslaath** | | | |
| BRRI dhan28 | 27.05 | 18.98 | -29.81 |
| Kasalath | 28.42 | 27.18 | -4.36 |
| MFOL-1378 | 22.51 | 18.65 | -17.14 |
| MFOL-1488 | 24.77 | 21.90 | -11.58 |
| MFOL-1491 | 25.51 | 24.70 | -3.16 |
| MFOL-1547 | 19.19 | 18.19 | -5.17 |
| MFOL-1956 | 23.20 | 20.07 | -13.50 |
| MFOL-2197 | 23.07 | 21.36 | -7.41 |
| MFOL-2236 | 24.63 | 24.23 | -1.66 |
| MFOL-230 | 25.46 | 19.92 | -21.79 |
| MFOL-233 | 23.96 | 21.10 | -11.94 |
| MFOL-328 | 25.28 | 24.16 | -4.44 |
| **Binadhan11 x Kasalath** | | | |
| Binadhan-11 | 20.07 | 11.77 | -41.34 |
| MFOL-1011 | 13.66 | 12.73 | -6.81 |
| MFOL-1102 | 19.58 | 17.97 | -8.22 |
| MFOL-1135 | 23.24 | 24.57 | 5.71 |
| MFOL-1198 | 19.33 | 18.18 | -5.95 |
| MFOL-1302 | 17.90 | 16.12 | -9.93 |
| MFOL-471 | 18.44 | 18.72 | 1.51 |
| MFOL-50 | 15.23 | 13.39 | -12.06 |
| MFOL-544 | 12.41 | 10.20 | -17.83 |
| MFOL-60 | 23.30 | 20.73 | -11.00 |

**Table S22.** *Effects of ozone exposure on grain yield of rice (Oryza sativa L.) genotypes under field conditions.*
Average grain yield (g plant⁻¹) under control and ozone treatments in the field experiment. Percent change represents the relative reduction (–) or increase (+) in yield under ozone compared with control.

| **Genotype** | **Average Yield from Control (g)** | **Average Yield from Ozone (g)** | **% Change (Reduction / Increase)** |
| --- | --- | --- | --- |
| **BRRI dhan28 x Kaslaath** | | | |
| BRRI dhan28 | 6.99 | 6.16 | -11.87 |
| Kasalath | 6.05 | 6.30 | 4.07 |
| MFOL-1491 | 6.44 | 7.09 | 10.09 |
| MFOL-1547 | 5.92 | 6.41 | 8.33 |
| MFOL-1956 | 5.88 | 6.14 | 4.31 |
| MFOL-2236 | 6.60 | 6.16 | -6.72 |
| MFOL-328 | 7.58 | 7.29 | -3.83 |
| **Binadhan11 x Kasalath** | | | |
| Binadhan-11 | 7.53 | 6.40 | -15.01 |
| MFOL-1102 | 8.26 | 7.50 | -9.16 |
| MFOL-1135 | 6.88 | 6.26 | -8.96 |
| MFOL-1302 | 6.99 | 7.42 | 6.25 |
| MFOL-471 | 7.28 | 7.39 | 1.51 |
| MFOL-60 | 7.05 | 8.11 | 15.14 |

**Supplementary Protocol S1**

Marker-assisted backcross breeding (MABC) to introgress ozone-tolerance QTLs (*OzT8*, *OzT9*) into Indica rice

**1. Overview**

This protocol documents a practical and reproducible pipeline for transferring ozone-tolerance QTLs *OzT8* and *OzT9* from the donor Kasalath into the recurrent parents BRRI dhan28 and Binadhan‑11 using KASP genotyping.

**2. Glossary (breeding-specific)**

**Polymorphism:** The presence of genetic variation at a locus among individuals or populations, forming the basis for marker-based selection.

**Molecular Marker:** A DNA sequence with a known location on a chromosome used to identify individuals carrying specific alleles or genomic regions.

**SNP (Single Nucleotide Polymorphism):** A single base-pair variation in the DNA sequence used as a molecular marker in genotyping.

**QTLs (Quantitative Trait Loci):** In plant genetics and breeding, QTLs (Quantitative Trait Loci) are genomic regions that contain one or more genes influencing a quantitative trait — a trait that varies continuously and is controlled by multiple genes, such as yield, stress tolerance, or plant height.

In our experiment, the ozone tolerance QTLs (OzT8 and OzT9) are the specific genomic regions identified in the donor parent Kasalath that contribute to enhanced tolerance against ozone stress. According to Frei et al. (2008), the RFLP marker intervals defining these target regions encompass 1.17 Mb (9.63–10.80 Mb on chromosome 9) for the *OzT9* region and 1.07 Mb (23.11–24.18 Mb on chromosome 8) for the *OzT8* region.

These QTLs are being introgressed into the recurrent parents BRRI dhan28 and Binadhan-11 through marker-assisted backcrossing, with the goal of developing ozone-tolerant near-isogenic lines (NILs) that retain the high yield potential and desirable agronomic traits of the recurrent parents while exhibiting improved performance under ozone-stressed environments.

**Flanking Markers:** Molecular markers located on either side of a target gene or QTL, used to detect recombination events during recombinant selection.

**Donor parent:** In plant breeding, a donor parent is the parent that contributes a specific desirable gene or trait (such as disease resistance, ozone tolerance, drought tolerance, or high yield) to another parent (called the recurrent parent) through hybridization or backcrossing. For example, in our experiment, Kasalath serves as the donor parent, as it is the source of the ozone tolerance QTLs (*OzT8*, *OzT9*).

**Recurrent parent: T**he recurrent (or recipient) parent is the elite or well-adapted variety that is repeatedly used in backcrossing to recover most of its genetic background while incorporating a specific desirable gene or QTL from a donor parent. For example, in our experiment, BRRI dhan28 and Binadhan-11 serve as the recurrent/recipient parents. Both are high-yielding modern rice varieties; however, they lack the ozone tolerance QTLs, which makes them more susceptible to ozone stress and results in yield reduction under polluted air conditions. To address this limitation, we aim to introgress the ozone tolerance QTLs (*OzT8*, *OzT9*) from Kasalath into BRRI dhan28 and/or Binadhan-11, so that these improved lines can maintain higher productivity in ozone-stressed environments.

**Introgression:** The process of transferring a gene or QTL from one genetic background (donor) into another (recurrent) through repeated backcrossing.

**Backcrossing:** Backcrossing is a breeding method in which a hybrid offspring (F₁ or later generation) is crossed back to one of its parents—usually the recurrent or recipient parent—to recover most of the desirable genetic background of that parent while retaining a specific target gene or QTL from the donor parent.

In our study, backcrossing is used to introduce the ozone tolerance QTLs (*OzT8*, *OzT9*) from the donor parent *Kasalath* into the genetic backgrounds of BRRI dhan28 and Binadhan-11, which are high-yielding but ozone-sensitive varieties. Through successive backcross generations, we aim to develop near-isogenic lines (NILs) that possess the ozone tolerance of Kasalath while maintaining the high yield potential and agronomic traits of BRRI dhan28 or Binadhan-11.

**Backcross Generation (e.g., BC₁F₁, BC₂F₁):** A generation obtained after crossing the hybrid progeny back to the recurrent parent; the subscript indicates the number of backcrosses.

**Selfing:** The process of allowing a plant to fertilize itself to produce homozygous lines, often used after backcrossing to fix the target allele.

**Marker-Assisted Selection (MAS):** In plant breeding, Marker-Assisted Selection (MAS) is a technique that uses molecular markers linked to specific genes or QTLs to identify and select individuals carrying desirable alleles for targeted traits, thereby increasing selection accuracy and efficiency compared to conventional phenotypic selection.

In our experiment, MAS was employed to introgress and select the ozone tolerance QTLs *OzT8* and *OzT9* from the donor parent Kasalath into the genetic backgrounds of the recurrent parents BRRI dhan28 and Binadhan-11. Using KASP markers linked to these QTLs, we performed foreground selection to identify plants carrying the desired ozone tolerance alleles, recombinant selection to minimize donor genome segments around the target loci, and background selection to recover the maximum proportion of the recurrent parent genome.
Through this MAS-based approach, we aim to develop ozone-tolerant near-isogenic lines (NILs) that combine the tolerance of Kasalath with the high yield potential and agronomic superiority of BRRI dhan28 and Binadhan-11.

**Marker-assisted backcross (MAB) breeding:** Marker-assisted backcross (MAB) breeding is an advanced form of backcrossing that uses molecular markers linked to target genes or QTLs to select and transfer specific genomic regions from a donor parent into the genetic background of a recurrent parent with greater precision and efficiency.

In our study, MABB was employed to introgress the ozone tolerance QTLs (*OzT8*, *OzT9*) from the donor parent *Kasalath* into the high-yielding but ozone-sensitive rice varieties BRRI dhan28 and Binadhan-11. Molecular markers tightly linked to these ozone tolerance QTLs were used to identify and select plants carrying the desired alleles at each backcross generation. This approach accelerated the recovery of the recurrent parent genome while ensuring the successful incorporation of the target QTLs. The ultimate goal is to develop ozone-tolerant near-isogenic lines (NILs) that combine the ozone tolerance of Kasalath with the high yield and adaptability of BRRI dhan28 and Binadhan-11.

**KASP (Kompetitive Allele-Specific PCR) markers:** In molecular breeding, KASP (Kompetitive Allele-Specific PCR) markers are fluorescence-based genotyping tools used to detect specific single nucleotide polymorphisms (SNPs) or small insertions/deletions. They provide a rapid, accurate, and cost-effective method for distinguishing alleles at target loci.

In our study, KASP markers were utilized for both foreground and background genotyping. For foreground selection, KASP markers linked to the ozone tolerance QTLs (*OzT8*, *OzT9*) were used to identify plants carrying the desired alleles from the donor parent Kasalath. For background selection, a set of genome-wide KASP markers was used to estimate the recovery of the recurrent parent genome in BRRI dhan28 and Binadhan-11 during successive backcross generations. This dual application of KASP markers enabled precise introgression of ozone tolerance alleles while accelerating the recovery of the recurrent parent genetic background.

**Foreground selection:** In marker-assisted backcross breeding, foreground selection refers to the process of identifying and selecting plants that carry the target gene or QTL from the donor parent using molecular markers tightly linked to that trait. This ensures that the desired alleles are successfully transferred into the genetic background of the recurrent parent.

In our experiment, foreground selection was performed using KASP markers associated with the ozone tolerance QTLs (*OzT8*, *OzT9*) to identify plants that had successfully inherited these QTLs from the donor parent Kasalath. This step was crucial for confirming the presence of ozone tolerance alleles in each backcross generation before proceeding to background selection.

**Recombinant selection:** In marker-assisted backcross breeding, recombinant selection involves using molecular markers flanking a target gene or QTL to identify crossover events that minimize the donor parent genome segment around the target locus. This helps to reduce linkage drag—the unintentional transfer of undesirable donor genomic regions—while retaining the desired gene or QTL.

In our experiment, recombinant selection was applied using flanking KASP markers around the ozone tolerance QTLs (*OzT8*, *OzT9*) introgressed from the donor parent Kasalath. By selecting plants showing recombination events near these QTL regions, we aimed to narrow down the donor genomic segment carrying the ozone tolerance alleles, ensuring that the resulting lines closely resembled the recurrent parents (BRRI dhan28 and Binadhan-11) in their overall genetic background, but retained the desired ozone tolerance traits.

**Background selection:** In marker-assisted backcross breeding, background selection is the process of using molecular markers distributed across the genome to identify and select plants that have recovered the maximum proportion of the recurrent parent genome, except for the target gene or QTL region transferred from the donor parent. This accelerates the recovery of the recurrent parent’s genetic background compared to conventional backcrossing.

In our experiment, background selection was carried out using genome-wide KASP markers to estimate and select individuals with the highest genetic similarity to the recurrent parents, BRRI dhan28 and Binadhan-11, while retaining the ozone tolerance QTLs (*OzT8*, *OzT9*) from the donor parent Kasalath. This approach helped to efficiently develop near-isogenic lines (NILs) that combine the ozone tolerance of Kasalath with the superior agronomic performance of BRRI dhan28 and Binadhan-11.

**Linkage Drag:** The unintentional transfer of undesirable donor genomic segments along with the target gene/QTL due to tight genetic linkage.

**Fixation:** In marker-assisted backcross breeding, fixation refers to the process of obtaining homozygous plants that carry the desired tolerant alleles at the target QTLs after selfing or advancing the selected backcross generations. This ensures that the target trait is stably inherited in subsequent generations.

In our experiment, fixation involved selfing the selected backcross plants carrying the ozone tolerance QTLs (*OzT8*, *OzT9*) introgressed from the donor parent Kasalath into the recurrent parents BRRI dhan28 and Binadhan-11. The goal was to obtain homozygous lines (near-isogenic lines, NILs) that consistently express ozone tolerance, while maintaining the high yield and agronomic characteristics of the recurrent parents.

**Genome recovery (%):** In marker-assisted backcross breeding, genome recovery (%) refers to the proportion of the recurrent parent genome recovered in a backcross-derived plant, estimated using molecular markers distributed across the genome. It indicates how closely a selected plant resembles the recurrent parent at the genetic level, apart from the target QTL region introgressed from the donor parent.

In our experiment, genome recovery (%) was calculated as:

$$\text{Genome recovery (\%)}=\frac{\text{Number of markers matching recurrent parent}}{\text{Total number of markers used}}\times100$$

Using genome-wide KASP markers, this parameter was employed to identify plants with the highest proportion of the BRRI dhan28 or Binadhan-11 genome while retaining the ozone tolerance QTLs (*OzT8*, *OzT9*) from Kasalath. This helped select the most advanced backcross lines for developing ozone-tolerant near-isogenic lines (NILs).

**Near-Isogenic Line (NIL):** A line developed through repeated backcrossing that is genetically identical to the recurrent parent except for the target gene or QTL region introgressed from the donor parent.

**3. Plant materials:** BRRI dhan28, Binadhan‑11 (recurrent), Kasalath (donor).

Landrace **(-)**

Tall plant & lodging **(-)**

Severe grain awns **(-)**

Red grain pericarp **(-)**

Ozone tolerance QTLs (OzT8 and OzT9) **(+)**


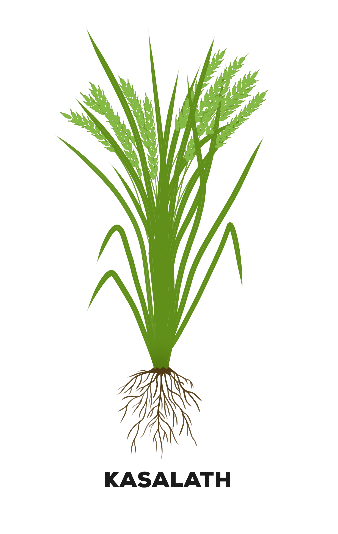


**Kasalath
(Donor parent)**

High-yielding modern variety **(+)**

Semi-dwarf plant type, resistant to lodging **(+)**

Good grain quality **(+)**

Ozone-sensitive  **(–)**


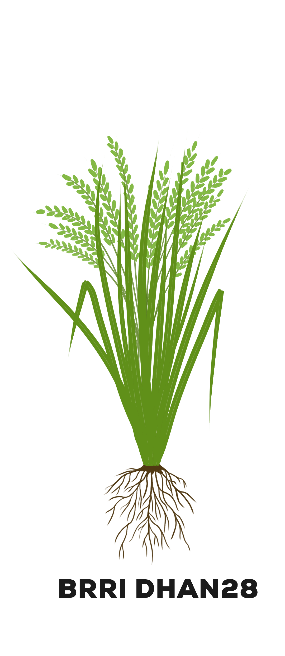


**BRRI dhan28**

**(Recurrent parent)**

High-yielding modern variety **(+)**

Semi-dwarf plant type, resistant to lodging **(+)**

Contains the Sub1 gene, providing submergence tolerance **(+)**

Good grain quality **(+)**

Ozone-sensitive  **(–)**


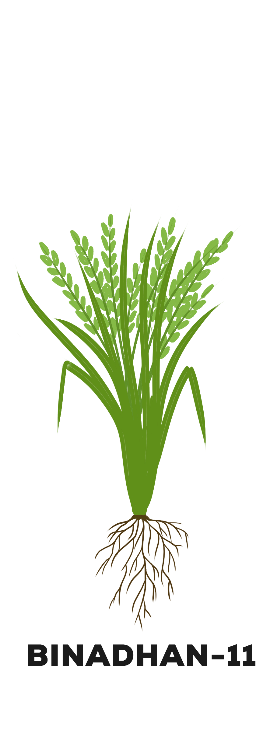


**Binadhan-11**

**(Recurrent parent)**

**Figure 1.** Plant materials used in marker-assisted breeding for ozone tolerance. Kasalath (donor parent) carries ozone tolerance QTLs *OzT8* and *OzT9*, while BRRI dhan28 and Binadhan-11 (recurrent parents) are high-yielding, semi-dwarf varieties; Binadhan-11 additionally carries the Sub1 gene for submergence tolerance.

**4. KASP marker development**

We followed the following steps to develop KASP (Kompetitive Allele-Specific PCR) markers linked to the ozone tolerance QTLs *OzT8* and *OzT9* (Figure 2):

1. **Target identification:**
   We identified the ozone tolerance QTLs *OzT8* (23.11–24.18 Mb on chromosome 8) and *OzT9* (9.63–10.80 Mb on chromosome 9) as reported by Frei et al. (2008) and selected these regions for marker development for foreground selection.
2. **Genotyping-by-sequencing (GBS):**

We outsourced genotyping-by-sequencing (GBS) to the Japan International Research Center for Agricultural Sciences (JIRCAS), Japan, to identify genome-wide single nucleotide polymorphisms (SNPs) between the donor parent (*Kasalath*) and the recurrent parents (BRRI dhan28 and Binadhan-11).

1. **Data quality control:**
   We filtered raw sequence reads to remove low-quality bases, adapters, and ambiguous reads. Only high-quality, biallelic SNPs were retained for further analysis.
2. **Alignment to the reference genome:**

We aligned the cleaned reads to the Nipponbare reference genome and identified polymorphic SNPs and InDels between *Kasalath* and the recurrent parents, focusing on the *OzT8* and *OzT9* intervals.


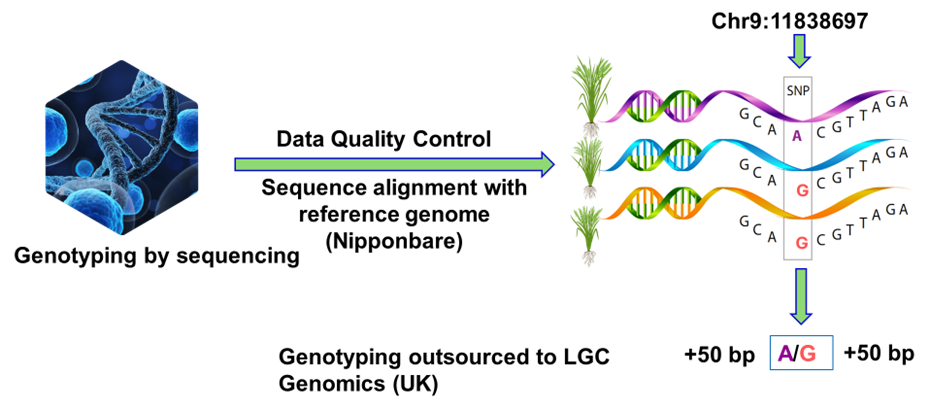


**Figure 2.** Schematic representation of the stepwise process used to develop KASP (Kompetitive Allele-Specific PCR) markers for ozone tolerance QTLs. Genotyping-by-sequencing (GBS) data from *Kasalath*, BRRI dhan28, and Binadhan-11 were subjected to data quality control and aligned to the *Oryza sativa* reference genome (Nipponbare) to identify polymorphic SNPs. Candidate SNPs with clean ±50 bp flanking regions were selected for KASP assay design. Genotyping was outsourced to LGC Genomics (UK).

1. **Selection of polymorphic SNPs:**

We selected SNPs that showed clear allele differentiation between the donor and recurrent parents and had clean flanking sequences (±50 bp) suitable for primer design. One example SNP was located at Chr9:11838697 (A/G).

1. **Design of KASP assays:**
   For each selected SNP, we designed KASP markers following the assay design guidelines provided by LGC Genomics (Biosearch Technologies). Detailed instructions and design principles are available in the following official resources:

KASP assay design for genotyping projects:
<https://www.biosearchtech.com/support/resources/starting-a-genotyping-project/assay-design-for-kasp-chemistry>

KASP assay design factsheet (PDF):
<https://biosearchassets.blob.core.windows.net/assetsv6/factsheet_kasp-assay-design.pdf>

KASP assay design anchoring guide (PDF):
<https://biosearchassets.blob.core.windows.net/assetsv6/guide_kasp-assay-design-anchoring.pdf>

**5. Application of KASP markers in marker-assisted backcross breeding:**

A schematic representation of the overall breeding and marker-assisted selection process is presented in Figure 1 of the main manuscript. In total, 48 KASP assays (RIOZ_101–RIOZ_148) were synthesized (details provided in *Supplementary Table S1*) and genotyped by LGC Genomics (UK) using DNA from *Kasalath*, BRRI dhan28, Binadhan-11, and their respective breeding lines ranging from BC₁F₁ to BC₂F₄ and BC₃F₃ generations (final genotyping results are shown in *Table 2* of the main manuscript).

We extracted genomic DNA from young leaf tissues of individual plants at each backcross generation and sent the samples to LGC Genomics (UK) for KASP genotyping. The validated KASP markers were subsequently used in three key selection stages of the marker-assisted backcross breeding (MABB) program:

**Foreground selection:**

For each QTL (*OzT8* and *OzT9*), we designed one KASP marker at the left border and another at the right border of the target region (a total of four KASP markers). Foreground selection was conducted from BC₁F₁ to BC₂F₃ and BC₃F₁ to BC₃F₂ generations to identify plants carrying the *Kasalath* alleles for ozone tolerance.

**Recombinant selection:**

For each QTL, we selected two flanking SNPs on each side (upstream and downstream) located approximately 1 Mb from the QTL borders. These markers were used to detect crossover events and minimize linkage drag. Recombinant selection was performed from BC₁F₁ to BC₂F₃ and BC₃F₁ to BC₃F₂ generations.

**Background selection:**

To recover the recurrent parent genome, we developed a 41-marker KASP panel evenly distributed across all 12 chromosomes for genome-wide coverage. Background selection was performed in the BC₂F₄ and BC₃F₃ generations (where applicable) to estimate genome recovery (%) and select lines genetically closest to the recurrent parents while retaining the target QTLs.

**7. Step by step breeding procedures with KASP genotyping**

We implemented a marker-assisted backcross (MAB) breeding approach to introgress the ozone tolerance QTLs *OzT8* and *OzT9* from the donor parent *Kasalath* into two high-yielding but ozone-sensitive Bangladeshi indica rice varieties: BRRI dhan28 and Binadhan-11.
Two independent breeding schemes were developed:

- **Scheme A:** BRRI dhan28 × Kasalath
- **Scheme B:** Binadhan-11 × Kasalath

**Step 1: Development of Parental Crosses (F₁ Generation)**

We used BRRI dhan28 and Binadhan-11 as female parents and Kasalath as the male parent. Controlled hand pollinations were performed to produce F₁ seeds (Figure 3). The resulting F₁ plants were verified by phenotypic observation, as they were clearly distinguishable from their respective recurrent parents in several morphological traits, confirming successful hybridization.


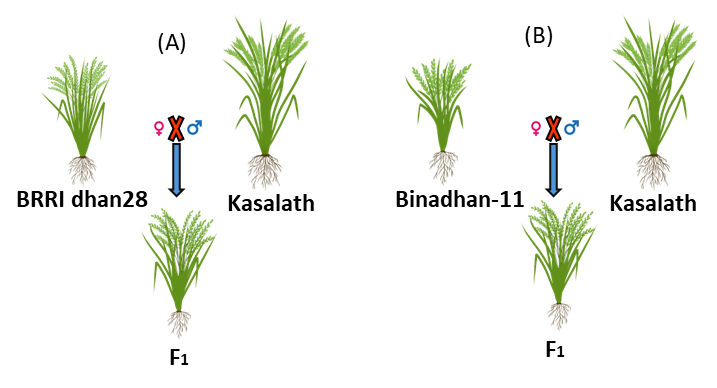


**Figure 3.** Development of F₁ Generation

**Step 2: First backcross (BC₁F₁ generation)**

The confirmed F₁ plants were backcrossed with their respective recurrent parents (*BRRI dhan28* or *Binadhan-11*) to generate BC₁F₁ progeny (Figure 4).

We genotyped 218 BC₁F₁ lines (135 from cross A and 83 from cross B) using the following marker sets:

- **Foreground selection:**

We used one KASP marker at the left border and one at the right border of each QTL (*OzT8* and *OzT9*) to identify plants carrying the *Kasalath* alleles.

- **Recombinant selection:**

We used two flanking KASP markers positioned approximately 1 Mb away from each border (upstream and downstream) to detect recombination events and minimize donor segment size.

Selected BC₁F₁ plants carrying QTL (*OzT8* and *OzT9*) were selected for further backcrossing.


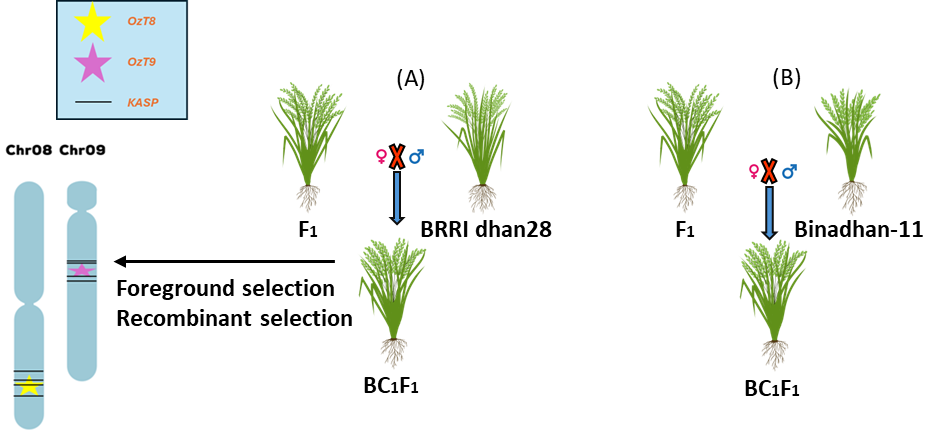


**Figure 4.** First backcross to generate BC₁F₁ generation and KASP genotyping for foreground and background selection (Genotyping was done for both cross schemes A & B). Chromosome diagrams highlight the positions of KASP markers, with *OzT8* (yellow star), and *OzT9* (pink star) explicitly indicated.

**Step 3: Second backcross (BC₂F₁ generation)**

The selected BC₁F₁ plants were again backcrossed to their respective recurrent parents to produce BC₂F₁ progeny (Figure 5).

We genotyped 1,034 BC₂F₁ lines (551 from BRRI dhan28 × Kasalath and 483 from Binadhan-11 × Kasalath) using the same marker sets as in BC₁F₁.

Foreground and recombinant selections were repeated to identify plants carrying either *OzT8*, *OzT9*, or both.

In addition to genotypic screening, we evaluated phenotypic traits such as plant height, tiller number, panicle structure, grain size, awn presence, and pericarp color to eliminate plants expressing *Kasalath*-like traits (e.g., tall, awned, red pericarp).


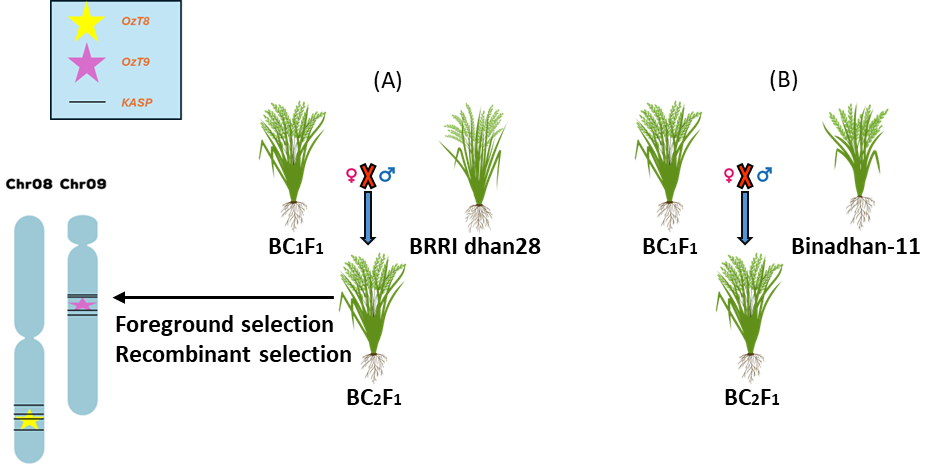


**Figure 5.** Second backcross to generate BC_2_F₁ generation and KASP genotyping for foreground and background selection (Genotyping was done for both cross schemes A & B). Chromosome diagrams highlight the positions of KASP markers, with *OzT8* (yellow star), and *OzT9* (pink star) explicitly indicated.

**Step 4: Third backcross (BC₃F₁ generation)**

A subset of selected BC₂F₁ plants with ideal genotypic and phenotypic characteristics were further backcrossed to generate BC₃F₁ progeny (Figure 6).

Foreground and recombinant selections were performed as before.


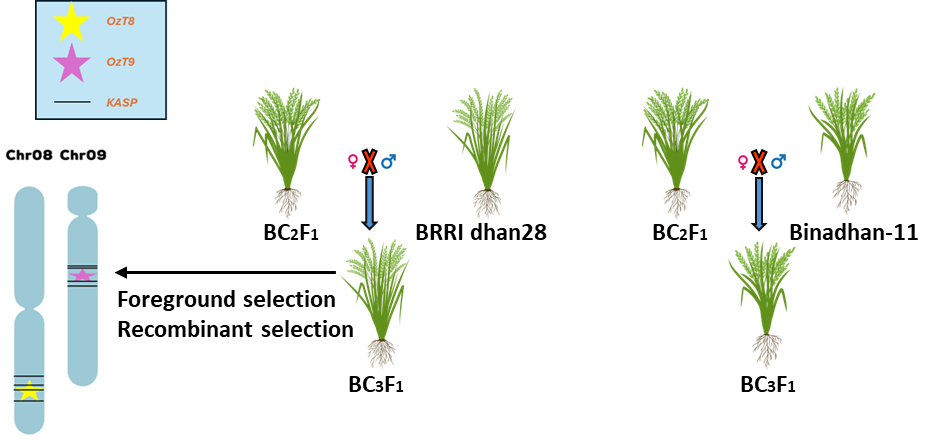


**Figure 6.** Third backcross to generate BC_3_F₁ generation and KASP genotyping for foreground and background selection (Genotyping was done for both cross schemes A & B). Chromosome diagrams highlight the positions of KASP markers, with *OzT8* (yellow star), and *OzT9* (pink star) explicitly indicated.

**Step 5: Selfing and advancement (BC₂F₂ – BC₃F_2_ generations)**

We self-pollinated selected BC₂F₁ and BC₃F₁ plants to advance the generations (Figure 7).
During these stages, we planted and genotyped:

- 2,300 BC₂F₂ lines (1,378 from cross A; 922 from cross B)
- 46 BC₃F₁ lines (25 from cross A; 21 from cross B)

Foreground and recombinant selections were repeated in each generation using the previously developed KASP markers.

Lines carrying homozygous tolerant alleles (AA) at one or both QTLs were advanced to the next generation.


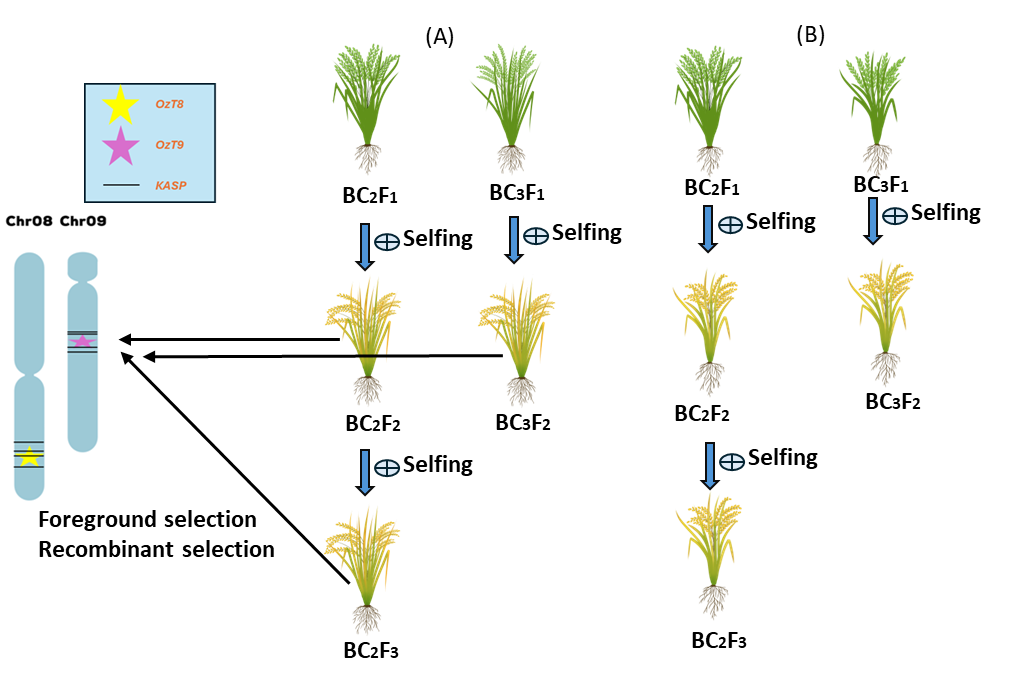


**Figure 7.** Line advancement by selfing and KASP genotyping for foreground and background selection (Genotyping was done for both cross schemes A & B). Chromosome diagrams highlight the positions of KASP markers, with *OzT8* (yellow star), and *OzT9* (pink star) explicitly indicated.

**Step 6: Background selection and genome recovery**

To accelerate the recovery of the recurrent parent genome, we developed a 41-marker KASP panel distributed across all 12 rice chromosomes for genome-wide background selection (Figure 8).

This genotyping was conducted on BC₂F₄ and BC₃F₃ generations. We calculated Genome Recovery (%) using the following formula:

$$\text{Genome Recovery (\%)}=(\frac{\text{Number of markers matching the recurrent parent}}{\text{Total number of markers tested}})\times100$$

Lines with the highest genome recovery and fixed tolerant alleles were selected as Near-Isogenic Lines (NILs).


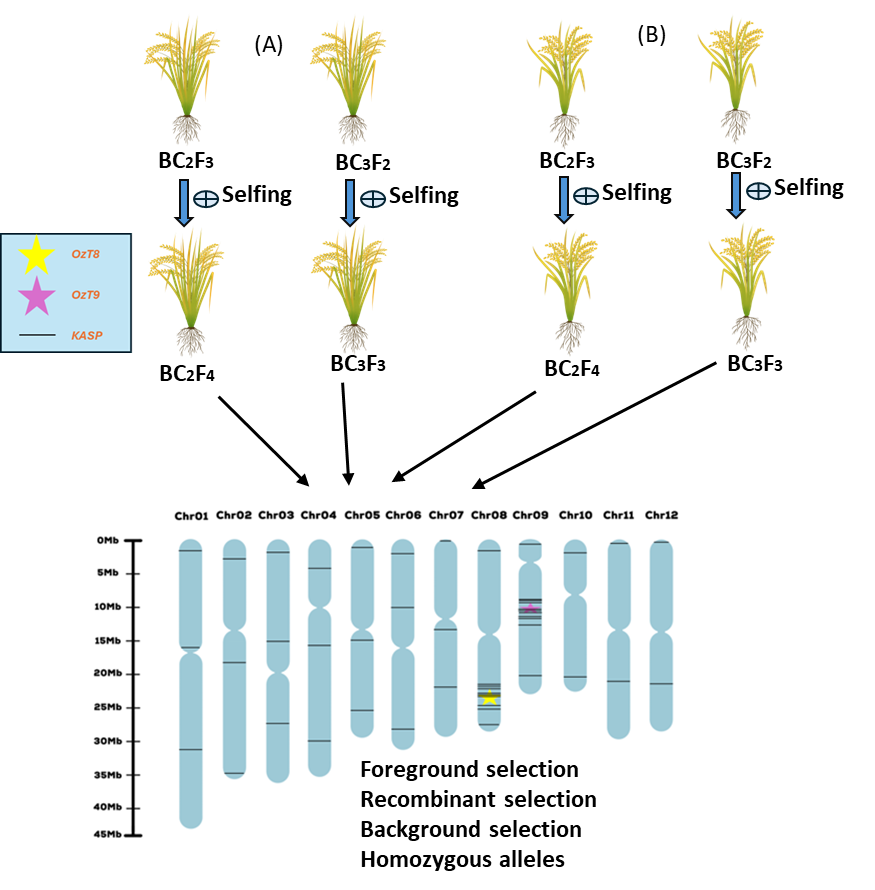


**Figure 8.** Background selection and genome recovery (Genotyping was done for both cross schemes A & B). Chromosome diagrams highlight the positions of KASP markers, with *OzT8* (yellow star), and *OzT9* (pink star) explicitly indicated.

**Step 7: Final confirmation and phenotyping**

We conducted preliminary ozone-stress phenotyping using these NILs to evaluate tolerance levels and agronomic performance under controlled ozone exposure.

Lines exhibiting both high genome recovery and superior ozone tolerance were identified as candidate NILs for further evaluation.

**Outcome**

Through this stepwise marker-assisted backcrossing approach, we successfully developed advanced breeding lines in the genetic backgrounds of BRRI dhan28 and Binadhan-11, each carrying *OzT8*, *OzT9*, or both QTLs, fixed for the *Kasalath* alleles, and with genome recovery exceeding 82 to 90%. These lines represent ozone-tolerant near-isogenic lines (NILs) with agronomic characteristics similar to the recurrent parents.
